# The molecular basis of octocoral calcification revealed by genome and skeletal proteome analyses

**DOI:** 10.1093/gigascience/giaf031

**Published:** 2025-04-01

**Authors:** Yanshuo Liang, Kuidong Xu, Junyuan Li, Jingyuan Shi, Jiehong Wei, Xiaoyu Zheng, Wanying He, Xin Zhang

**Affiliations:** Laboratory of Marine Organism Taxonomy and Phylogeny, Qingdao Key Laboratory of Marine Biodiversity and Conservation, Institute of Oceanology, Chinese Academy of Sciences, Qingdao 266071, China; Laoshan Laboratory, Qingdao 266237, China; University of Chinese Academy of Sciences, Beijing 100049, China; Laboratory of Marine Organism Taxonomy and Phylogeny, Qingdao Key Laboratory of Marine Biodiversity and Conservation, Institute of Oceanology, Chinese Academy of Sciences, Qingdao 266071, China; Laoshan Laboratory, Qingdao 266237, China; University of Chinese Academy of Sciences, Beijing 100049, China; Laboratory of Marine Organism Taxonomy and Phylogeny, Qingdao Key Laboratory of Marine Biodiversity and Conservation, Institute of Oceanology, Chinese Academy of Sciences, Qingdao 266071, China; Laoshan Laboratory, Qingdao 266237, China; CAS Key Laboratory of Marine Ecology and Environmental Sciences, Institute of Oceanology, Chinese Academy of Sciences, Qingdao 266071, China; Laboratory of Marine Organism Taxonomy and Phylogeny, Qingdao Key Laboratory of Marine Biodiversity and Conservation, Institute of Oceanology, Chinese Academy of Sciences, Qingdao 266071, China; Laoshan Laboratory, Qingdao 266237, China; University of Chinese Academy of Sciences, Beijing 100049, China; Laboratory of Marine Organism Taxonomy and Phylogeny, Qingdao Key Laboratory of Marine Biodiversity and Conservation, Institute of Oceanology, Chinese Academy of Sciences, Qingdao 266071, China; Laoshan Laboratory, Qingdao 266237, China; University of Chinese Academy of Sciences, Beijing 100049, China; University of Chinese Academy of Sciences, Beijing 100049, China; Key Laboratory of Marine Geology and Environment & Center of Deep Sea Research, Institute of Oceanology, Chinese Academy of Sciences, Qingdao 266071, China; University of Chinese Academy of Sciences, Beijing 100049, China; Key Laboratory of Marine Geology and Environment & Center of Deep Sea Research, Institute of Oceanology, Chinese Academy of Sciences, Qingdao 266071, China

**Keywords:** Octocorallia, genomes, CaCO_3_ polymorphs, skeletal proteomes, biomineralization toolkit

## Abstract

The ability of octocorals and stony corals to deposit calcium carbonate (CaCO_3_) has contributed to their ecological success. Whereas stony corals possess a homogeneous aragonite skeleton, octocorals have developed distinct skeletal structures composed of different CaCO_3_ polymorphs and a skeletal organic matrix. Nevertheless, the molecular basis of skeletal structure formation in octocorals remains inadequately understood. Here, we sequenced the genomes and skeletal proteomes of two calcite-forming octocorals, namely *Paragorgia papillata* and *Chrysogorgia* sp. The assembled genomes sizes were 618.13 Mb and 781.04 Mb for *P. papillata* and *Chrysogorgia* sp., respectively, with contig N50s of 2.67 Mb and 2.61 Mb. Comparative genomic analyses identified 162 and 285 significantly expanded gene families in the genomes of *P. papillata* and *Chrysogorgia* sp., respectively, which are primarily associated with biomineralization and immune response. Furthermore, comparative analyses of skeletal proteomes demonstrated that corals with different CaCO_3_ polymorphs share a fundamental toolkit comprising cadherin, von Willebrand factor type A, and carbonic anhydrase domains for calcified skeleton deposition. In contrast, collagen is abundant in the calcite-forming octocoral skeletons but occurs rarely in aragonitic stony corals. Additionally, certain collagens have developed domains related to matrix adhesion and immunity, which may confer novel genetic functions in octocoral calcification. These findings enhance our understanding of the diverse forms of coral biomineralization processes and offer preliminary insights into the formation and evolution of the octocoral skeleton.

## Introduction

The history of calcium carbonate (CaCO_3_) biomineralization by organisms spans at least 541 Myr, and biomineralization as an innovative mechanism in the evolutionary history of life has played a significant role in species development and global carbon cycles [1]. The class Anthozoa, an ecologically important and morphologically diverse clade of metazoans, produces extensive biogenic structures through their ability to form colonies, and precipitates CaCO_3_ skeletons to support entire coral ecosystems in both shallow and deep waters. The ability to produce CaCO_3_ skeletons is found in two distinct clades of Anthozoa, namely the order Scleractinia (stony coral, subclass Hexacorallia) and the subclass Octocorallia (octocoral). As the primary reef builders, stony corals possess homogeneous aragonite skeletons, and their calcification process has been elucidated through skeletal proteome analysis and immunohistochemical verification [2–5]. In contrast, octocorals have evolved diverse skeletal structures, mainly including different CaCO_3_ polymorphs (i.e., aragonite or calcite) and organic components (e.g., gorgonin) as well as different types of sclerites [6, 7]. Thus, skeletons of octocorals provide a unique opportunity to compare different calcification strategies involving varied skeletal structures and CaCO_3_ polymorphs with those of stony corals.

The formation of coral skeletons is biologically controlled by the supply of ions required for CaCO_3_ deposition and the secretion of a diverse organic matrix at the site of calcification (Supplementary Fig. S1) [8–10]. Major components of the organic matrix include proteins, carbohydrates, and lipids [6, 8]. Despite comprising a minimal portion of the coral skeletal organic matrix space, the organic matrices secreted by the calicoblastic ectoderm play an important role in promoting nucleation, growth, and spatial orientation of various CaCO_3_ polymorphs [11]. A previous study showed that, although mollusks possess a set of conserved biomineralization-related proteins, the calcite and aragonitic layers within the shell use specific shell matrix proteins to deposit different polymorphs [12]. A fundamental question related to coral calcification is elucidating the mechanisms by which corals regulate calcite and aragonitic polymorphs through skeletal organic matrix proteins (SOMPs), and how they control the development of complex and diverse skeletal structures. However, the lack of comprehensive genomic and proteomic data for octocorals has constrained our understanding of the molecular mechanisms underlying the formation of skeletal structures with different CaCO_3_ polymorphs.

In the present study, we generated draft genomes of two calcite-forming octocorals *Paragorgia papillata* (NCBI: txid2853639; marinespecies.org:taxname:1545268) and *Chrysogorgia* sp. (NCBI: txid3051262) and characterized their skeletal proteomes. We further performed comprehensive phylogenetic analyses, gene family evolution studies, and comparative skeletal proteomic analyses to understand the molecular mechanisms of skeleton formation in octocorals. The obtained genome and proteome information can contribute significantly to our understanding of the molecular mechanisms of coral skeletal formation and its evolutionary development.

## Methods

### Sample collection and DNA extraction

**Figure 1:**
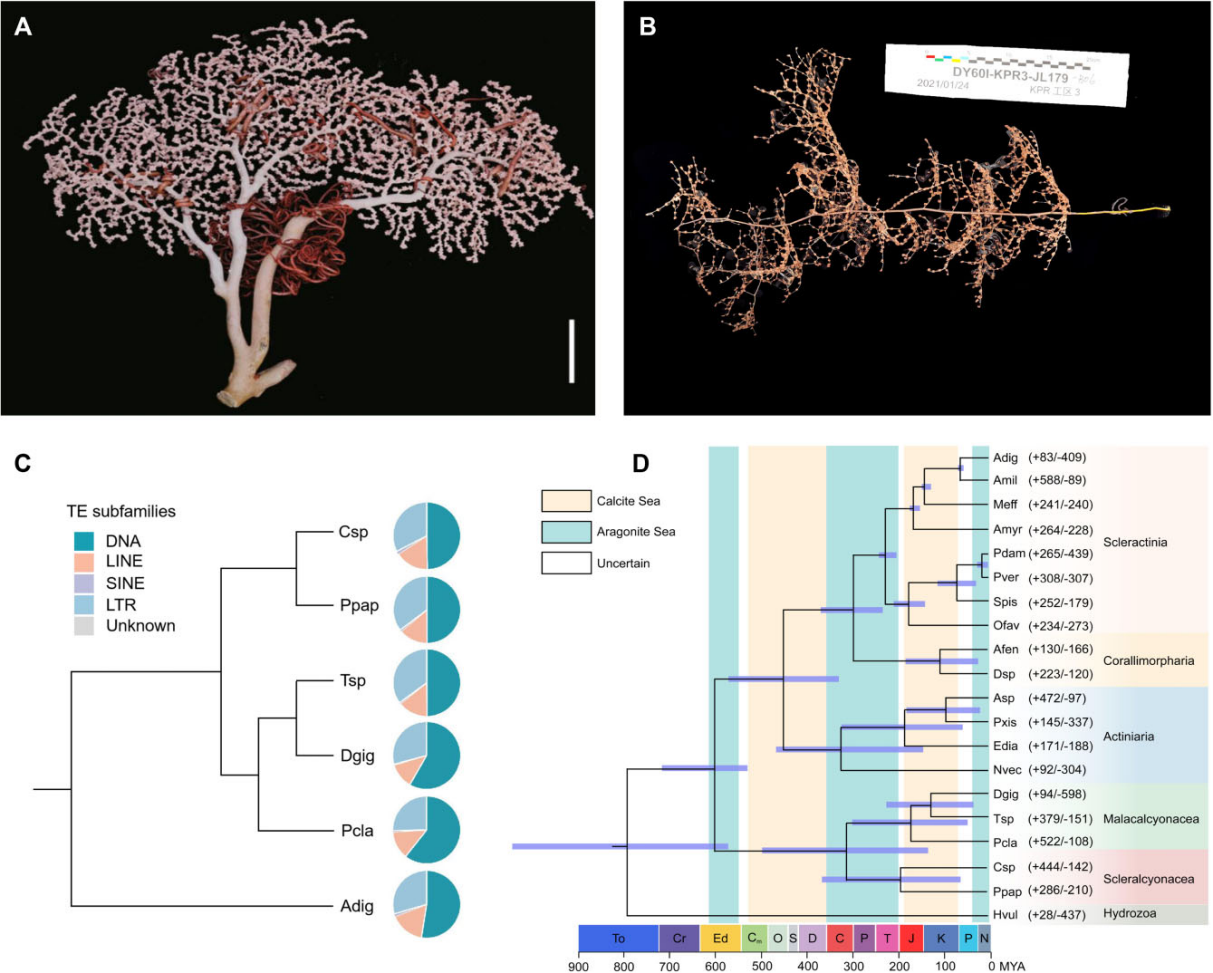
Evolution of the genomes of *P. papillata* and *Chrysogorgia* sp. (a,b) Freshly collected samples of *P. papillata* (a) and *Chrysogorgia* sp. (b). Scale bar (a): 10 cm. (c) Proportions of DNA transposons and LTR, LINE, and SINE retrotransposons in the genomes of 6 representative anthozoans including *P. papillata* (Ppap), *Chrysogorgia* sp. (Csp), *D. gigantea* (Dgig), *P. clavata* (Pcla), *Trachythela* sp. (Tsp), and *A. digitifera* (Adig). The tree illustrates the evolutionary relationships among the 6 corals. The pie charts are scaled according to the genome size (Supplementary Table S9). (d) A phylogenetic tree was constructed using 275 single-copy orthologues from 19 anthozoans and *Hydra vulgaris* (outgroup). Divergence time was estimated with the approximate likelihood calculation method together with a correlated rates molecular clock. The 95% confidence interval of the estimated divergence time at each node is denoted as a blue bar. The positive and negative numbers adjacent to the species abbreviations represent gene family numbers of expansion/contraction derived from the CAFE analysis. Species abbreviations in Supplementary Table S1. Geological era abbreviations: To: Tonian; Cr: Cryogenian; Ed: Ediacaran; Cm: Cambrian; O: Ordovician; S: Silurian; D: Devonian; C: Carboniferous; P: Permian; T: Triassic; J: Jurassic; K: Cretaceous; P: Palaeogene; N: Neogene.

Samples of *P. papillata* and *Chrysogorgia* sp. were collected using the submersible vehicles *Faxian* and *Jiaolong* from seamounts of the Caroline Ridge (10°06′46.80″N, 140°14′31.79″E, 858 m deep) and the Kyushu-Palau Ridge (13°20′18.24″N, 134°33′37.44″E, 2,086 m deep) in the tropical Western Pacific (Fig. 1a,b). The coral samples were preserved in a sealed sample chamber placed inside the sample basket of the submersible. Following recovery, the samples were sectioned into small pieces and immediately preserved in liquid nitrogen. All experimental protocols were approved by the relevant guidelines and regulations established by the Institutional Animal Care and Use Committee of the Institute of Oceanology, Chinese Academy of Sciences. Genomic DNA was extracted from the polyps by using the MagAttract HMW DNA kit (Qiagen, Stuttgart, Germany). The quality and quantity of the extracted DNA were validated with standard agarose gel electrophoresis and a Qubit Fluorometer, respectively.

### Illumina sequencing and genome size estimation

Paired-end libraries with insert sizes of 300 bp were constructed using the TruSeq DNA Sample Prep Kit in accordance with the manufacturer’s instructions. The resulting libraries were then sequenced on an Illumina NovaSeq 6000 platform (RRID:SCR_016387). Low-quality reads and sequencing-adapter-contaminated reads were trimmed using Trimmomatic-0.36 (RRID:SCR_011848). A *k*-mer frequency distribution map of the clean reads was constructed to estimate the genome size, heterozygosity, and proportion of repetitive sequences by using the Jellyfish v.2.2.7 (RRID:SCR_005491) [13]. The genome size (*G*) was calculated using the following formula: *G* = K_num_/*K*_depth_, where *K*_num_ is the number of *k*-mers and *K*_depth_ is the peak depth. The trimmed Illumina paired-end reads were assembled into scaffolds using SOAPdenovo v.2.04 (RRID:SCR_010752) [14] with the following specified parameters: -K 45 -d 1 -D 1 -F.

### PacBio sequencing and genome assembly

High-molecular-weight genomic DNA (gDNA) was used for constructing Pacific Biosciences (PacBio) sequencing libraries. The gDNA was fragmented with the g-TUBE device (Covaris) to achieve a size range of 6–20 kb to construct 20 kb libraries. The fragmented DNA was then concentrated and purified using AMPure XP beads (Agencourt). The SMRTbell Template Prep Kit reagents were used to repair various DNA damage, including abasic sites, nicks, thymine dimers, blocked 3ʹ-ends, oxidized guanines/pyrimidines, and deaminated cytosines. T4 DNA polymerase was utilized to polish the ends of the fragments deemed suitable for ligation. The SMRTbell hairpin adapters were then ligated to the repaired ends. Subsequently, size selection was conducted by BluePippin electrophoresis (Sage Science), with a cut-off threshold size of 20 kb. Subsequently, AMPure PB Beads were used to concentrate and purify the SMRTbell templates after size selection. Finally, these purified SMRTbell templates were utilized for primer and polymerase binding. The SMRTbell libraries were then sequenced on a Pacbio Sequel II platform (RRID:SCR_017990).

PacBio long reads were subjected to quality control by using SequelQC software (RRID:SCR_017279), and the clean reads were corrected using the error-correction module of Canu v.1.5 (RRID:SCR_015880) [15] to select longer subreads. Contaminated reads containing chloroplast, mitochondrial, bacterial, or viral sequences were removed through comparison of the genome assembly with the nucleotide sequence database from the National Center for Biotechnology Information (NCBI) using BLASTN v.2.2.26 with an *e*-value threshold of ≤1 × 10^−5^. The data were then assembled using NextDenovo v.2.2 (RRID:SCR_025033) with default parameters. The raw assembly was subjected to 3 rounds of polishing with Illumina short reads using Pilon (RRID:SCR_014731) [16]. Finally, the PacBio reads were aligned to the initial assembly by using minimap2 v.2.24-r1122 (RRID:SCR_018550) with the parameter: -x map-bp. Haplotigs and contig overlaps in the resulting assembly were eliminated using Purge_dups v.1.2.5 (RRID:SCR_021173) [17] with the parameter minimumAlignmentScore 70 for *P. papillata* and minimumAlignmentScore 80 for *Chrysogorgia* sp. To evaluate the accuracy of the genome assembly, the Illumina reads were first mapped to the genome assembly by using bwa v.0.7.10 (RRID:SCR_010910). The completeness of the genome assembly was assessed by mapping 954 metazoan benchmarking universal single-copy orthologues to the genome by using BUSCO v.5.0 (RRID:SCR_015008) [18].

### Transcriptome sequencing

Total RNA was extracted from the polyps of *P. papillata* and *Chrysogorgia* sp. by using Invitrogen TRIzol reagent (Thermo Fisher Scientific) by following the manufacturer’s instructions. The integrity and quality of the extracted RNA were evaluated using a Fragment Analyzer 5400 (Agilent Technologies). Sequencing libraries were generated using the NEBNext UltraTM RNA Library Prep Kit for Illumina (NEB, USA) in accordance with the manufacturer’s instructions, with an insert size of 300–500 bp. Illumina RNA sequencing (RNA-seq) libraries were prepared and sequenced on the Illumina NovaSeq 6000 platform, resulting in 150-bp paired-end reads. After performing quality score-based trimming using Trimmomatic-0.36, the clean reads were aligned to the coral genomes by using StringTie v.2.1.5 (RRID:SCR_016323) [19].

### Genome annotation

The protein-coding genes were annotated through a combination of *ab initio* prediction methods, homology searches, and RNA sequencing. *Ab initio* gene prediction was performed using Augustus v.3.1.0 (RRID:SCR_008417) and SNAP v.2006–07-28 (RRID:SCR_007936) with default parameters. For the homolog-based approach, GeMoMa v.1.7 (RRID:SCR_017646) [20] software was performed by using a reference gene model from a selection of other cnidarians: *Acropora digitifera, Acropora millepora, Astreopora myriophthalma, Dendronephthya gigantea, Porites australiensis, Paramuricea clavata*, and *Stylophora pistillata*. Gene prediction based on the RNA-seq data was conducted by aligning clean RNA-seq reads to the reference genome using Hisat2 v.2.0.4 (RRID:SCR_015530) [21] and assembling them with StringTie v.2.1.5. The coding regions were predicted using GeneMarkS-T v.5.1 (RRID:SCR_017648) [22] and PASA v.2.0.2 (RRID:SCR_014656) [23]. Gene models from these different approaches were integrated using EVM v.1.1.1 with default parameters (RRID:SCR_014659) [24] and updated by PASA. The weights assigned to *ab initio* prediction, protein alignment, and transcript were 4, 7, and 8, respectively. The final gene models were annotated by searching against the GenBank Non-Redundant, Gene Ontology, KEGG, and SwissProt databases, with an *e*-value threshold of 1×10^−5^. Additionally, these predicted genes were annotated against the Pfam database using HMMER v.3.3.2 (RRID:SCR_005305) software.

Transposable elements (TEs) were analyzed using the RepeatModeler pipeline v.2.0.1 (RRID:SCR_015027) [25] and LTR_retriever v.2.9.0 (RRID:SCR_017623) [26]. Initially, RECON v.1.0.8 (RRID:SCR_021170), RepeatScout v.1.0.6 (RRID:SCR_014653), LTRharvest v.1.5.10 (RRID:SCR_018970), and LTR_FINDER v.1.0.7 (RRID:SCR_015247) were utilized to construct a *de novo* repeat library using default parameters. The predicted repeats were classified using RepeatClassifier and integrated with the Dfam database v.3.5. Subsequently, RepeatMasker v.4.1.2 (RRID:SCR_012954) [27] was used to identify the divergence of TEs in the coral genomes based on the constructed repetitive sequence database. The repeat landscape was obtained using a modified R script from GitHub.

### Phylogenetic analysis and gene expansion and contraction

The ortholog groups (OGs) were identified through a BLASTp search of protein sequences from the genomes of 19 anthozoans and *Hydra vulgaris* (outgroup) (Supplementary Table S1). The BLASTp results were used by OrthoFinder v.2.4.0 (RRID:SCR_017118) [28] to construct OGs. To construct phylogenetic relationships, the protein sequences from 275 single-copy orthologs were extracted from all 20 species and analyzed through multiple alignment using MAFFT v.7.310 (RRID:SCR_011811). Subsequently, poorly aligned regions were trimmed using Gblocks v.0.91b (RRID:SCR_015945), and all alignments were combined into one supergene. ModelFinder software was used to identify the optimal model for the trimmed alignment, and the maximum-likelihood tree was generated using IQtree v.2.2.0 (RRID:SCR_017254) [29] with 1,000 bootstrap replicates. The divergence times were estimated using the MCMCTree program from PAML v.4.9j (RRID:SCR_014932) [30] with a correlated rates molecular clock. Five fossil calibration points (Supplementary Table S2) were selected for dating the phylogeny of anthozoans. Finally, the OGs comprising >100 copies in a single species were excluded, and the remaining OGs were used for the gene family expansion and contraction analysis performed with CAFÉ v.4.2.1 (RRID:SCR_005983) [31], with the parameter lambda -s and estimated divergence times between species as the input. An event of significant expansion or contraction was considered only when the gene family-wide *P-*value was <0.01 and the taxon-specific Viterbi *P-*value was <0.05. The significantly expanded and contracted gene families were extracted for the Gene Ontology term enrichment analysis with Fisher's exact test, and the *P-*value was adjusted for multiple testing by using the false discovery rate method.

### Morphological observation, CaCO_3_ polymorph analysis, and van Gieson staining of octocoral skeletons

To observe the skeletal ultrastructure, the axial skeletons of *P. papillata* and *Chrysogorgia* sp. were isolated by digestion of the tissues in a sodium hypochlorite solution and washed repeatedly by multiple rinses in milli-Q water. The axial skeletons were subsequently mounted on carbon double-adhesive tape, air-dried, and coated for scanning electron microscopy (SEM) examination. SEM scans were performed using a Hitachi TM3030Plus scanning electron microscope at 15 kV and optimal magnification for each axial skeleton. To determine the CaCO_3_ polymorphs of coral skeletons, confocal Raman spectroscopy (Alpha 300R+, WITec, Ulm, Germany) was conducted to detect the axial skeleton after the removal of the coenenchyme. Van Gieson (VG) staining was performed to determine the distribution of collagen fibers in axial skeletons. The protocol involved the following steps. First, the decalcified axial skeleton was embedded in paraffin, dewaxed with xylene and ethanol, and stored in tap water. Next, the samples were treated with the VG staining solution (Servicebio) for 1 min, rinsed rapidly with water, and dehydrated rapidly in 3 grades of anhydrous ethanol. Finally, the slides were immersed in xylene until they were transparent, coverslipped with neutral resin, observed under a microscope, and photographed.

### Proteomics analysis

The axial skeletons of *P. papillata* and *Chrysogorgia* sp. were bleached in a 10% hypochlorite solution for 5 h to remove the tissue and other potential contaminants. Subsequently, the skeletons were thoroughly rinsed with milli-Q water and dried overnight at 60°C. The dried axial skeletons were pulverized to a fine powder in liquid nitrogen, and bleached again, rinsed, and dried. The skeleton powder was decalcified with 10% acetic acid for 24 h at room temperature on an orbital shaker, and the decalcified solution was centrifuged (14,000 × *g*, 10 min, 4°C) to separate the acid-soluble matrix (ASM) and acid-insoluble matrix (AIM). The resulting insoluble pellets (AIM) were rinsed repeatedly with milli-Q water, lyophilized, and reconstituted with 8 M urea (with 1% SDS). Both AIM and ASM were concentrated using Amicon Ultrafiltration devices (15 ml, 10 kDa cut-off), purified with methanol/chloroform, and subsequently reconstituted in 8 M urea.

The ASM and AIM samples were dissolved in solubilization buffer (1% SDS, 10 mM DTT, 50 mM Tris–HCl (pH 8.0)) for sodium dodecyl sulfate–polyacrylamide gel electrophoresis. The samples were subsequently prepared for HPLC-MS/MS analysis through a series of steps, including reduction, alkylation, trypsin digestion, drying, and solubilization. Label-free MS was conducted using a Thermo Orbitrap Fusion mass spectrometer. The scan events were configured as a full MS scan in the range of 250–1450 *m*/*z* at a mass resolution of 120,000, followed by CID MS/MS scan repetition on the 20 most abundant ions selected from the previous full MS scan with an isolation window. The resulting MS raw data were imported into MaxQuant v.1.5.2.8 (RRID:SCR_014485) [32] and compared against their respective genomic data. For this study, proteins were considered identified if they exhibited a spectral count exceeding 2 in each sample Identified proteins with at least 2 distinct peptides were considered for the analysis.

Protein annotation was performed based on sequence similarity search against the NR database in NCBI and the UniProtKB/SwissProt database using BLASTP with an *e*-value threshold of 1 × 10^−5^. Protein sequences were analyzed for signal peptides and transmembrane domains by using Signal IP v.5.0 (RRID:SCR_015644) and TMHMM v.2.0 (RRID:SCR_014935), respectively. Conserved domains were detected using the InterproScan platform (RRID:SCR_005829). In previous studies, protein identification was based on matching nucleotides or EST databases with unique peptides, which resulted in incomplete functional annotations. Here, we conducted a comparative analysis of the domains of SOMPs by including these two octocorals and two aragonitic scleractinians (*A. millepora* and *S. pistillata*) [3, 4]. The interspecies comparison of the SOMPs from each species was performed using a locally installed NCBI BLAST tool (v.2.2.25+).

## Results

### Genomic characteristics of *P. papillata* and *Chrysogorgia* sp.

Using a combination of PacBio long reads and Illumina short reads (Supplementary Fig. S2 and Supplementary Table S3), we generated high-quality genomes for *P. papillata* and *Chrysogorgia* sp. The genome sizes of *P. papillata* (618.13 Mb) and *Chrysogorgia* sp. (781.04 Mb) closely agreed with the *k*-mer-based estimates of 596.50 Mb and 774.93 Mb, respectively (Supplementary Fig. S3 and Supplementary Table S4). The contig N50 of the *P. papillata* assembly is 2.67 Mb and the *Chrysogorgia* sp. assembly is 2.61 Mb (Supplementary Table S4). The integrity of genome assembly was evaluated by back-mapping one library of paired-end data for each coral to its respective assembly. The analysis revealed that 99.34% (*P. papillata*) and 99.40% (*Chrysogorgia* sp.) of the Illumina paired-end reads aligned to the assembled genomes (Supplementary Table S5). BUSCO analysis with the metazoan database showed that the genome assemblies of *P. papillata* and *Chrysogorgia* sp. contained 91.61% and 88.36% complete BUSCO genes, respectively. Moreover, the proportion of duplicated BUSCO genes in both genomes was 1.89%, which was comparable to that in previously published octocoral genomes (Supplementary Table S6).

The genomes of *P. papillata* and *Chrysogorgia* sp. have a relatively high number of protein-coding genes as compared to the genomes of other anthozoans. Through the integration of multiple methodologies, 41,723 and 52,329 protein-coding genes (PCGs) were predicted in the genomes of *P. papillata* and *Chrysogorgia* sp., respectively (Supplementary Table S7). Among these PCGs, 37,974 (91.01%) in *P. papillata* and 47,126 (90.06%) in *Chrysogorgia* sp. were assigned functional annotations by comparing with public databases, including SwissProt, Pfam, NR, TrEMBL, eggNOG, KOG, KEGG, and GO (Supplementary Table S8). While considerable variation exists in the number of PCGs among different corals, octocoral genomes typically demonstrate higher counts than hexacoral genomes (Supplementary Table S9). The BUSCO completeness of the predicted genes in each genome was 94.23% for *P. papillata* and 92.87% for *Chrysogorgia* sp. (Supplementary Table S10), indicating that our predicted genes are highly complete. TEs influence genome evolution by modifying genomic architecture and affecting gene expression regulation. Using a combination of homology-based and *de novo* approaches, 294.04 Mb and 374.71 Mb (47.58% and 47.98%) of the *P. papillata* and *Chrysogorgia* sp. genomes (respectively) were identified as TEs (Fig. 1c and Supplementary Tables S11 and S12), with 23.76% and 23.84% of these TEs being class II DNA transposons and 23.81% and 24.14% of these TEs being class I retrotransposons (long interspersed nuclear elements [LINEs], long terminal repeats [LTRs], and short interspersed nuclear elements [SINEs]). Additionally, Kimura distance-based copy divergence analysis revealed similar expansion patterns for TEs across different coral lineages, except for *Trachythela* sp. (Tsp), with high compositional similarity (Fig. 1c and Supplementary Fig. S4).

### Phylogenomic analysis and gene-family evolution

The results of phylogenetic analysis clearly showed an Ediacaran origin for Anthozoa and the reciprocal monophyly of the subclasses Octocorallia and Hexacorallia (Fig. 1D). The 5 octocorals examined belonged to 2 newly established orders Scleralcyonacea (*P. papillata* [Ppap] and *Chrysogorgia* sp. [Csp]) and Malacalcyonacea (*Paramuricea clavata* [Pcla], Tsp, and *Dendronephthya gigantea* [Dgig]). Ppap and Csp formed a sister group, and their divergence was estimated at approximately the Triassic–Jurassic boundary (181 Ma), which corresponds to the transition period from aragonitic to calcite seas. The remaining 3 octocorals, i.e., Dgig, Pcla, and Tsp, originated in calcite seas during the Jurassic to Cretaceous periods. Additionally, the results supported the monophylies of Actiniaria (true sea anemones), Corallimorpharia (naked corals, mushroom anemones), and Scleractinia (stony corals) within Hexacorallia. The divergence between Scleractinia and Corallimorpharia (Amplexidiscus fenestrafer [Afen] and *Discosoma* sp. [Dsp]) occurred at 281 Ma (95% confidence interval: 349–221 Ma). Stony corals evolved the capacity to deposit aragonitic crystals in typical aragonitic seas during the Late Carboniferous to Triassic periods (281–214 Ma). Subsequently, stony corals diversified into two crown clades (“robust” and “complex”) in aragonitic seas during the mid-Triassic period (228–193 Ma).

Comparative analyses among a selection of the available anthozoan genomes showed that 286 gene families were expanded in *P. papillata* and 444 in *Chrysogorgia* sp., with 162 and 285 gene families showing significant expansion, respectively (Viterbi *P-*value < 0.05) (Fig. 1d and Supplementary Tables S13 and S14). The GO enrichment analysis of the expanded gene families identified 24 overrepresented GO categories in both *P. papillata* and *Chrysogorgia* sp. genomes (Supplementary Fig. S5). The significantly expanded gene families were involved in multiple processes: the phosphatidylinositol signaling pathway (PIP5 kinase activity and G-protein-coupled neurotransmitter receptor), cell–cell adhesion (cadherin binding and actinin binding), ion-transport processes (potassium channel regulator, vacuolar transport, and endosomal transport), and immune-related pathways (e.g., scavenger receptor activity, immunoglobulin production, and T-cell-receptor signaling pathway); this finding suggests their contributions to both biomineralization and immune responses.

### Skeletal structure characterization and biomineralized protein toolkit

**Figure 2:**
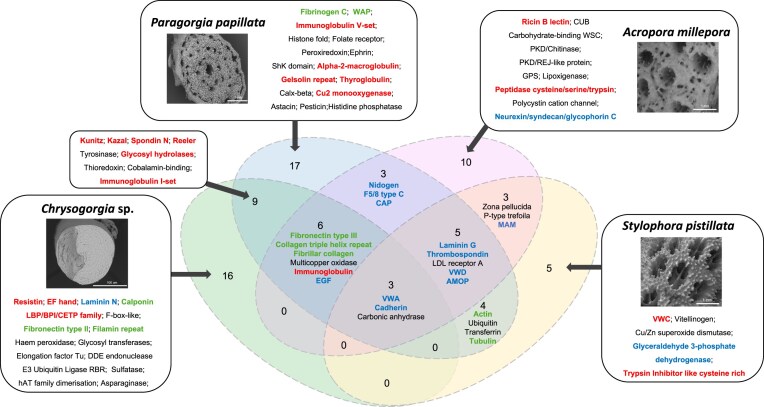
Venn diagram of the protein domains identified from the four coral SOMPs. SEM images illustrate the skeletal morphology of these four corals. Domains shown in bright green are related to the structural support of the skeleton. Domains shown in blue are mainly involved in cell adhesion. Immunity-related domains are represented in red.

To determine the types of the octocoral skeletons, we utilized Raman spectroscopy and SEM to analyze the skeletal structures. We found calcified sclerites embedded at both polyps and coenosarc tissues in *P. papillata* and *Chrysogorgia* sp. The axial skeleton of *P. papillata* showed the accumulation of high-magnesium calcite (HMC) sclerites with diverse morphologies in the form of a ring of regularly arranged central pores. In contrast, the axial skeleton of *Chrysogorgia* sp. exhibited a completely calcified HMC structure with a growth pattern resembling that of annual rings (Fig. 2 and Supplementary Fig. S6).

We further investigated the molecular basis of skeletal formation in octocorals and identified 64 and 37 SOMPs in the skeletal organic matrix space of *P. papillata* and *Chrysogorgia* sp., respectively (Supplementary Tables S15 and S16) by using LC-MS/MS protein sequencing and reference genome search. These SOMPs were validated by multiple unique peptides (Supplementary Tables S17 and S18). To characterize the conserved biomineralization toolkit, we performed a comparative skeleton proteomics analysis of the two calcite-forming octocorals and the two aragonitic scleractinians, *A. millepora* and *S. pistillata*. To detect multiple domains in the same protein from different evolutionary sources, we performed domain prediction and further compared the SOMPs in their functional context. Despite substantial differences in the skeletal morphology and microstructures of corals, we identified the following 3 functional domains common to the coral skeletal organic matrix space across all 4 species (Fig. 2): cadherin, von Willebrand factor type A (VWA), and carbonic anhydrase (CA). The cadherin domain, which contains conserved cysteine residues and calcium-binding motifs, is involved in intercellular adhesion, and this domain was exclusively detected in protocadherin-like or classical cadherin (Supplementary Tables S15 and S16), which belongs to the cadherin superfamily. The VWA domain was identified in protocadherin, collagen, and fibrillin-2 (Supplementary Tables S15 and S16). CA occurred in a superfamily of predominantly zinc-binding metalloenzymes that catalyze the interconversion of CO_2_ into HCO_3_^−^, all of which contain predicted signal peptides or transmembrane domains (Supplementary Tables S15 and S16).

### Function and composition of SOMPs

The SOMPs embedded within octocoral skeletons can be classified into 5 main categories according to their domain function prediction, namely cell adhesion, structure support, immune regulation, enzymes, and other functional proteins (Fig. 2 and Supplementary Tables S15 and S16). The composition of SOMPs largely varied among corals with different skeletal types, and each coral retained distinct functional domains, with the proportion of unique functional domains of up to 47% in *P. papillata* (Fig. 2). Compared to stony corals, we found that octocorals possess numerous proteins containing immunity-associated domains, including alpha-2-macroglobulin, spondin 2, agrin, and putative defense protein 3 (Fig. 2 and Supplementary Tables S15 and S16). The presence of these proteins is consistent with the expansion of gene families related to immune regulation (Supplementary Fig. S5), suggesting the presence of immune regulatory pathways within the coral skeletal organic matrix that enhance defense mechanisms during skeletal formation and prevent pathogen invasion.

**Figure 3:**
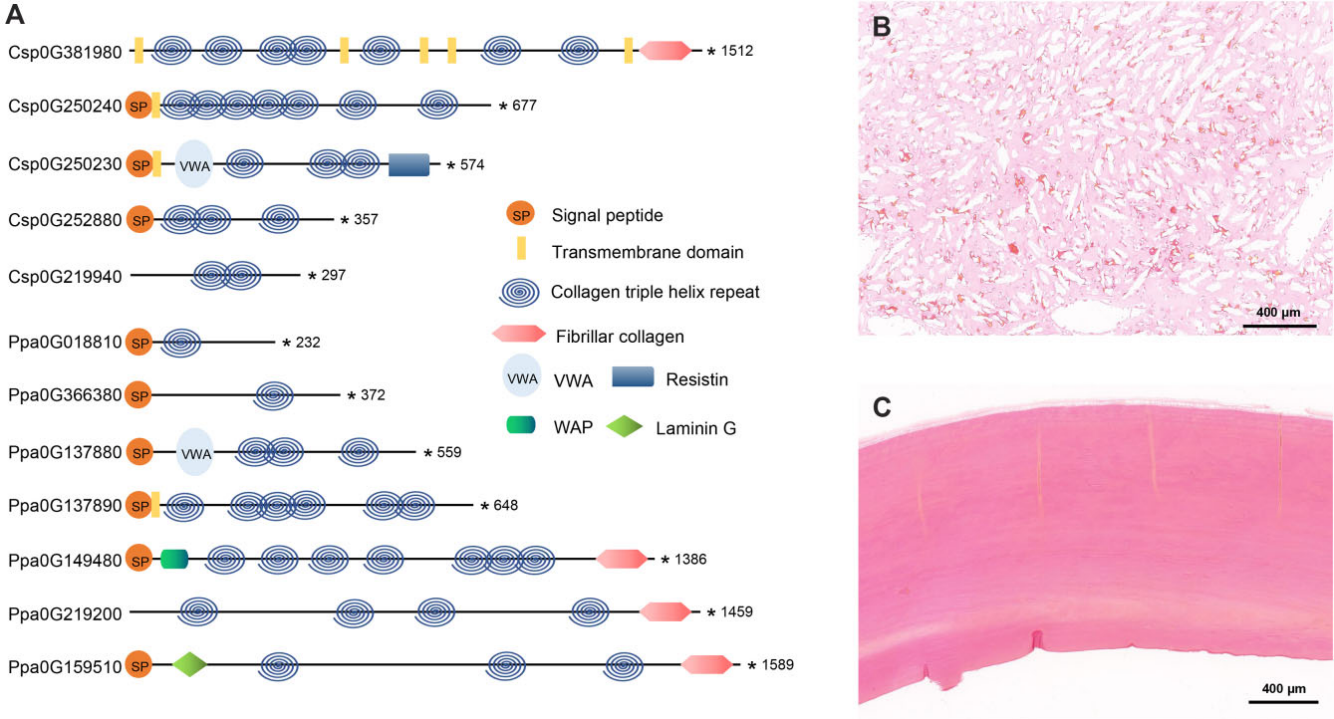
Structural domain and distribution of collagen in the axial skeleton of *P. papillata* and *Chrysogorgia* sp. (a) Schematic representation of 5 and 7 collagen proteins identified in the proteomes of *P. papillata* and *Chrysogorgia* sp., respectively. (b,c) VG staining results of axial skeletons of *P. papillata* (b) and *Chrysogorgia* sp. (c). The axial skeleton areas containing collagen fibers appear dark red. In *P. papillata*, the wart-like branching structures are sclerites with distributed collagen fibers.

We identified 7 and 5 collagen types in the skeletons of *P. papillata* and *Chrysogorgia* sp., respectively, and the helical regions of all collagens exhibited the characteristic Gly-X-Y periodic repeats (Fig. 3a and Supplementary Tables S15 and S16). Pfam domain analysis revealed that some collagens have undergone recombination with VWA, WAP, and laminin G domains, potentially contributing to the diverse functions of collagens. To observe the distribution of collagen fibers in tissues, VG staining was performed on the axial skeleton of *P. papillata* and *Chrysogorgia* sp. The results showed that collagen fibers in *P. papillata* were mainly distributed on the calcified sclerites along its unconsolidated and unfused scleritic axis; in contrast, collagen fibers in *Chrysogorgia* sp. appeared deep red throughout the axial skeleton, indicating their widespread distribution in the mineralized skeleton (Fig. 3b,c and Supplementary Fig. S7).

## Discussion

This study presents the sequenced and assembled draft genomes and skeletal proteomes of 2 octocoral species, *P. papillata* and *Chrysogorgia* sp., contributing to the growing collection of octocoral genomes and enhancing our understanding of skeletal formation and evolutionary history in octocorals. We found that, except for *P. clavata*, the genome sizes of the 2 octocoral species, *P. papillata* and *Chrysogorgia* sp., were considerably larger than those of the published genomes of octocorals and hexacorals; this could be potentially attributed to the large number of repetitive sequences in the genomes of *P. papillata* and *Chrysogorgia* sp. Phylogenetic analyses revealed that the 5 calcite-forming octocorals examined belonged to 2 newly established orders, namely Scleralcyonacea and Malacalcyonacea [33], all of which emerged in calcite seas during the Jurassic to Cretaceous periods. The origin of corals with different CaCO_3_ polymorphs is generally considered to be related to the palaeoclimate ocean conditions [34], suggesting that elevated Mg/Ca ratios in calcite seas may have favored calcite skeleton formation in octocorals [35].

The skeletal structure of corals contains an embedded organic matrix with a specific set of proteins that can stabilize amorphous calcium carbonate and regulate the nucleation, orientation, and polymorph selection [9, 36]. Understanding the composition of SOMPs in the coral skeleton is essential for elucidating the ancient mechanisms underlying coral skeleton formation and evolution. By comparing the proteomes of different coral skeletons, this study revealed a conserved protein toolkit utilized by both calcite-forming octocorals and aragonitic stony corals for biomineralization. Despite variations in skeletal morphology and polymorphs, the biomineralization toolkit composed of cadherin, VWA, and CA is evolutionarily conserved and represents a fundamental component of the biomineralization process for skeletal construction. The cadherin domain, a Ca^2+^-dependent transmembrane glycoprotein present in both protocadherin and classical cadherin, belongs to extracellular matrix-like proteins. A potential role of cadherin in coral skeleton formation is mediating connections between calicoblastic cells and the organic matrix within the skeleton [4, 5]. The VWA domain is predominantly associated with proteins involved in cell adhesion and structural support. This domain interacts with chitin or fibronectin to form a cross-linked organic matrix network, thereby guiding skeletal growth and morphological differentiation [11, 37]. CA is a key enzyme involved in a wide range of physiological functions and is present in all metazoan clades [38, 39]. CA proteins identified in the skeletal proteomes of octocorals and stony corals possess transmembrane domains or signaling peptides, suggesting their role as secreted or membrane-associated CAs that catalyze the interconversion of CO_2_ to HCO_3_^−^ in the extracellular calcifying medium (ECM) and provide inorganic carbon for CaCO_3_ precipitation.

Skeletal proteome analysis and collagen fiber staining revealed substantial collagen presence in the octocoral skeletons. Abundant collagen-like proteins have also been detected in the precious red coral *Corallium rubrum* and a gorgonian coral with a calcite skeleton [40, 41]. The presence of abundant collagen in the skeletal organic matrix space appears to be a distinctive characteristic of calcite-forming octocorals. Previous studies have indicated that, during the skeleton formation process, the initial collagen triple-helix structure contains negatively charged carboxyl groups on its exterior; these groups bind with calcium ions to form mineralized collagen fibers that provide a template for mineral deposition and promote CaCO_3_ nucleation [42, 43]. This finding suggests that collagen may serve as the fundamental structural framework of octocoral skeletons. We also observed frequent recombination of collagen domains in the axial skeletons of octocorals, including binding to VWA, laminin G, and WAP domains. The binding of new domains may confer novel genetic functions to collagen during the deposition of CaCO_3_ skeletal structures. The VWA and laminin G domains, typically found in ECM proteins, are involved in cell–substrate adhesion and the arrangement of CaCO_3_ crystals [36, 44]. The presence of these proteins may facilitate the cross-linking of collagen with other non-collagenous proteins to establish the core matrix framework. The WAP domain plays a pivotal role in regulating innate immunity, protecting against microbial invasion, and promoting mucosal tissue repair [45]. A previous study demonstrated that proteins involved in innate immune responses can assist stony corals in resisting skeletal pathogen infiltration, thereby enhancing their calcification capacity [46]. Based on these findings, we propose that the binding of the collagen domain to the WAP domain enhances immunity in a matrix framework environment, thereby promoting the deposition of calcified skeleton in octocorals. Octocorals are predominantly passive suspension feeders, and their colonies frequently adopt a clumped, tree-like, or net-like structure oriented toward ocean currents [47]. In this context, collagen might play a critical role in strengthening skeletal structure and enhancing skeletal flexibility to withstand ocean current conditions.

## Data Description

This study presents the assembly, annotation, and comparative genomic analyses of genomes of two octocoral species: *P. papillata* and *Chrysogorgia* sp. We also characterized the axial skeletal proteomes of these two octocorals to identify a fundamental toolkit for coral calcification by comparison with the skeletal proteomes of aragonitic corals. We found that collagen in the axial skeleton of octocorals has evolved structural domains linked to matrix adhesion and immunity, which may confer novel genetic functions for calcification in octocorals. The data obtained from the genomes and proteomes expand the existing octocoral genome database and provide significant insights into the molecular mechanisms and evolutionary history of coral skeletal formation.

## Supplementary Material

giaf031_Supplemental_Files

giaf031_GIGA-D-24-00546_Original_Submission

giaf031_GIGA-D-24-00546_Revision_1

giaf031_Response_to_Reviewer_Comments_Original_Submission

giaf031_Reviewer_1_Report_Original_Submission Eldon Ball -- 12/16/2024

giaf031_Reviewer_2_Report_Original_Submission Timothy Gordon Stephens, Ph.D. -- 1/28/2025

## Data Availability

The genomes of the two deep-sea octocoral species investigated in this study have been deposited in the NCBI database under BioProject numbers PRJNA999483 (*P. papillata*) and PRJNA999484 (*Chrysogorgia* sp.). The whole-genome sequencing data and RNA-seq data were deposited in the sequence read archive database under accession numbers SRR25705840-SRR25705842 (*P. papillata*) and SRR25705989-SRR25705991 (*Chrysogorgia* sp.). The genome-related annotation files are accessible through Figshare [50]. The raw data of proteomic sequencing are available in the ProteomeXchange database under project ID IPX0010006000. The specific accessions are provided in the respective Material and Methods sections describing the data and analyses. All additional supporting data are available in the *GigaScience* repository, GigaDB [51–53].

## Additional Files

**Supplementary Figure S1:** A schematic representation of the main components and ion transport involved in biomineralization at the calcified site of coral. Calcification process is precisely controlled and occurs in the extracellular calcifying medium (ECM) lined by the calicoblastic ectoderm that initiate and control the precipitation reaction. The entry of calcium ions into the ECM can occur either actively through the transmembrane pathway via transporters (namely Ca^2+^-ATPase or Na^+^/Ca^2+^ exchanger) or, less commonly, passively through the paracellular pathway. Carbonic anhydrase (CA) facilitates/catalyzes the interconversion of CO_2_ into HCO_3_^−^. These HCO_3_^−^ are transported via the bicarbonate transporters to the ECM, where they react with Ca^2+^ to form CaCO_3_ and ultimately the coral skeleton. The skeletal organic matrix proteins or other organic molecules can promote the formation of macroscopic structures in crystals (see Fig. 2 of 48, with minor modifications).

**Supplementary Figure S2:** Statistics of reads length distribution: 66.1% and 82.2% of clean reads in *P. papillata* and *Chrysogorgia* sp. are larger than 18 kb, respectively.

**Supplementary Figure S3:** The *k*-mer distribution of *P. papillata* (a) and *Chrysogorgia* sp. (b). The result from jellyfish v.2.2.6 (49) with a *k*-mer size of 17. The estimated heterozygosity is 1.13% and 1.44% for *P. papillata* and *Chrysogorgia* sp., respectively, with estimated genome sizes of 595.50 Mb for *P. papillata* and 774.93 Mb for *Chrysogorgia* sp.

**Supplementary Figure S4:** Distribution of the divergence rate of each type of repetitive. The historical transposable element (TE) divergence was compared in the following species: *Paragorgia papillata* (Ppap), *Chrysogorgia* sp. (Csp), *Trachythela* sp. (Tsp), *Dendronephthya gigantea* (Dgig), *Paramuricea clavata* (Pcla) and *Acropora digitifera* (Adig). The analysis was conducted using the Kimura distance-based copy divergence method.

**Supplementary Figure S5:** Enriched Gene Ontology (GO) terms of the expanded gene families in *P. papillata* (a) and *Chrysogorgia* sp. (b). The blue and red bars indicate the p/FDR value of genes in the expanded gene families, respectively. The heatmap was plotted using an online platform for data analysis and visualization (https://www.bioinformatics.com.cn, accessed 20 February 2023).

**Supplementary Figure S6:** Axial skeletal traits of *P. papillata* and *Chrysogorgia* sp. (a,c) represent cross-sections of the axial skeleton of *P. papillata* and *Chrysogorgia* sp., respectively. The Raman spectra demonstrate that the CaCO_3_ polymorphs of *P. papillata* and *Chrysogorgia* sp. are calcite, and the characteristic peaks are 2,283.59, 714.17, and 1,088.14 cm^−1^ (b) and 284.75, 714.49, and 1,088.09 cm^−1^ (d), respectively.

**Supplementary Figure S7:** The results of van Gieson staining of the axial skeleton of *P. papillata* (a,b) and *Chrysogorgia* sp. (c,d). (a,c) Cross-sections of the axial skeletons of *P. papillata* and *Chrysogorgia* sp., respectively. (b,d) Longitudinal sections of axial skeletons of *P. papillata* and *Chrysogorgia* sp., respectively. The axial skeleton of *Chrysogorgia* sp. is brittle after decalcification, resulting in fracture during the slicing process. However, the presence of collagen fibers can still be observed throughout the entire axial skeleton tissue.

**Supplementary Table S1:** Summary of species for comparative genomes.

**Supplementary Table S2:** Fossil constraints used in the MCMCtree analyses in this study.

**Supplementary Table S3:** Sequence information used in this study.

**Supplementary Table S4:** Genome assembly statistics for *P. papillata* and *Chrysogorgia* sp.

**Supplementary Table S5:** The mapping rate of sequencing reads to assembled genomes.

**Supplementary Table S6:** The completeness of assembled genome by BUSCO assessment.

**Supplementary Table S7:** Gene prediction through integrating multiple methods.

**Supplementary Table S8:** Functional annotation of *P. papillata* and *Chrysogorgia* sp. predicted gene.

**Supplementary Table S9:** Data on annotated genes from the *P. papillata, Chrysogorgia* sp. and other anthozoan used in this study.

**Supplementary Table S10:** The completeness of *P. papillata* and *Chrysogorgia* sp. gene by BUSCO assessment.

**Supplementary Table S11:** TE constitution in *P. papillata* and *Chrysogorgia* sp. genome.

**Supplementary Table S12:** Summary statistics of repetitive sequences.

**Supplementary Table S13:** The annotation of expansion/contraction gene families in *Paragorgia papillata*.

**Supplementary Table S14:** The annotation of expansion/contraction gene families in *Chrysogorgia* sp.

**Supplementary Table S15:** List of skeletal organic matrix proteins in *P. papillata*.

**Supplementary Table S16:** List of skeletal organic matrix proteins in *Chrysogorgia* sp.

**Supplementary Table S17:** Unique peptide list identified by LC-MS/MS analysis in *P. papillata*.

**Supplementary Table S18:** Unique peptide list identified by LC-MS/MS analysis in *Chrysogorgia sp*.

## Abbreviations

Stony coral: Scleractinia; Octocoral: Octocorallia; CaCO_3_: calcium carbonate; SOMPs: skeletal organic matrix proteins; TE: transposable element; OG: ortholog groups; VG: van Gieson staining; SEM: scanning electron microscope; CA: carbonic anhydrase; SDS-PAGE: sodium dodecyl sulfate–polyacrylamide gel electrophoresis; ASM: acid-soluble matrix; AIM: acid-insoluble matrix; BUSCO: benchmarking universal single-copy orthologs; LINE: long interspersed nuclear element; LTR: long terminal repeats; SINE: short interspersed nuclear element; HMC: high-magnesium calcite; VWA: von Willebrand factor type A; WAP: WAP-type (whey acidic protein) “four-disulfide core”; ECM: extracellular calcifying medium.
