# Supplementary material for: The molecular basis of octocoral calcification revealed by genome and skeletal proteome analyses
Source: Gigascience. 2025 Apr 1;14:giaf031. doi: 10.1093/gigascience/giaf031 (PMC11959691; doi:10.1093/gigascience/giaf031)
Supplement: giaf031_Supplemental_Files [file giaf031_supplemental_files.zip › Supplementary Figure.docx]

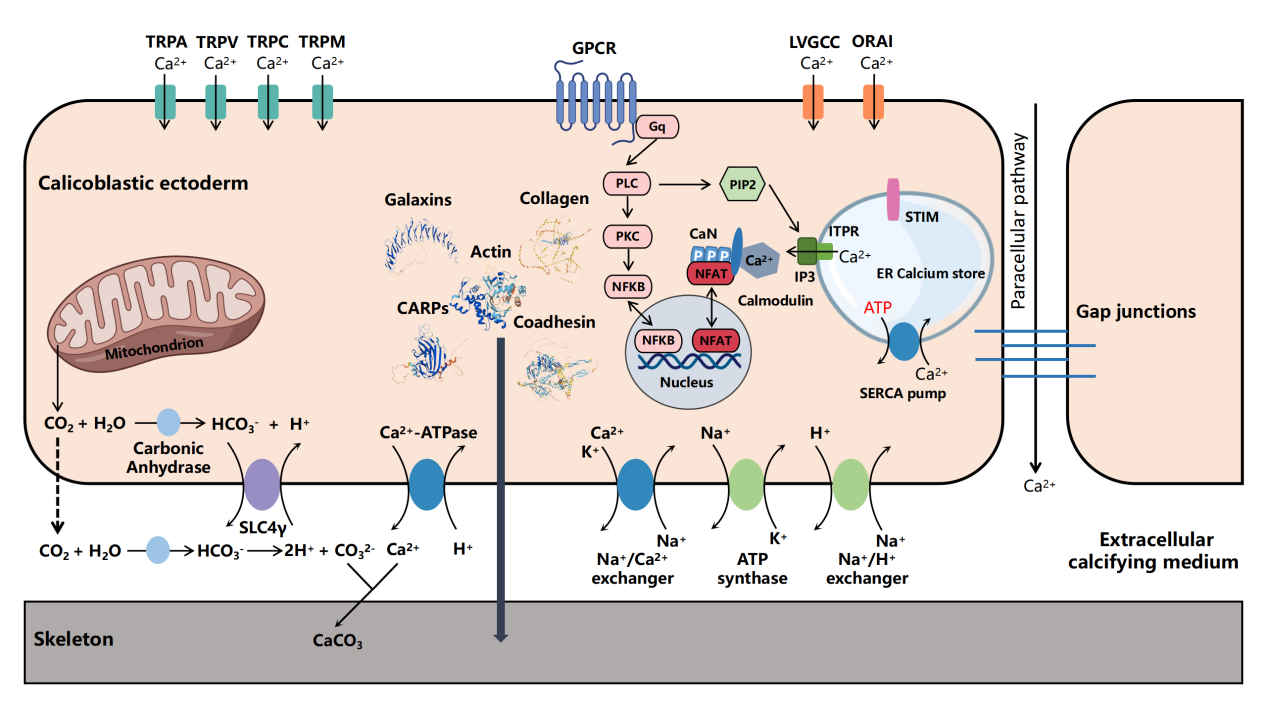


**Fig. S1. A schematic representation of the main components and ion transport involved in biomineralization at the calcified site of coral.** Calcification process is precisely controlled and occurs in the extracellular calcifying medium (ECM) lined by the calicoblastic ectoderm that initiate and control the precipitation reaction. The entry of calcium ions into the ECM can occur either actively through the transmembrane pathway via transporters (namely Ca^2+^-ATPase or Na^+^/Ca^2+^ exchanger) or less commonly, passively through the paracellular pathway. Carbonic anhydrase (CA) facilitates/catalyzes the interconversion of CO_2_ into HCO_3_^-^. These HCO_3_^-^ are transported via the bicarbonate transporters to the ECM, where they react with Ca^2+^ to form CaCO_3_ and ultimately the coral skeleton. The skeletal organic matrix proteins or other organic molecules can promote the formation of macroscopic structures in crystals (see Figure 2 of Bhattacharya et al. (2016), with minor modifications).


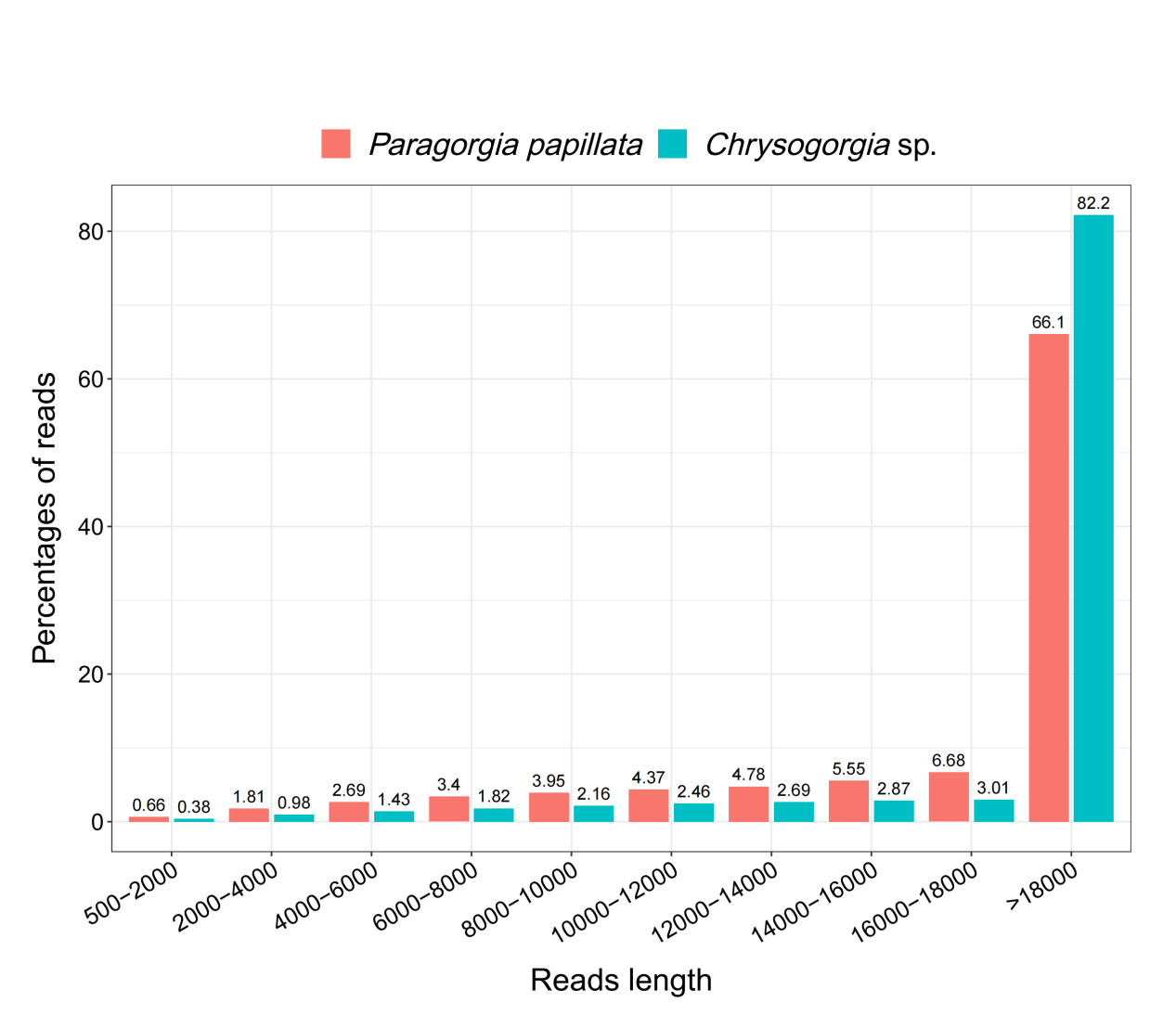


**Fig. S2. Statistics of Reads length distribution.** 66.1% and 82.2% of clean reads in *P. papillata* and *Chrysogorgia* sp. are larger than 18 kb, respectively.


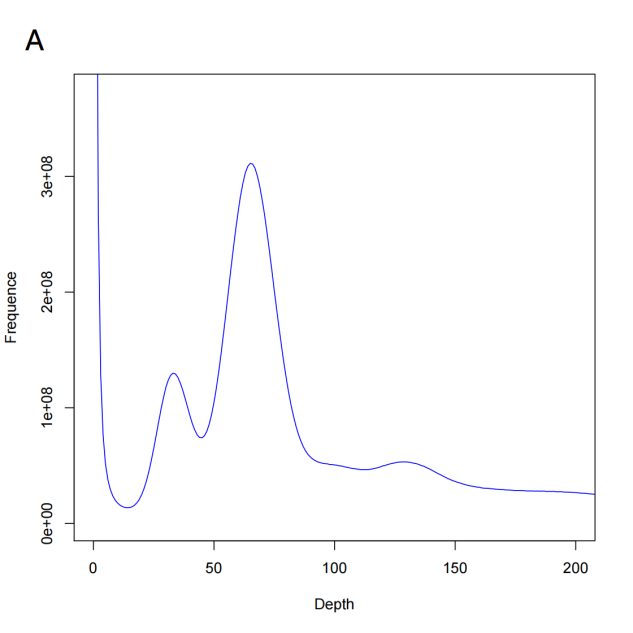

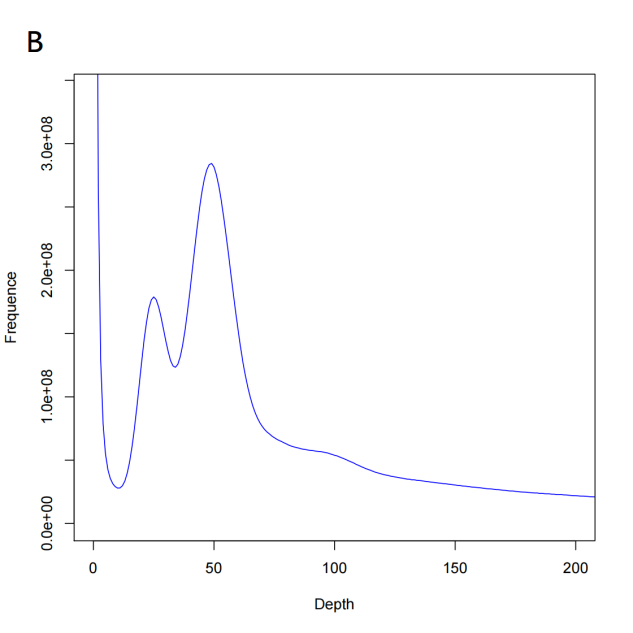


**Fig. S3. The *k*-mer distribution of** ***P. papillata* (A) and *Chrysogorgia* sp. (B).** The result from jellyfish v2.2.6 (Marçais and Kingsford 2011) with a kmer size of 17. The estimated heterozygosity is 1.13% and 1.44% for *P. papillata* and *Chrysogorgia* sp., respectively, with the estimated genome size 595.50 Mb for *P. papillata* and 774.93 Mb for *Chrysogorgia* sp.

**
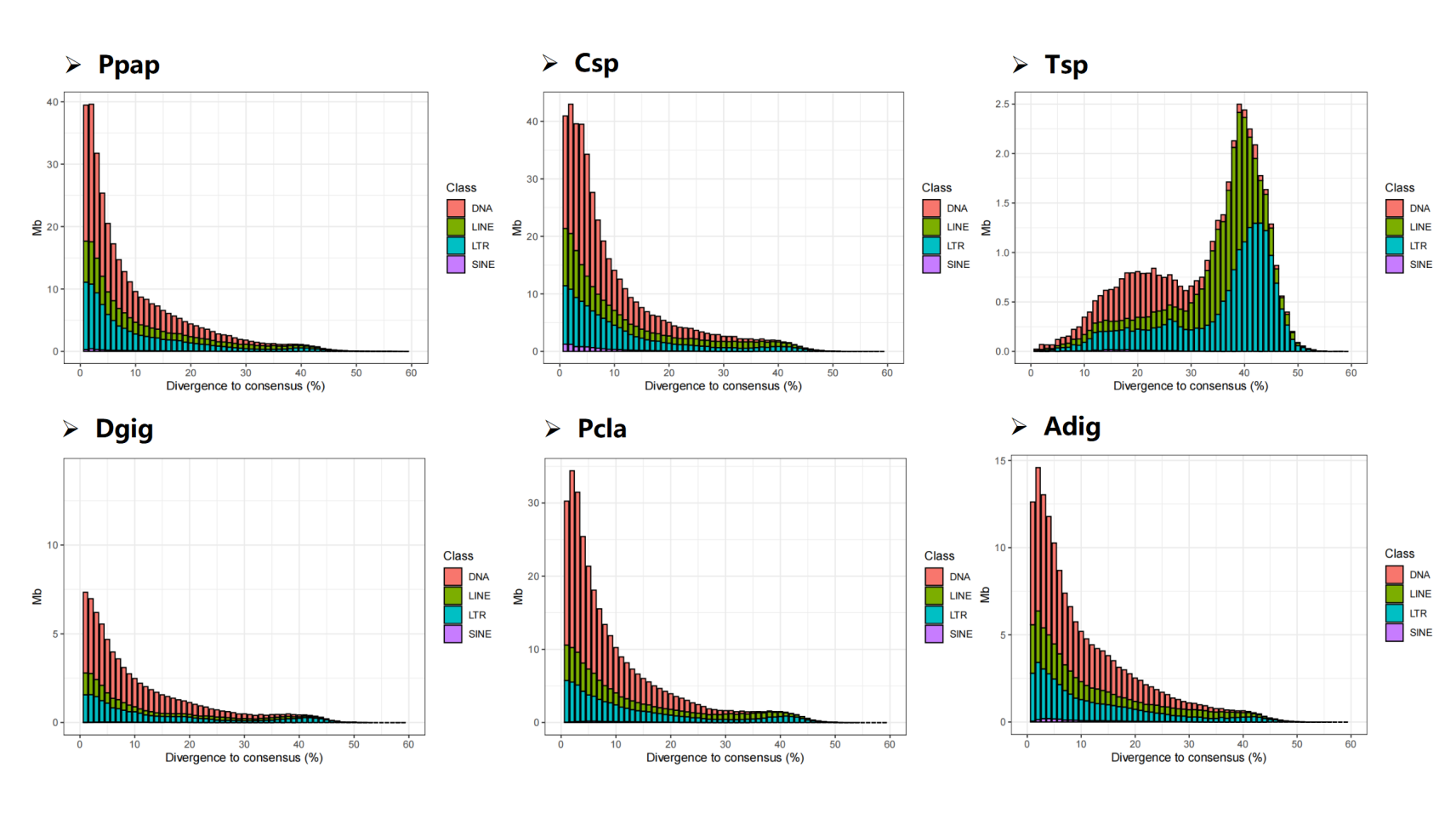
**

**Fig. S4. Distribution of the divergence rate of each type of repetitive.** The historical transposable element (TE) divergence was compared in the following species: *Paragorgia papillata* (Ppap), *Chrysogorgia* sp. (Csp), *Trachythela* sp. (Tsp), *Dendronephthya gigantea* (Dgig), *Paramuricea clavata* (Pcla) and *Acropora digitifera* (Adig). The analysis was conducted using the Kimura distance-based copy divergence method.


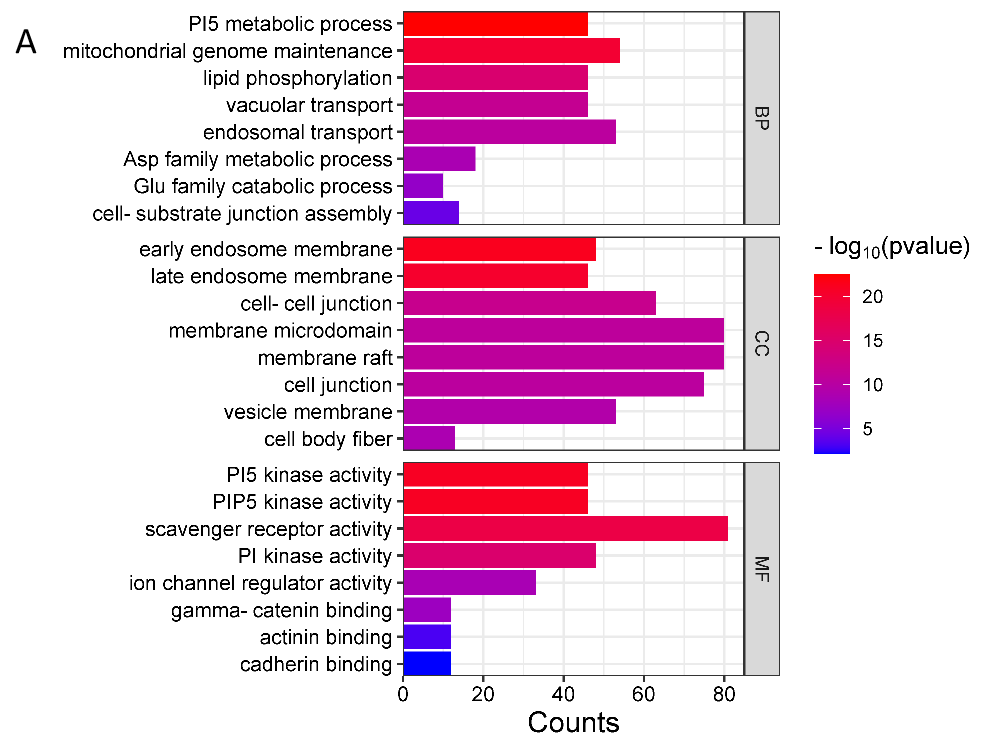


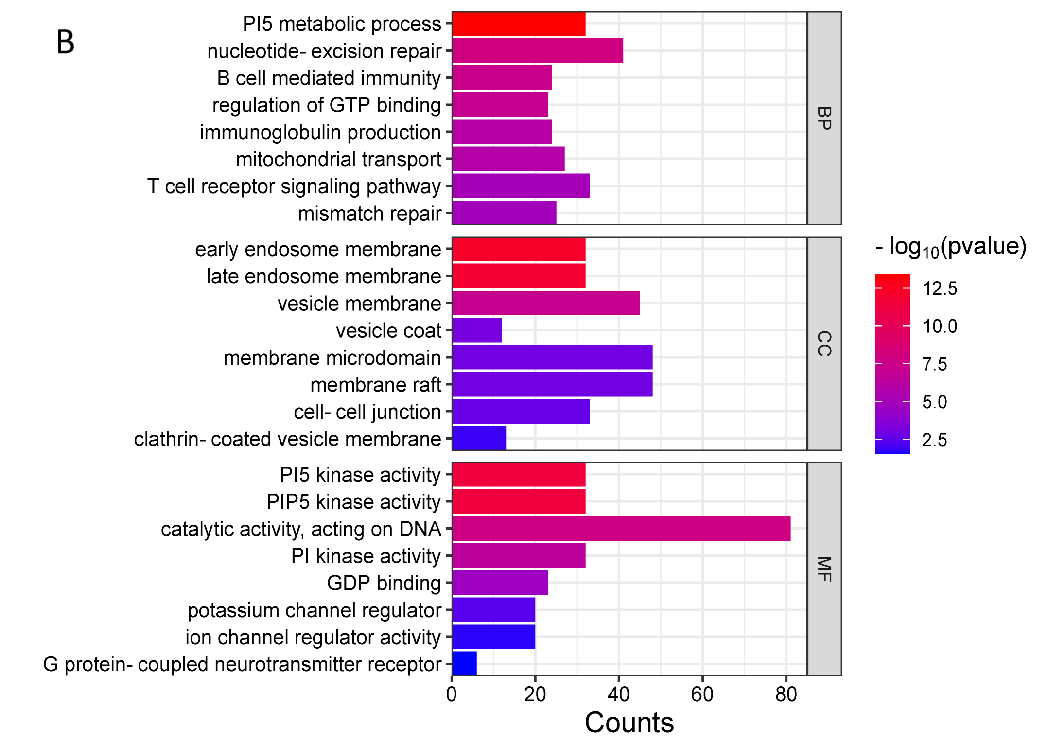


**Fig. S5. Enriched Gene Ontology (GO) terms of the expanded gene families in *P. papillata* (A) and *Chrysogorgia* sp. (B).** The blue and red bars indicate the p/FDR value of genes in the expanded gene families, respectively. The heatmap was plotted using an online platform for data analysis and visualization (https://www.bioinformatics.com.cn, last accessed on 20 Feb 2023).

^
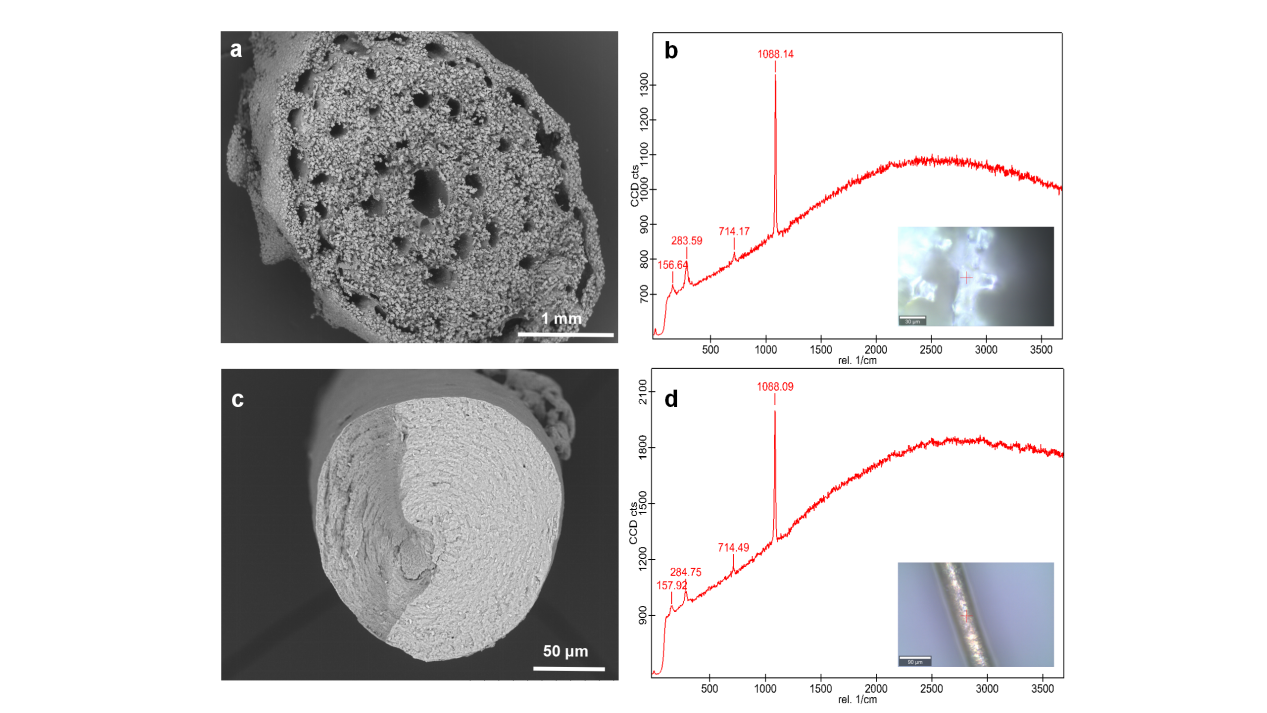
^

**Fig. S6.** **Axial skeletal traits of** ***P. papillata* and** ***Chrysogorgia* sp.** (a) and (c) represent cross-sections of the axial skeleton of *P. papillata* and *Chrysogorgia* sp., respectively. The Raman spectra demonstrate that the CaCO_3_ polymorphs of *P. papillata* and *Chrysogorgia* sp. are calcite, and the characteristic peaks are 2283.59, 714.17, and 1088.14 cm^-1^ (b) and 284.75, 714.49, and 1088.09 cm^-1^ (d), respectively.


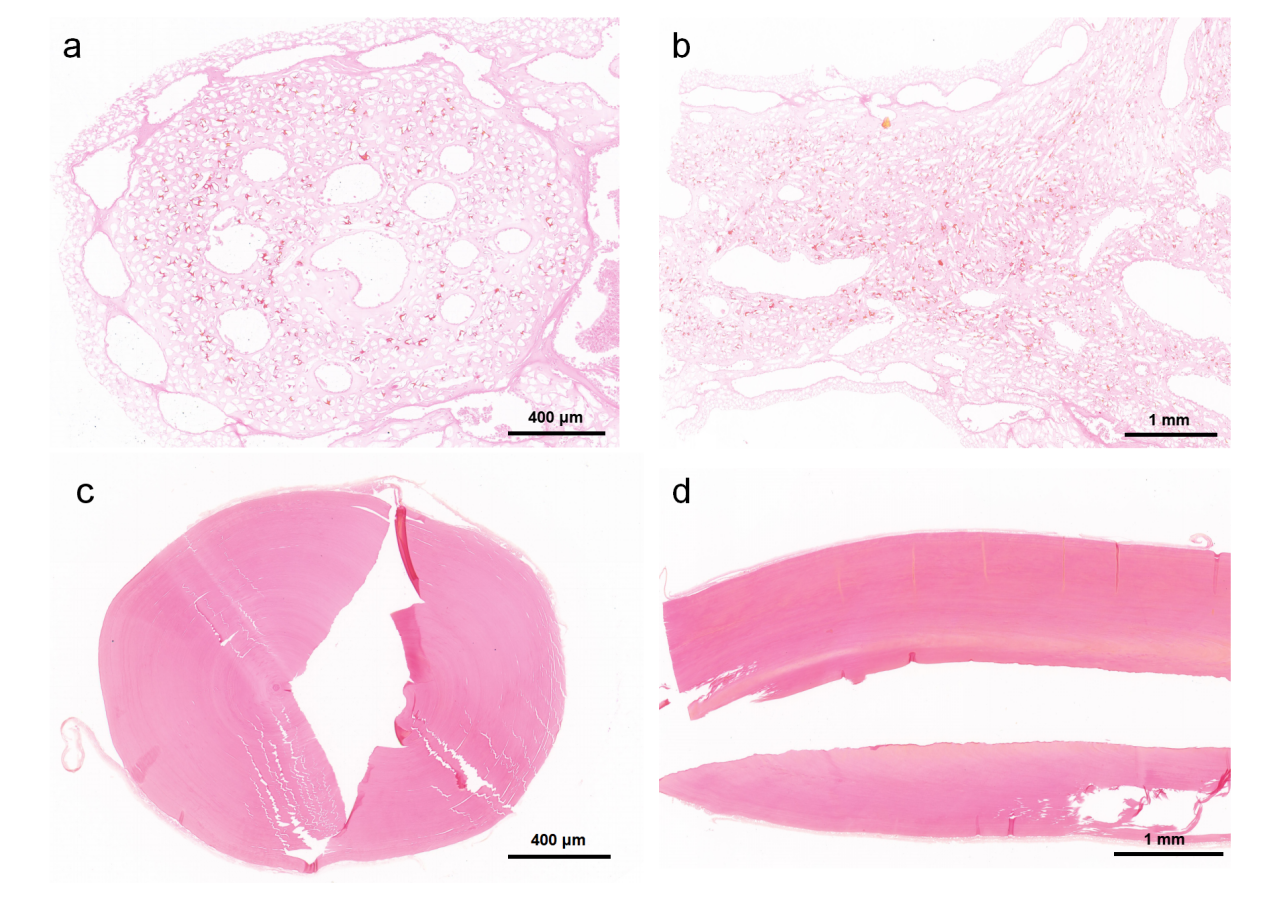


**Fig. S7. The results of van gieson staining of the axial skeleton of *P. papillata* (a and b) and *Chrysogorgia* sp. (c and d).** (a) and (c) represent cross-sections of the axial skeletons of *P. papillata* and *Chrysogorgia* sp., respectively; (b) and (d) represent longitudinal sections of axial skeletons of *P. papillata* and *Chrysogorgia* sp. The axial skeleton of *Chrysogorgia* sp. is brittle after decalcification, resulting in fracture during the slicing process. However, the presence of collagen fibers can still be observed throughout the entire axial skeleton tissue.

**References:**

Bhattacharya D, Agrawal S, Aranda M, Baumgarten S, Belcaid M, Drake JL, et al. Comparative genomics explains the evolutionary success of reef-forming corals. eLife, 2016;5:1–26.

Marçais G, Kingsford C. A fast, lock-free approach for efficient parallel counting of occurrences of k-mers. Bioinformatics, 2011;27:764–70.
