# Supplementary material for: The molecular basis of octocoral calcification revealed by genome and skeletal proteome analyses
Source: Gigascience. 2025 Apr 1;14:giaf031. doi: 10.1093/gigascience/giaf031 (PMC11959691; doi:10.1093/gigascience/giaf031)
Supplement: giaf031_Supplemental_Files [file giaf031_supplemental_files.zip › Supplementary Table.docx]

**Table S1.** Summary of species for comparative genomes.

| Species | Abbreviation | Reference | Link |
| --- | --- | --- | --- |
| *Paragorgia papillata* | Ppap | This study | [https://doi.org/10.6084/m9.figshare.23984235](https://doi.org/10.6084/m9.figshare.23984235.) |
| *Chrysogorgia* sp. | Csp | This study | [https://doi.org/10.6084/m9.figshare.23984235](https://doi.org/10.6084/m9.figshare.23984235.) |
| *Dendronephthya gigantea* | Dgig | Jeon et al. 2019 | https://ftp.ncbi.nlm.nih.gov/genomes/all/GCA/004/324/835/GCA_004324835.1_DenGig_1.0/ |
| *Trachythela* sp. | Tsp | Zhou et al. 2021 | https://www.ncbi.nlm.nih.gov/bioproject/PRJNA661975/ |
| *Paramuricea clavata* | Pcla | Ledoux et al. 2020 | https://denovo.cnag.cat/pclavata_data?fid=778#block-likable-page-title |
| *Amplexidiscus fenestrafer* | Afen | Wang et al. 2017 | http://corallimorpharia.reefgenomics.org/download/ |
| *Discosoma sp.* | Dsp | Wang et al. 2017 | http://corallimorpharia.reefgenomics.org/download/ |
| *Actinoscyphia* sp. | Asp | Li et al. unpublished | https://figshare.com/articles/dataset/Signatures_of_photosensitivity_circadian_rhythm_and_advanced_neurons_revealed_by_a_deepsea_Venus_flytrap_sea_anemone_genome/19772119 |
| *Exaiptasia diaphana* | Edia | Baumgarten et al. 2015 | https://ftp.ncbi.nlm.nih.gov/genomes/all/GCA/001/417/965/GCA_001417965.1_Aiptasia_genome_1.1/ |
| *Paraphelliactis xishaensis* | Pxis | Feng et al. 2021 | https://figshare.com/articles/dataset/deep-sea_anemone_genome/13076387 |
| *Nematostella vectensis* | Nvec | Putnam et al. 2007 | https://ftp.ncbi.nlm.nih.gov/genomes/all/GCA/932/526/225/GCA_932526225.1_jaNemVect1.1/ |
| *Acropora digitifera* | Adig | Shinzato et al. 2020 | https://www.ncbi.nlm.nih.gov/datasets/genome/GCA_014634065.1/ |
| *Astreopora myriophthalma* | Amyr | Shinzato et al. 2020 | https://www.ncbi.nlm.nih.gov/datasets/genome/GCA_014634185.1/ |
| *[Montipora efflorescens](https://www.ncbi.nlm.nih.gov/genome/80926)* | Meff | Shinzato et al. 2020 | https://www.ncbi.nlm.nih.gov/datasets/genome/GCA_014634505.1/ |
| *[Acropora millepora](https://www.ncbi.nlm.nih.gov/genome/2652)* | Amil | Ying et al. 2019 | https://ftp.ncbi.nlm.nih.gov/genomes/all/GCA/013/753/865/GCA_013753865.1_Amil_v2.1/ |
| *[Stylophora pistillata](https://www.ncbi.nlm.nih.gov/genome/12040)* | Spis | Voolstra et al. 2017 | https://ftp.ncbi.nlm.nih.gov/genomes/all/GCA/002/571/385/GCA_002571385.1_Stylophora_pistillata_v1/ |
| *[Orbicella faveolata](https://www.ncbi.nlm.nih.gov/genome/13173)* | Ofav | Prada et al.2016 | https://ftp.ncbi.nlm.nih.gov/genomes/all/GCA/002/042/975/GCA_002042975.1_ofav_dov_v1/ |
| *Pocillopora damicornis* | Pdam | Cunning et al. 2018 | https://ftp.ncbi.nlm.nih.gov/genomes/all/GCA/003/704/095/GCA_003704095.1_ASM370409v1/ |
| *Pocillopora verrucosa* | Pver | Buitrago-López et al. 2020 | https://ftp.ncbi.nlm.nih.gov/genomes/all/GCA/014/529/365/GCA_014529365.1_Pver_genome_assembly_v1.0/ |
| *Hydra vulgatis* | Hvul | Chapman et al. 2010 | https://www.ncbi.nlm.nih.gov/datasets/genome/GCF_000004095.1/ |

**Table S2.** Fossil constraints used in the MCMCtree analyses in this study.

| Fossil constraints | upper limit (Ma) | lower limit (Ma) | References |
| --- | --- | --- | --- |
| Stylophora and Astreopora | 225.1 | 164.7 | Behrensmeyer and Turner, 2013 |
| Astreopora and Montipora | 164.7 | 136.4 | Shinzato et al. 2020 |
| Acropora and Montipora | 136.4 | 70.6 | Shinzato et al. 2020  Quattrini et al. 2020 |
| Acropora spp. | 76.4 | 55.8 | Quattrini et al. 2020 |
| Cnidaria | 741 | 520 | Quattrini et al. 2020 |

**Table S3.** Sequence information used in this study.

|  | *P. papillata* | *Chrysogorgia* sp. |
| --- | --- | --- |
| PacBio sequencing | | |
| Reads number | 7,179,442 | 5,120,497 |
| Reads base (bp) | 111,940,507,627 | 112,036,321,414 |
| Reads LenN50 (bp) | 22,650 | 32,951 |
| Reads LenMax (bp) | 282,232 | 294,353 |
| Reads LenMean (bp) | 15,592 | 21,880 |
| Genome coverage | 181.09 | 143.45 |
| Illumina sequencing | | |
| Raw paired reads | 169,635,159 | 163,700,654 |
| Raw Base (bp) | 50,890,547,700 | 49,110,196,200 |

**Table S4.** Genome assembly statistics for *P. papillata* and *Chrysogorgia* sp.

|  | *P. papillata* | *Chrysogorgia* sp. |
| --- | --- | --- |
|  | Contig | Contig |
| Number | 473 | 522 |
| Length (bp) | 618,132,567 | 781,039,190 |
| N50 (bp) | 2,672,309 | 2,612,635 |
| N90 (bp) | 698,750 | 719,945 |
| Maximum (bp) | 17,301,290 | 18,279,194 |
| GC content | 37.82% | 37.37% |

**Table S5.** The mapping rate of sequencing reads to assembled genomes.

|  | *P. papillata* | *Chrysogorgia* sp. |
| --- | --- | --- |
| Illumina reads mapping | | |
| Total reads | 290,172,450 | 282,848,010 |
| Mapped reads | 288,247,893 | 281,162,450 |
| Mapped (%) | 99.34 | 99.40 |
| Properly mapped reads | 271,784,754 | 257,755,630 |
| Properly mapped (%) | 93.66 | 91.13 |
| Pacbio read mapping | | |
| Total reads | 7,179,442 | 5,120,497 |
| Mapped reads | 6,616,715 | 4,851,200 |
| Mapped (%) | 92.16 | 94.74 |

**Table S6.** The completeness of assembled genome by BUSCO assessment.

|  | *Paragorgia papillata* | *Chrysogorgia* sp. | *Eunicella verrucosa* | *Dendronephthya gigantea* | *Trachythela* sp. | *Paramuricea*  *clavata* |
| --- | --- | --- | --- | --- | --- | --- |
| Complete BUSCOs(C) | 874 (91.61%) | 843 (88.36%) | 825 (86.47%) | 896 (93.91%) | 965 (90.71%) | 724 (75.88%) |
| Complete and single-copy BUSCOs(S) | 856 (89.73%) | 825 (86.48%) | 790 (82.81%) | 833 (87.32%) | 844 (88.48%) | 701 (73.44%) |
| Complete and duplicated BUSCOs(D) | 18 (1.89%) | 18 (1.89%) | 36 (3.75%) | 64 (6.68%) | 22 (2.31%) | 24 (2.49%) |
| Fragmented BUSCOs (F) | 21 (2.20%) | 29 (3.04%) | 66 (6.92%) | 24 (2.47%) | 20 (2.07%) | 90 (9.46%) |
| Missing BUSCOs (M) | 59 (6.18%) | 82 (8.60%) | 63 (6.63%) | 34 (3.56%) | 70 (7.34%) | 141 (14.81%) |
| Total Lineage BUSCOs | 954 | 954 | 954 | 954 | 954 | 954 |

**Table S7.** Gene prediction through integrating multiple methods.

| *Paragorgia papillata* | | | |
| --- | --- | --- | --- |
| Method | Software | Species | Gene number |
| *Ab initio* | Augustus | - | 37,728 |
|  | SNAP | - | 53,171 |
| Homology-based | GeMoMa | *Acropora digitifera* | 20,978 |
|  |  | *[Acropora millepora](https://www.ncbi.nlm.nih.gov/genome/2652)* | 25,569 |
|  |  | *Astreopora myriophthalma* | 23,033 |
|  |  | *Dendronephthya gigantea* | 23,859 |
|  |  | *Porites australiensis* | 22,738 |
|  |  | *Paramuricea clavata* | 44,026 |
|  |  | *[Stylophora pistillata](https://www.ncbi.nlm.nih.gov/genome/12040)* | 21,739 |
| RNAseq | PASA | - | 0 |
| Integration | EVM | - | 41,723 |
| *Chrysogorgia* sp. | | | |
| *Ab initio* | Augustus | - | 51,201 |
|  | SNAP | - | 72,233 |
| Homology-based | GeMoMa | *Acropora digitifera* | 21,413 |
|  |  | *[Acropora millepora](https://www.ncbi.nlm.nih.gov/genome/2652)* | 27,008 |
|  |  | *Astreopora myriophthalma* | 24,364 |
|  |  | *Dendronephthya gigantea* | 23,727 |
|  |  | *Porites australiensis* | 23,193 |
|  |  | *Paramuricea clavata* | 52,373 |
|  |  | *[Stylophora pistillata](https://www.ncbi.nlm.nih.gov/genome/12040)* | 22,853 |
| RNAseq | GeneMarkS-T | - | 3,460 |
|  | PASA | - | 2,562 |
| Integration | EVM | - | 52,329 |

**Table S8.** Functional annotation of *P. papillata* and *Chrysogorgia* sp. predicted gene.

|  | *P. papillata* | | *Chrysogorgia* sp. | |
| --- | --- | --- | --- | --- |
| Database | Annotated number | Percentage (%) | Annotated number | Percentage (%) |
| GO Annotation | 26,920 | 64.52 | 29,771 | 56.89 |
| KEGG Annotation | 26,727 | 64.06 | 31,257 | 59.73 |
| KOG Annotation | 15,371 | 36.84 | 17,943 | 34.29 |
| Pfam Annotation | 27,501 | 65.91 | 32,140 | 61.42 |
| Swissprot Annotation | 19,631 | 47.05 | 21,385 | 40.87 |
| TrEMBL Annotation | 36,959 | 88.58 | 46,059 | 88.02 |
| eggNOG Annotation | 22,068 | 52.89 | 25,191 | 48.14 |
| nr Annotation | 32,743 | 78.48 | 39,751 | 75.96 |
| All Annotated | 37,974 | 91.01 | 47,126 | 90.06 |

**Table S9.** Data on annotated genes from the *P. papillata*, *Chrysogorgia* sp. and other anthozoan used in this study.

|  | Genome size (Mb) | No. Predicted  Genes | Gene length  (bp) | Average gene length (bp) | Number of exons | Average exon length (bp) | Number of introns | Average intron length (bp) |
| --- | --- | --- | --- | --- | --- | --- | --- | --- |
| *P. papillata* | 618 | 41,723 | 254,688,447 | 6,104 | 216,272 | 275 | 174,549 | 1,118 |
| *Chrysogorgia* sp. | 781 | 52,329 | 314,127,140 | 6,003 | 233,360 | 313 | 181,031 | 1,332 |
| *P. clavata* | 712 | 62,652 | 243,255,127 | 3,883 | 274,793 | 290 | 212,143 | 772 |
| *D. gigantea* | 276 | 28,879 | 139,680,597 | 6,364 | 189,265 | 256 | 167,315 | 545 |
| *Trachythela* sp. | 578 | 35,305 | 229,708,091 | 5,592 | 221,362 | 271 | 180,289 | 941 |
| *A. digitifera* | 416 | 22,221 | 230,130,927 | 10,383 | 186,995 | 285 | 164,830 | 1,073 |
| *A. millepora* | 387 | 23,710 | 258,008,043 | 8621 | 202,147 | 330 | 172,219 | 1,111 |
| *S. pistillata* | 400 | 24,833 | 206,389,520 | 8256 | 193,726 | 266 | 168,726 | 918 |
| *A. fenestrafer* | 370 | 21,372 | 153,773,009 | 7,194 | 139,123 | 219 | 117,751 | 1,047 |
| *Discosoma* sp. | 445 | 23,199 | 160,255,849 | 6,907 | 138,376 | 226 | 115,177 | 1,119 |
| *Actinoscyphia* sp. | 522 | 29,975 | 230,103,153 | 7,677 | 230,788 | 325 | 197,543 | 968 |
| *N. vectensis* | 450 | 23,845 | 122,613,574 | 5,142 | 147,984 | 235 | 151,929 | 846 |

**Table S10.** The completeness of *P. papillata* and *Chrysogorgia* sp. gene by BUSCO assessment.

|  | *P. papillata* | *Chrysogorgia* sp. |
| --- | --- | --- |
| Complete BUSCOs(C) | 899 (94.23%) | 886 (92.87%) |
| Complete and single-copy BUSCOs(S) | 875 (91.72%) | 867 (90.88%) |
| Complete and duplicated BUSCOs(D) | 24 (2.52%) | 19 (1.99%) |
| Fragmented BUSCOs (F) | 19 (1.99%) | 22 (2.31%) |
| Missing BUSCOs (M) | 36 (3.77%) | 46 (4.82%) |
| Total Lineage BUSCOs | 954 | 954 |

**Table S11.** TEs constitution in *P. papillata* and *Chrysogorgia* sp. genome.

|  | *P. papillata* | | | *Chrysogorgia* sp. | | | |
| --- | --- | --- | --- | --- | --- | --- | --- |
| Type | Number | Length | Rate(%) | Type | Number | Length | Rate(%) |
| ClassI:Retroelement | 609,247 | 147,316,567 | 23.83 | ClassI:Retroelement | 786,727 | 188,836,259 | 24.18 |
| ClassI/DIRS | 434 | 222,331 | 0.04 | ClassI/DIRS | 616 | 341,408 | 0.04 |
| ClassI/LINE | 146,062 | 41,078,324 | 6.65 | ClassI/LINE | 215,734 | 60,966,089 | 7.81 |
| ClassI/LTR/Copia | 4,654 | 1,911,733 | 0.31 | ClassI/LTR/Copia | 3,219 | 1,690,045 | 0.22 |
| ClassI/LTR/ERV | 5,377 | 522,332 | 0.08 | ClassI/LTR/ERV | 6,262 | 633,525 | 0.08 |
| ClassI/LTR/Gypsy | 80,784 | 32,440,395 | 5.25 | ClassI/LTR/Gypsy | 106,221 | 39,942,941 | 5.11 |
| ClassI/LTR/Ngaro | 31,308 | 7,670,938 | 1.24 | ClassI/LTR/Ngaro | 14,775 | 3,641,810 | 0.47 |
| ClassI/LTR/Pao | 7,679 | 1,952,625 | 0.32 | ClassI/LTR/Pao | 2,385 | 555,920 | 0.07 |
| ClassI/LTR/Unknown | 316,571 | 59,126,695 | 9.57 | ClassI/LTR/Unknown | 402,391 | 75,661,718 | 9.69 |
| ClassI/SINE | 16,378 | 2,391,194 | 0.39 | ClassI/SINE | 35,124 | 5,402,803 | 0.69 |
| ClassII:DNA transposon | 603,893 | 146,886,297 | 23.76 | ClassII:DNA transposon | 674,941 | 186,207,636 | 23.84 |
| ClassII/Academ | 2,956 | 705,723 | 0.11 | ClassII/Academ | 1,967 | 753,023 | 0.1 |
| ClassII/CACTA | 4,820 | 595,309 | 0.1 | ClassII/CACTA | 9,092 | 1,308,685 | 0.17 |
| ClassII/Crypton | 4,341 | 1,191,651 | 0.19 | ClassII/Crypton | 3,208 | 920,640 | 0.12 |
| ClassII/Dada | 415 | 24,199 | 0 | ClassII/Dada | 1,175 | 129,362 | 0.02 |
| ClassII/Ginger | 544 | 44,904 | 0.01 | ClassII/Ginger | 1,121 | 112,885 | 0.01 |
| ClassII/Helitron | 2,195 | 1,181,712 | 0.19 | ClassII/Helitron | 4,693 | 2,144,738 | 0.27 |
| ClassII/IS3EU | 1,476 | 577,512 | 0.09 | ClassII/IS3EU | 2,790 | 619,622 | 0.08 |
| ClassII/Kolobok | 2,536 | 677,290 | 0.11 | ClassII/Kolobok | 6,020 | 1,466,504 | 0.19 |
| ClassII/Maverick | 10,054 | 7,213,074 | 1.17 | ClassII/MULE | 145 | 43,842 | 0.01 |
| ClassII/Merlin | 429 | 22,628 | 0 | ClassII/Maverick | 22,633 | 46,560,802 | 5.96 |
| ClassII/Mutator | 605 | 49,412 | 0.01 | ClassII/Merlin | 688 | 50,487 | 0.01 |
| ClassII/P | 3,776 | 1,297,980 | 0.21 | ClassII/Mutator | 1,210 | 143,977 | 0.02 |
| ClassII/PIF-Harbinger | 4,207 | 1,023,441 | 0.17 | ClassII/P | 1,765 | 595,838 | 0.08 |
| ClassII/PiggyBac | 180 | 12,377 | 0 | ClassII/PIF-Harbinger | 5,632 | 1,339,994 | 0.17 |
| ClassII/Tc1-Mariner | 3,954 | 1,203,005 | 0.19 | ClassII/PiggyBac | 280 | 22,321 | 0 |
| ClassII/Unknown | 549,103 | 127,760,926 | 20.67 | ClassII/Sola | 5 | 460 | 0 |
| ClassII/Zator | 141 | 40,688 | 0.01 | ClassII/Tc1-Mariner | 5,196 | 1,325,878 | 0.17 |
| ClassII/Zisupton | 155 | 43,742 | 0.01 | ClassII/Unknown | 585,894 | 124,710,135 | 15.97 |
| ClassII/hAT | 12,006 | 3,220,724 | 0.52 | ClassII/Zator | 140 | 15,787 | 0 |
| Unknown | 296 | 57,175 | 0.01 | ClassII/Zisupton | 443 | 53,027 | 0.01 |
| srpRNA | 2 | 579 | 0 | ClassII/hAT | 20,844 | 3,889,629 | 0.5 |
| Total | 1,213,438 | 294,260,618 | 47.60 | Unknown | 88 | 8,299 | 0 |
|  |  |  |  | srpRNA | 11 | 2,937 | 0 |
|  |  |  |  | Total | 1,461,767 | 375,055,131 | 48.02 |

**Table S12.** Summary statistics of repetitive sequences.

|  | *P. papillata* | *Chrysogorgia* sp. | *P. clavata* | *Trachythela* sp. | *D. gigantea* | *A. digitifera* |
| --- | --- | --- | --- | --- | --- | --- |
| DNA | 146,886,297 (49.95%) | 186,207,636 (49.69%) | 65,436,874 (20.87 %) | 80,115,138 (31.75%) | 6,344,179 (39.52%) | 24,991,328 (18.15%) |
| LINE | 41,078,324 (13.97%) | 60,966,089 (16.27%) | 33,645,094 (10.73 %) | 62,403,973 (24.73%) | 3,014,162 (18.78 %) | 18,533,837 (13.46%) |
| SINE | 2,391,194 (0.82%) | 5,402,803 (1.45%) | 9,570,518 (3.05 %) | 6,190,780 (2.46%) | 4,753 (0.03%) | 4,738,289 (3.45%) |
| LTR | 103,624,718 (35.24%) | 122,125,959 (32.59%) | 8,780,644 (2.81 %) | 22,107,531 (8.76%) | 6,435,444 (40.09%) | 19,414,404 (14.10%) |
| Unknown | 57,175 (0.02%) | 8,299 (0.00%) | 196,068,308 (62.54 %) | 81,497,366 (32.30%) | 253,480 (1.58%) | 69,984,103 (50.84%) |

**Table S13-S18 in the Supplementary TableS13-S18.xlsx**

**Reference：**

Baumgarten S, Simakov O, Esherick LY, Liew YJ, Lehnert EM, Michell CT, et al. 2015. The genome of *Aiptasia*, a sea anemone model for coral symbiosis. *Proc Natl Acad Sci U S A*, 2015;112:11893–98.

Behrensmeyer AK, Turner A. Taxonomic occurrences of Suidae recorded in the Paleobiology Database. Fossilworks [Internet]. 2013. Available from: <http://fossilworks.org.>

Buitrago-López C, Mariappan KG, Cárdenas A, Gegner HM, Voolstra CR. The genome of the Cauliflower coral *Pocillopora verrucosa*. Genome Biol Evol, 2020;12:1911–17.

Chapman JA, Kirkness E, Simakov O, Hampson SE, Mitros T, Weinmaier T, et al. The dynamic genome of *Hydra*. Nature, 2021;464:592–96.

Cunning R, Bay RA, Gillette P, Baker AC, Traylor-Knowles N. Comparative analysis of the *Pocillopora damicornis* genome highlights role of immune system in coral evolution. Sci Rep, 2018;8:16134.

Feng C, Liu R, Xu W, Zhou Y, Wang K. The genome of a new anemone species (Actiniaria: Hormathiidae) provides insights into deep-sea adaptation. Deep–Sea Research I, 2021;170:103492.

Jeon Y, Park SG, Lee N, Weber JA, Kim HS, Hwang SJ, et al. The draft genome of an octocoral, *Dendronephthya gigantea*. Genome Biol Evol, 2019;11:949–53.

Ledoux JB, Cruz F, Gómez-Garrido J, Antoni R, Blanc J, Gómez-Gras D, et al. The genome sequence of the octocoral *Paramuricea clavata* - a key resource to study the impact of climate change in the Mediterranean. G3-Genes Genomes Genetics, 2020;10:2941–52.

Putnam NH, Srivastava M, Hellsten U, Dirks B, Chapman J, Salamov A, et al. Sea anemone genome reveals ancestral Eumetazoan gene repertoire and genomic organization. Science, 2007;317:86–94.

Prada C, Hanna B, Budd AF, Woodley CM, Schmutz J, Grimwood J, et al. Empty niches after extinctions increase population sizes of modern corals. Curr Biol, 2016;26:3190–94.

Quattrini AM, Rodríguez E, Faircloth BC, Cowman PF, Brugler MR, Farfan GA, et al. Palaeoclimate ocean conditions shaped the evolution of corals and their skeletons through deep time. Nat Ecol Evol, 2020;4:1531–38.

Shinzato C, Khalturin K, Inoue J, Zayasu Y, Kanda M, Kawamitsu M, et al. Eighteen coral genomes reveal the evolutionary origin of *Acropora* strategies to accommodate environmental changes. Mol Biol Evol, 2020;38:16–30.

Voolstra CR, Li Y, Liew YJ, Baumgarten S, Zoccola D, Flot JF, et al. Comparative analysis of the genomes of *Stylophora pistillata* and *Acropora digitifera* provides evidence for extensive differences between species of corals. Sci Rep, 2017;7:17583.

Wang X, Liew YJ, Li Y, Zoccola D, Tambutté S, Aranda M. Draft genomes of the corallimorpharians *Amplexidiscus fenestrafer* and *Discosoma* sp. Mol Ecol Resour, 2017;17:e187–e195.

Ying H, Hayward DC, Cooke I, Wang W, Moya A, Siemering KR, et al. The whole-genome sequence of the coral *Acropora millepora*. Genome Biol Evol, 2019;11:1374–79.

Zhou Y, Feng C, Pu Y, Liu J, Liu R, Zhang H. The first draft genome of a cold-water coral *Trachythela* sp. (Alcyonacea: Stolonifera: Clavulariidae). Genome Biol Evol, 2021;13:evaa265.
