# Supplementary material for: The molecular basis of octocoral calcification revealed by genome and skeletal proteome analyses
Source: Gigascience. 2025 Apr 1;14:giaf031. doi: 10.1093/gigascience/giaf031 (PMC11959691; doi:10.1093/gigascience/giaf031)
Supplement: giaf031_GIGA-D-24-00546_Original_Submission [file giaf031_giga-d-24-00546_original_submission.pdf]

GigaScience

The molecular basis of octocoral calcification revealed by genome and skeletal proteome analyses

--Manuscript Draft--

**Manuscript Number:**	GIGA-D-24-00546
**Full Title:**	The molecular basis of octocoral calcification revealed by genome and skeletal proteome analyses
**Article Type:**	Data Note
**Funding Information:**	National Natural Science Foundation of China (No. 41930533) — Prof. Kuidong Xu<br>Strategic Priority Research Program of the Chinese Academy of Sciencescs, Chinese Academy of Sciences (No. XDB42000000) — Prof. Kuidong Xu<br>Senior User Project of R/V KEXUE of the Chinese Academy of Sciences (No. KEXUE2020GZ02) — Prof. Kuidong Xu
**Abstract:**	The ability of octocorals and stony corals to deposit calcium carbonate (CaCO3) have led to their ecological success. Compared with the homogeneous aragonite skeleton of stony corals, octocorals have evolved different skeletal structures composed of different CaCO3 polymorphs and skeletal organic matrix. However, the molecular basis of skeletal structure formation in octocorals remains largely unexplored. Here, we generated the genomes and skeletal proteomes of two calcite-forming octocorals, Paragorgia papillata and Chrysogorgia sp. Both assembled genomes size is 618.13 Mb and 781.04 Mb for P. papillata and Chrysogorgia sp. respectively, with contig N50 of 2.67 Mb and 2.61 Mb. Comparative genomic analyses identified 162 and 285 significantly expanded gene families in the genomes of P. papillata and Chrysogorgia sp., respectively, which were mainly associated with biomineralization and immune response. Comparative analyses of skeletal proteomes revealed that corals with different CaCO3 polymorphs share a basic toolkit consisting of cadherin, von Willebrand factor type A and carbonic anhydrase domains for calcified skeleton deposition. By contrast, collagen is rich in the calcite-forming octocoral skeletons, but rarely occurs in aragonitic stony corals. Furthermore, some collagens have evolved domains related to matrix adhesion and immunity, which may confer new genetic functions on the calcification in octocorals. These findings facilitate our comprehension of the diverse forms of coral biomineralization and provide preliminary insights into the formation and evolution of the octocoral skeleton.
**Corresponding Author:**	Yanshuo Liang<br>Institute of Oceanology Chinese Academy of Sciences<br>Qingdao, CHINA
**Corresponding Author Secondary Information:**	
**Corresponding Author's Institution:**	Institute of Oceanology Chinese Academy of Sciences
**Corresponding Author's Secondary Institution:**	
**First Author:**	Yanshuo Liang
**First Author Secondary Information:**	
**Order of Authors:**	Yanshuo Liang<br>Kuidong Xu<br>Junyuan Li<br>Jingyuan Shi<br>Jiehong Wei

Powered by Editorial Manager® and ProduXion Manager® from Aries Systems Corporation

	Xiaoyu Zheng
	Wanying He
	Xin Zhang
**Order of Authors Secondary Information:**	
**Additional Information:**	
**Question**	**Response**
Are you submitting this manuscript to a special series or article collection?	No
**Experimental design and statistics**<br><br>Full details of the experimental design and statistical methods used should be given in the Methods section, as detailed in our Minimum Standards Reporting Checklist. Information essential to interpreting the data presented should be made available in the figure legends.<br><br>Have you included all the information requested in your manuscript?	Yes
**Resources**<br><br>A description of all resources used, including antibodies, cell lines, animals and software tools, with enough information to allow them to be uniquely identified, should be included in the Methods section. Authors are strongly encouraged to cite Research Resource Identifiers (RRIDs) for antibodies, model organisms and tools, where possible.<br><br>Have you included the information requested as detailed in our Minimum Standards Reporting Checklist?	Yes
**Availability of data and materials**<br><br>All datasets and code on which the conclusions of the paper rely must be either included in your submission or deposited in publicly available repositories (where available and ethically	Yes

Powered by Editorial Manager® and ProduXion Manager® from Aries Systems Corporation

appropriate), referencing such data using a unique identifier in the references and in the "Availability of Data and Materials" section of your manuscript.<br><br>Have you have met the above requirement as detailed in our Minimum Standards Reporting Checklist?	
GigaScience has policies and guidelines in place for the use of generative AI-writing tools such as ChatGPT. If you have used such writing tools to assist with writing the manuscript this must be declared and cited in the text. Authors should not list AI-writing tools and other AI-assisted technologies as an author or co-author and should acknowledge that they are fully responsible for text generated or refined by AI-writing tools.<p><br><br>A summary of use (particularly in the introduction or among methods) needs to be included at the end of the paper, and the outputs should also be included as a supplementary file hosted in GigaDB or other open repositories. Please read our guidelines for more information. <p><br><br>By submitting to GigaScience, you are aware of the journal's AI-writing tools policy, and if you have declared use of such tools below, you have acknowledged this where appropriate in your manuscript and have made a summary of use and outputs available. <p><br><b>AI-assisted writing tools have been used in the preparation of this manuscript?	No

Click here to access/download;Manuscript;Manuscript.docx

The molecular basis of octocoral calcification revealed by genome and skeletal proteome analyses

Yanshuo Liang[1,2,3], Kuidong Xu[1,2,3*], Junyuan Li[1,2], Jingyuan Shi[4], Jiehong Wei[1,2,3], Xiaoyu Zheng[1,2,3], Wanying He[3,5], Xin Zhang[3,5]

[1]*Laboratory of Marine Organism Taxonomy and Phylogeny, Qingdao Key Laboratory of Marine Biodiversity and Conservation, Institute of Oceanology, Chinese Academy of Sciences, Qingdao 266071, China*

[2]*Laoshan Laboratory, Qingdao 266237, China*

[3]*University of Chinese Academy of Sciences, Beijing 100049, China*

[4]*CAS Key Laboratory of Marine Ecology and Environmental Sciences, Institute of Oceanology, Chinese Academy of Sciences, Qingdao 266071, China*

[5]*Key Laboratory of Marine Geology and Environment & Center of Deep Sea Research, Institute of Oceanology, Center for Ocean Mega-Science, Chinese Academy of Sciences, Qingdao 266071, China*

***Correspondence address**. Kuidong Xu. No. 7 Nanhai Road, Qingdao City, Shandong Province, China. Institute of Oceanology, Chinese Academy of Science. E-mail: kxu@qdio.ac.cn

Abstract

The ability of octocorals and stony corals to deposit calcium carbonate ($CaCO_3$) have led to their ecological success. Compared with the homogeneous aragonite skeleton of stony corals, octocorals have evolved different skeletal structures composed of different $CaCO_3$ polymorphs and skeletal organic matrix. However, the molecular basis of skeletal structure formation in octocorals remains largely unexplored. Here, we generated the genomes and skeletal proteomes of two calcite-forming octocorals, *Paragorgia papillata* and *Chrysogorgia* sp. Both assembled genomes size is 618.13 Mb and 781.04 Mb for *P. papillata* and *Chrysogorgia* sp. respectively, with contig N50 of 2.67 Mb and 2.61 Mb. Comparative genomic analyses identified 162 and 285 significantly expanded gene families in the genomes of *P. papillata* and *Chrysogorgia* sp., respectively, which were mainly associated with biomineralization and immune response. Comparative analyses of skeletal proteomes revealed that corals with different $CaCO_3$ polymorphs share a basic toolkit consisting of cadherin, von Willebrand factor type A and carbonic anhydrase domains for calcified skeleton deposition. By contrast, collagen is rich in the calcite-forming octocoral skeletons, but rarely occurs in aragonitic stony corals. Furthermore, some collagens have evolved domains related to matrix adhesion and immunity, which may confer new genetic functions on the calcification in octocorals. These findings facilitate our comprehension of the diverse forms of coral biomineralization and provide preliminary insights into the formation and evolution of the octocoral skeleton.

Keywords Octocorallia, genomes, $CaCO_3$ polymorphs, skeletal proteomes, biomineralization toolkit

Introduction

The history of biomineralization of $CaCO_3$ by organisms has lasted for at least 541 Myr, and biomineralization as an innovation in the history of life has played a significant role in species evolution and global carbon cycles [1]. As an ecologically important and morphologically diverse clade of metazoans, the class Anthozoa create enormous biogenic structures by their ability to form colonies and precipitate $CaCO_3$ skeletons to support entire coral ecosystems in both shallow and deep waters. $CaCO_3$ skeleton-producing ability is found in two distinct clades of Anthozoa, namely the order Scleractinia (stony coral, subclass Hexacorallia) and the subclass Octocorallia (octocoral). As the main reef builders, stony corals have homogeneous aragonite skeleton, and their calcification process has been interpreted by the analysis of skeletal proteomes and immunohistochemical verification [2–5]. In contrast, octocorals have evolved a wide variety of skeletal structures, mainly including different $CaCO_3$ polymorphs (i.e., aragonite or calcite) and organic components (e.g., gorgonin) as well as different types of sclerites [6, 7]. Consequently, octocoral skeletons provide a unique opportunity to compare different calcification strategies involving different skeletal structures and $CaCO_3$ polymorphs with stony corals.

The formation of coral skeletons is biologically controlled by the supply of ions required for $CaCO_3$ deposition and the secretion of diverse organic matrix to calcification site (Supplementary Fig. S1) [8–10]. Major components of organic matrix include proteins, carbohydrates and lipids [6, 8]. The organic matrices secreted by the calicoblastic ectoderm play an important role in promoting nucleation, growth, and spatial orientation of different $CaCO_3$ polymorphs even though they constitute only a very minor proportion of the coral skeletal organic matrix space [11]. Previous study showed that, although a set of conserved proteins for biomineralization exists in

mollusks, the calcite and aragonitic layers within the shell respectively use specific shell matrix proteins to deposit different polymorphs [12]. The core question surrounding coral calcification is how corals regulate the calcite and aragonitic polymorphs through skeletal organic matrix proteins (SOMPs), and how they control the growth of complex and diverse skeletal structures. However, the lack of genome and proteome in octocorals has limited our understanding of the molecular mechanisms underlying the formation of different $CaCO_3$ polymorphs skeletal structures.

In this study, we generated draft genomes of two calcite-forming octocorals (*Paragorgia papillata* and *Chrysogorgia* sp.) and characterized the skeletal proteomes of octocorals. We further performed phylogenetic analyses, gene family expansion and contraction analyses, and comparative skeletal proteomic analyses to understand the molecular basis of skeleton formation in octocorals. The availability of these genomic and proteome information provides a valuable resource for understanding the molecular mechanism of coral skeletal formation and its evolutionary history.

Methods

Sample collection and DNA extraction

Specimens of *P. papillata* and *Chrysogorgia* sp. were collected by the submersible vehicles *Faxian* and *Jiaolong* from seamounts of the Caroline Ridge (10°06'46.80"N, 140°14'31.79"E, 858 m deep) and the Kyushu-Palau Ridge (13°20'18.24"N, 134°33'37.44"E, 2,086 m deep) in the tropical Western Pacific (Fig. 1A, 1B). The coral samples were kept in a closed sample chamber placed inside the sample basket of the submersible. Following recovery, the samples were cut into small pieces and immediately stored in liquid nitrogen. All experimental protocols were approved

by the relevant guidelines and regulations established by the Institutional Animal Care and Use Committee of the Institute of Oceanology, Chinese Academy of Science. The polyps were used to extract genomic DNA with a MagAttract HMW DNA kit (Qiagen, Germany). The quality and quantity of the extracted DNA were checked with standard agarose gel electrophoresis and a Qubit Fluorometer, respectively.

Illumina sequencing and genome size estimation

Paired-end libraries with insert sizes of 300 base pairs were constructed using the TruSeq DNA Sample Prep Kit, following the manufacturer's instructions. The resulting libraries were then sequenced on an Illumina NovaSeq 6000 platform. The low-quality and sequencing-adapter-contaminated reads were trimmed using Trimmomatic-0.36 (RRID:SCR_011848).

A K-mer frequency distribution map of the clean reads was constructed to estimate the genome size, heterozygosity and proportion of repetitive sequences using the GenomeScope software (RRID:SCR_017014) [13]. The genome size (G) was estimated using the following formula: $G = K_{num}/K_{depth}$, where K_{num} is the number of K-mers and K_{depth} is the peak depth. The trimmed Illumina paired-end reads were assembled as contigs using SOAPdenovo v2.04 (RRID:SCR_010752) [14] with the default parameters. This was followed by extending, gap filling and polishing the assembly.

PacBio sequencing and genome assembly

The high-molecular-weight genomic DNA (gDNA) was employed in the construction of Pacific

Biosciences (PacBio) sequencing libraries. The gDNA was fragmented by the g-TUBE device (Covaris) to a size of 6–20 kb for the construction of 20 kb libraries. The sheared DNA was then concentrated and purified using AMPure XP Beads (Agencourt). The sequencing reagents included in the SMRTbell Template Prep Kit were employed for the repair of abasic sites, nicks, thymine dimers, blocked 3'-ends, oxidised guanines/pyrimidines and deaminated cytosines. T4 DNA polymerase was employed for the purpose of polishing the ends of fragments deemed suitable for ligation. The SMRTbell hairpin adapters were ligated to the repaired ends. Subsequently, size selection was conducted using BluePippin electrophoresis (Sage Science), with a cutoff threshold size of 20 kb. Following this, AMPure PB Beads were employed to concentrate and purify the SMRTbell templates after size selection. Finally, these purified SMRTbell templates were utilized for primer and polymerase binding. The SMRTbell libraries were then sequenced on a Pacbio Seque II platform (RRID:SCR_017990).

Following the removal of low-quality and duplicate reads, the Pacbio clean reads were corrected using the error correction module of Canu v.1.5 (RRID:SCR_015880) [15] to select for longer subreads. Contaminated reads containing chloroplast, mitochondrial, bacterial or viral sequences were removed via comparison of the genome assembly with the nucleotide sequence database (nt) from the National Center for Biotechnology Information (NCBI). Subsequently, the data were assembled by NextDenovo v.2.2 (RRID:SCR_025033) with the default parameters. Next, the raw assembly was polished on three rounds with Illumina short reads using Pilon (RRID:SCR_014731) [16]. Finally, the PacBio reads were aligned to the initial assembly using minimap2 v.2.24-r1122 (RRID:SCR_018550) with the parameter: -x map-bp. Duplicated genes in the assembly were removed by using Purge_dups v.1.2.5 (RRID:SCR_021173) [17] with the

parameter minimumAlignmentScore 70 for *P. papillata* and minimumAlignmentScore 80 for *Chrysogorgia* sp. To evaluate the accuracy of the genome assembly, the Illumina reads were first mapped to the genome assembly using bwa v.0.7.10 (RRID:SCR_010910). Furthermore, the completeness of the genome assembly was evaluated by mapping 954 metazoan benchmarking universal single-copy orthologues to the genome by using BUSCO v.5.0 (RRID:SCR_015008) [18].

Transcriptome sequencing

Total RNA was extracted from polyps of *P. papillata* and *Chrysogorgia* sp. using Invitrogen TRIzol Reagent (Thermo Fisher Scientific), following the manufacturer's instructions. The integrity and quality of the RNA were evaluated using the Fragment Analyzer 5400 (Agilent Technologies). Sequencing libraries were generated using the NEBNext® UltraTM RNA Library Prep Kit for Illumina® (NEB, USA) following manufacturer's instructions, with an insert size of 300–500 bp. Illumina RNA-seq libraries were prepared and sequenced on an Illumina Novaseq 6000 platform, resulting in 150 bp paired-end reads. Following the application of quality score-based trimming using Trimmomatic-0.36, clean reads were aligned to the coral genomes with StringTie v.2.1.5 (RRID:SCR_016323) [19].

Genome annotation

The protein-coding genes were annotated using a combination of *ab initio* prediction methods, and homology searches, and RNA sequencing (RNA-seq). *Ab initio* gene prediction was conducted using Augustus v.3.1.0 (RRID:SCR_008417) and SNAP v.2006-07-28 (RRID:SCR_007936) with

the default parameters. For the homolog-based approach, GeMoMa v.1.7 (RRID:SCR_017646) [20] software was performed by using reference gene model from the other cnidarians, that is *A. digitifera*, *A. millepora*, *Astreopora myriophthalma*, *D. gigantea*, *Porites australiensis*, *P. clavata*, *Stylophora postillata*. Gene prediction based on RNA-seq data was conducted by aligning clean RNA-seq reads to the reference genome using Hisat2 v.2.0.4 (RRID:SCR_015530) [21] and assembling them with StringTie v.2.1.5. The coding regions were predicted using GeneMarkS-T v.5.1 (RRID:SCR_017648) [22] and PASA v.2.0.2 (RRID:SCR_014656) [23]. Gene models from these different approaches were combined using the EVM v1.1.1 (RRID:SCR_014659) [24] and updated by PASA. The final gene models were annotated by blasting the GenBank Non-Redundant, Gene Ontology, KEGG and SwissProt database, with an E-value cut-off of 1×10^{-5}. Moreover, these predicted genes were annotated against the Pfam database of the HMMER v.3.3.2 (RRID:SCR_005305) software (http://www.hmmer.org/).

Transposable elements (TEs) analysis was performed by the RepeatModeler pipeline v.2.0.1 (RRID:SCR_015027) [25] and LTR_retriever v.2.9.0 (RRID:SCR_017623) [26]. We first used RECON v.1.0.8 (RRID:SCR_021170), RepeatScout v.1.0.6 (RRID:SCR_014653), LTRharvest v.1.5.10 (RRID:SCR_018970) and LTR_FINDER v.1.0.7 (RRID:SCR_015247) to construct a *de novo* repeat library with the default settings. The predicted repeats were classified using RepeatClassifier and combined with the Dfam database v.3.5. Finally, RepeatMasker v4.1.2 (RRID:SCR_012954) [27] was used to identify the divergence of TEs in the coral genomes based on the constructed repetitive sequences database, and a repeat landscape was obtained using an R script that was modified from https://github.com/ValentinaBoP/TransposableElements.

Phylogenetic analysis, gene expansion and contraction

The orthologue groups (OGs) were identified through a BLASTp search of protein sequences from 19 anthozoans and *Hydra vulgaris* (outgroup) genomes (Supplementary Table S1). The BLASTp results were used to assign the OGs by OrthoFinder v2.4.0 (RRID:SCR_017118) [28]. To construct phylogenetic relationships, the protein sequences of 275 single-copy orthologues were extracted from all 20 species and multiple alignment analysis was performed with MAFFT v7.310 (RRID:SCR_011811). Poorly aligned regions were trimmed using Gblocks v0.91b (RRID:SCR_015945) and all alignments were combined into one supergene. The ModelFinder software was used to identify the best suitable model of the trimmed alignment, and the maximum likelihood tree was generated using IQtree v2.2.0 (RRID:SCR_017254) [29] with 1,000 bootstrap replicates. The divergence times were estimated using MCMCTree from the PAML v.4.9j (RRID:SCR_014932) [30] with a correlated rates molecular clock. Five fossil calibration points (Supplementary Table S2) were selected for dating the phylogeny of anthozoans. Finally, the OGs comprising >100 copies in a single species were excluded, and the remaining OGs were employed for the gene family expansion and contraction analysis using CAFÉ v4.2.1 (RRID:SCR_005983) [31] with the parameter lambda -s and estimated divergence times between species as input. Only those with gene family wide *p*-value lower than 0.01 and a taxon-specific Viterbi *p*-value lower than 0.05 were considered as an event of significant expansion or contraction. The significantly expanded and contracted gene families were extracted for the GO term enrichment analysis with Fisher's exact test, and the *p*-value was adjusted for multiple testing using the False Discovery Rate method.

Morphological observation, $CaCO_3$ polymorphs analysis and van Gieson staining of octocoral skeletons

To observe the skeletal ultrastructure, the axial skeletons of *P. papillata* and *Chrysogorgia* sp. were isolated by digestion of the tissues in sodium hypochlorite, and then were washed repeatedly with milli-Q water repeatedly. Then, the axial skeletons were transferred to carbon double-adhesive tape, air-dried and coated for analysis by the scanning electron microscopy (SEM). SEM scans were obtained using a Hitachi TM3030Plus SEM at 15 kV and the optimum magnification for each axial skeleton. To investigate the $CaCO_3$ polymorphs of coral skeletons, we used the confocal Raman spectroscopy technology (Alpha 300R+, WITec, Ulm, Germany) to detect the axial skeleton after the removal of the coenenchyme. In order to observe the distribution of collagen fibers in axial skeletons, we conducted the van Gieson (VG) staining experiments. The specific procedures were as follows: Firstly, the decalcified axial skeleton was embedded in paraffin, dewaxed with xylene and ethanol, and stored in tap water. Secondly, the samples were stained with VG staining solution (Servicebio) for 1 min, rinsed rapidly with water, and dehydrated rapidly in anhydrous ethanol triple. Finally, the slides were immersed in xylene until transparent and then coverslipped with neutral resin, observed under a microscope and photographed.

Proteomic analysis

The axial skeletons of *P. papillata* and *Chrysogorgia* sp. were bleached in 10% hypochlorite for 5 h to remove the tissue and other potential contaminants. Following this, the skeletons were rinsed extensively with milli-Q water and left to dry overnight at 60°C. The dried axis skeletons were

ground to a fine powder in liquid nitrogen and again bleached, rinsed, and dried. The skeleton powder was decalcified with 10% acetic acid for 24 h at room temperature on an orbital shaker and the decalcified solution was centrifuged (14000 × g, 10 min, 4°C) to separate the acid soluble (ASM) and insoluble matrix (AIM). The obtained insoluble pellets (AIM) were rinsed several times with milli-Q water, lyophilised and reconstituted with 8 M of urea (with 1% SDS). Both AIM and ASM were concentrated using Amicon Ultrafiltration devices (15 ml, 10 kDa cutoff), purified with methanol/chloroform and subsequently reconstituted in 8 M of urea.

The ASM and AIM samples were dissolved in solubilization buffer (1% SDS, 10 mM DTT, 50 mM Tris-HCl (pH 8.0)) for sodium dodecyl sulfate-polyacrylamide gel electrophoresis (SDS-PAGE). The AIM and ASM were prepared for HPLC-MS/MS analysis after reduction, alkylation, trypsin digestion, drying and solubilization. Label-free mass spectrometry was conducted using a Thermo Orbitrap Fusion mass spectrometer. The scan events were configured as a full MS scan of 250–1450 m/z at a mass resolution of 120,000, followed by CID MS/MS scan repetition on the 20 most intense ions selected from the previous full MS scan with an isolation window. The resulting MS raw data were imported into MaxQuant v1.5.2.8 (RRID:SCR_014485) [32], and searched against their respective genomic data. Proteins with a spectral count of more than 2 in each sample were considered to be identified in this study. Proteins identified with at least two distinct peptides were considered for the analysis.

Protein annotation was performed by sequence similarity protein searches against the NR database in NCBI and UniProtKB/Swiss-Prot database. Protein sequences were analyzed for the presence of signal peptides with Signal IP v5.0 (https://services.healthtech.dtu.dk/services/SignalP-5.0/) and TM domains with TMHMM v2.0

(https://services.healthtech.dtu.dk/services/TMHMM-2.0/). Conserved domains were detected using the InterproScan platform (http://smart.embl.de/, last accessed April 2023). In previous studies, protein identification was based on matching nucleotide or EST databases with unique peptides, and such functional annotations were incomplete. We performed a comparative analysis of the domain of SOMPs, by including these two octocorals and two aragonitic scleractinians (*Acropora millepora* and *Stylophora pistillata*) [3, 4]. Interspecies comparison of the SOMPs from each species was carried out using locally installed NCBI BLAST tool (version 2.2.25+).

Results

Genomic characteristics of *P. papillata* and *Chrysogorgia* sp.

Using a combination of PacBio long reads and Illumina short reads (Supplementary Fig. S2 and Supplementary Table S3), we generated high-quality genomes for *Paragorgia papillata* and *Chrysogorgia* sp. The genome sizes for *P. papillata* (618.13 Mb) and *Chrysogorgia* sp. (781.04 Mb) are in close agreement with the K-mer-based estimates of 596.50 Mb and 774.93 Mb, respectively (Supplementary Fig. S3 and Supplementary Table S4). The contig N50 of the *P. papillata* assembly are 2.67 Mb, while they are 2.61 Mb for the *Chrysogorgia* sp. assembly (Supplementary Table S4). To evaluate the integrity of the assembled genomes, one library of paired-end data for each coral was back-mapped to the assembly. A total of 99.34% (*P. papillata*) and 99.40% (*Chrysogorgia* sp.) of the Illumina paired-end reads could be aligned to the assembled genomes (Supplementary Table S5). The benchmarking universal single-copy orthologs (BUSCO) analysis with the metazoan database showed that the genome assemblies of *P. papillata* and *Chrysogorgia* sp. exhibited 91.61% and 88.36% complete BUSCO genes, respectively

(Supplementary Table S6). These results indicate that the assembled genomes are highly accurate and complete.

The genomes of *P. papillata* and *Chrysogorgia* sp. have a relatively high number of protein-coding genes compared to other anthozoans. A total of 41,723 and 52,329 protein-coding genes were identified in *P. papillata* and *Chrysogorgia* sp., respectively, through integrating multiple methods (Supplementary Table S7), with 91.01% and 90.06% of genes annotated into Nr, GO, KEGG and other databases (Supplementary Table S8). Interestingly, the number of protein-coding genes in the octocorals is relatively more variable than that in hexacorals (Supplementary Table S9), which may be associated with their diverse habitat. Genome annotation completeness was also evaluated using BUSCO, and the results showed that the ortholog genes of *P. papillata* and *Chrysogorgia* sp. contributed 94.23% and 92.87% of complete genes, respectively (Supplementary Table S10), indicating that our gene annotation is highly complete. Transposable elements (TEs) drive genome evolution by altering the genomic architecture and affecting gene expression regulation. Using a combination of homology-based and *de novo* approaches, the TEs of 294.04 and 374.71 Mb are identified in the *P. papillata* and *Chrysogorgia* sp. genomes (47.58% and 47.98%, respectively) (Fig. 1C; Supplementary Table S11 and S12), with both class II DNA transposons predominate (23.76% and 23.84%, respectively), while class I retrotransposons (long interspersed nuclear elements (LINEs), long terminal repeats (LTRs) and short interspersed nuclear elements (SINEs)) account for 23.81% and 24.14% of the genomes, respectively. Furthermore, Kimura distance-based copy divergence analysis showed that the TEs of different coral lineages had similar expansion patterns except *Trachythela* sp. (Tsp) and were highly similar in composition (Fig. 1C; Supplementary Fig. S4).

Phylogenomic analysis and gene-family evolution

Phylogenetic results unequivocally showed an Ediacaran origin for Anthozoa and the reciprocal monophyly of the subclasses Octocorallia and Hexacorallia (Fig. 1D). The five octocorals analyzed were assigned to two newly established orders Scleralcyonacea (Ppap and Csp) and Malacalcyonacea (Pcla, Tsp and Dgig). *P. papillata* (Ppap) and *Chrysogorgia* sp. (Csp) formed a sister group, and their divergence was estimated at around the Triassic-Jurassic boundary (181 Ma), that is, in the transition period from aragonitic to calcite seas. The other three octocorals, *Dendronephthya gigantea* (Dgig), *Paramuricea clavata* (Pcla), and *Trachythela* sp. (Tsp), also originated in calcite seas during the Jurassic to Cretaceous period. Furthermore, our results supported the monophylies of Actiniaria (true sea anemones), Corallimorpharia (naked corals, mushroom anemones), and Scleractinia (stony corals) within Hexacorallia. The differentiation between Scleractinia and Corallimorpharia (Afen and Dsp) dated back to 281 Ma (95% confidence interval 349–221 Ma). Stony corals evolved the ability to deposit aragonitic crystals in typical aragonitic seas during the Late Carboniferous to Triassic periods (281–214 Ma). Subsequently, stony corals diversified into two crown clades (‘robust’ and ‘complex’) in the aragonitic seas during the mid-Triassic period (228–193 Ma).

Comparative analyses among the available anthozoan genomes showed that 286 gene families were expanded in *P. papillata* and 444 in *Chrysogorgia* sp., with 162 and 285 gene families significantly expanded, respectively (Viterbi p-value < 0.05) (Fig. 1D; Supplementary Table S13-S14). The GO enrichment analysis on the expanded gene families revealed 24 overrepresented GO categories in the *P. papillata* and *Chrysogorgia* sp. genomes (Supplementary

Fig. S5). The significantly expanded gene families appear to be involved in the phosphatidylinositol signaling pathway (PIP5 kinase activity and G protein-coupled neurotransmitter receptor), cell-cell adhesion (cadherin binding and actinin binding), ion transport process (potassium channel regulator, vacuolar transport and endosomal transport), and immune-related pathway (e.g. scavenger receptor activity, immunoglobulin production, and T cell receptor signaling pathway), suggesting their contributions to both biomineralization and immune response.

Skeletal structure characterization and biomineralized protein toolkit

To determine the types of the octocoral skeletons, we utilized Raman spectroscopy and scanning electron microscopy to analyze the axial skeletons. The axial skeleton of *P. papillata* was found to consist of the accumulation of high-Mg calcite (HMC) sclerites of varying morphologies, forming a ring of regularly arranged central pores. In contrast, the axial skeleton of *Chrysogorgia* sp. is a fully calcified, HMC structure with a growth pattern analogous to that of annual rings (Fig. 2; Supplementary Fig. S6).

We further investigated the molecular basis of the skeletal formation in the octocorals and identified a total of 64 and 37 SOMPs in the skeletal organic matrix space of *P. papillata* and *Chrysogorgia* sp., respectively (Supplementary Table S15-S16), using LC/MS/MS protein sequencing and reference genome searches. All these SOMPs were supported by more than one unique peptide (Supplementary Table S17-S18). To characterize the conserved biomineralization toolkit, we performed a comparative skeleton proteomic analysis of the two calcite-forming octocorals and the two aragonitic scleractinians, *A. millepora* and *S. pistillata*. To detect multiple

domains in the same protein from different evolutionary sources, we performed domain predictions and further compared the SOMPs in their functional context. Despite the significant differences in the coral skeletal morphology and microstructure, we identified three functional domains that are common to the coral skeletal organic matrix space of all the four species (Fig. 2). These domains are cadherin, von Willebrand factor type A (VWA) and carbonic anhydrase (CA). The cadherin domain, which contains conserved cysteine residues and calcium-binding motifs involved in intercellular adhesion, and this domain was only detected in protocadherin-like or classical cadherin (Supplementary Table S15-S16), which belong to the cadherin superfamily. The VWA domain was identified in protocadherin, collagen and fibrillin-2 (Supplementary Table S15-S16). CA formed a superfamily of mostly zinc-binding metalloenzymes that catalyze the interconversion of CO_2 into HCO_3^-, all of which contain predicted signal peptides or transmembrane domains (Supplementary Table S15-S16).

Function and composition of SOMPs

The SOMPs enclosed in the octocoral skeletons can be classified into five main categories based on the domain function prediction, namely, cell adhesion, structure support, immune regulation, enzymes, and other functional proteins (Fig. 2; Supplementary Table S15-S16). The composition of SOMPs varied greatly among corals with different skeletal types, and each coral retained the specific functional domains, with the ratio of unique functional domains up to 47% in *P. papillata* (Fig. 2). In comparison with the stony corals, we identified a large number of proteins containing immunity-associated domains in octocorals, including alpha-2-macroglobulin, spondin 2, agrin, and putative defence protein 3 (Fig. 2; Supplementary Table S15-S16). The occurrence of these

proteins is consistent with the expansion of gene families related to immune regulation (Supplementary Fig. S5), suggesting the existence of immune regulatory pathways within the coral skeletal organic matrix that reinforce the defence mechanisms of skeletal formation and prevent pathogen invasion.

We identified 7 and 5 types of collagen in the skeletons of *P. papillata* and *Chrysogorgia* sp., respectively and the helical regions of all collagens exhibited the typical Gly-X-Y periodic repeats (Fig. 3A; Supplementary Table S15-S16). Pfam domain analysis revealed that some collagens have undergone recombination with VWA, WAP, and laminin G domains, which may contribute to the diverse functions of collagens. To observe the distribution of collagen fibers in tissues, we further performed VG staining on the axial skeleton of *P. papillata* and *Chrysogorgia* sp. The result showed that the collagen fibers in *P. papillata* were mainly distributed on the calcified sclerites along its unconsolidated and unfused scleritic axis, while the collagen fibers in *Chrysogorgia* sp. were dyed deep red throughout the axial skeleton, indicating its widespread distribution in the mineralized skeleton (Fig. 3B, 3C; Supplementary Fig. S7).

Discussion

In this study, we sequenced and assembled draft genomes and skeletal proteomes for the two octocoral species, *P. papillata* and *Chrysogorgia* sp., which enrich the expanding list of octocoral genomes and provide valuable resources for advancing our understanding of the skeletal formation and evolutionary history of octocorals. We found that, with the exception of *P. clavata*, the genome sizes of these two octocorals were considerably larger than those of the published genomes of the octocorals and hexacorals, which may be caused by the large number of repetitive

sequences in the genomes of *P. papillata* and *Chrysogorgia* sp. Phylogenetic analyses show that the five calcite-forming octocorals analyzed were assigned to two newly established orders Scleralcyonacea and Malacalcyonacea [33], and that they all originated in calcite seas originating from the Jurassic to the Cretaceous period. The origin of corals with different $CaCO_3$ polymorphs is generally considered to be related to the palaeoclimate ocean conditions [34], and higher Mg/Ca ratios in calcite seas may have facilitated calcite skeleton formation in octocorals [35].

The skeletal structure of corals contains an embedded organic matrix with a set of proteins that can stabilize amorphous calcium carbonate and control the nucleation, orientation and polymorph selection [9, 36]. Understanding the SOMPs composition of the coral skeleton is important for elucidating the ancient mechanisms underlying the coral skeleton formation and evolution. By comparing the proteomes of different coral skeletons, we shed light on a conserved protein toolkit used by calcite-forming octocorals and aragonitic stony corals for biomineralization. Without considering the differences in the skeletal morphology and polymorphs, the biomineralization toolkit composed of cadherin, VWA and CA is evolutionarily maintained and represents part of the basic biomineralization toolkit for skeletal construction. The cadherin domain is a Ca^{2+}-dependent transmembrane glycoprotein and is present in both protocadherin and classical cadherin, which belong to the extracellular matrix-like proteins. One possible role of the cadherin in the coral skeleton formation is to facilitate a connection between the calicoblastic cells and organic matrix within the skeleton [4, 5]. The VWA domain is predominantly associated with proteins involved in cell adhesion and structural support. This domain typically interacts with chitin or fibronectin to form a cross-linked organic matrix network, thereby directing skeletal growth and morphological differentiation [11, 37]. CA is a key enzyme involved in a wide range

of physiological functions and is present in all metazoan clades [38, 39]. The CA in the skeletal proteomes of octocorals and stony corals all possess transmembrane domains or signaling peptides, suggesting that they are the secreted or membrane-associated CAs that can catalyze the interconversion of CO_2 into HCO_3^- in ECM and provide inorganic carbon for $CaCO_3$ precipitation.

We identified large amount of collagen in the octocoral skeletons by the skeletal proteomes analysis and collagen fiber staining. Abundant collagen-like proteins have also been found in the precious red coral *Corallium rubrum* and the gorgonian coral with calcite skeleton [40, 41]. The presence of abundant collagen in the skeletal organic matrix space is likely a striking feature of calcite-forming octocorals. Previous studies have indicated that in the process of skeleton formation, the initial collagen triple helix structure has negatively charged carboxyl groups on the outer side that can combine with calcium ions to assembled into mineralized collagen fibers, which can provide a template for mineral deposition and promote the $CaCO_3$ nucleation [42, 43]. Therefore, collagen may form the basic structural framework of octocoral skeletons. Furthermore, we observed a high frequency of recombination of collagen domains in the axial skeletons of octocorals, including binding to VWA, laminin G, and WAP domains. The binding of the new domain may result in the acquisition of a novel genetic function for collagen during the deposition of CaCO3 skeletal structures. The VWA and laminin G domains are often present in extracellular matrix proteins and are involved in cell-substrate adhesion and the arrangement of $CaCO_3$ crystals [36, 44]. The presence of these proteins may facilitate the cross-linking of collagen with other non-collagenous proteins to form the core matrix framework. The WAP domain plays a pivotal role in regulating innate immunity, protecting against microbial invasion and promoting mucosal tissue repair [45]. A previous study has demonstrated that proteins involved in innate immune

responses can assist stony corals in combating pathogens that penetrate their skeletons, thereby enhancing their calcification capacity [46]. It is therefore proposed that the binding of the collagen domain to the WAP domain may enhance immunity in a matrix framework environment, thereby promoting the deposition of calcified skeleton in octocorals. The majority of octocorals are passive suspension feeders, and their colonies frequently adopt a clumped, tree-like, or net-like structure oriented towards ocean currents [47]. Under such circumstances, collagen might play an important role in strengthening the skeletal structure and enhancing the flexibility of skeleton to cope with the ocean current situation.

Additional Files

Supplementary Figure. This file contains Figure S1-S7.

Supplementary Table. This file contains Table S1-S12.

Supplementary Table S13-S18. This supplementary table contains the annotation file of expansion/contraction gene families in *P. papillata* and *Chrysogorgia* sp. and skeletal proteome analysis file.

Abbreviations

Stony coral: Scleractinia; Octocoral: Octocorallia; $CaCO_3$: Calcium carbonate; SOMPs: Skeletal organic matrix proteins; TEs: Transposable elements; OGs: Orthologue Groups; VG: Van Gieson staining; SEM: Scanning electron microscope; CA: Carbonic anhydrase; SDS-PAGE: Sodium dodecyl sulphate-polyacrylamide gel electrophoresis; ASM: The acid soluble matrix; AIM: The acid insoluble matrix; BUSCO: Benchmarking universal single-copy orthologs; LINEs: Long

interspersed nuclear elements; LTRs: Long terminal repeats; SINEs: Short interspersed nuclear elements; HMC: high-Mg calcite; VWA: von Willebrand factor type A; WAP: WAP-type (Whey Acidic Protein) 'four-disulfide core'; ECM: Extracellular calcifying medium.

Acknowledgements

We appreciate the crew of R/V Kexue and ROV Faxian and HOV Jiaolong for their assistance on sample and data collection. We thank Dr. Zifeng Zhan, Yang Li, Yu Xu, and Dongsheng Wang for providing assistance in collecting and preserving samples at sea. Thanks to Dr. Yu Xu and Dr. Ting Lv for their assistance in using scanning electron microscopy. Thanks to Oceanographic Data Center, Institute of Oceanology, Chinese Academy of Sciences for providing computing power for comparative genomics analysis.

Author's Contributions

YS Liang and KD Xu conceived and designed the project. YS Liang, JY Li, JY Shi assembled the genome, annotated the genes, and performed bioinformatics analyses. YS Liang conducted Van Gieson (VG) staining experiments of coral skeletons, and JY Shi photographed the staining pictures. JH Wei revised the abstract and introduction and provided important points. XY Zheng analyzed the collagen domain and mapped it. WY He and X Zhang used Raman spectroscopy to identify coral skeletons and $CaCO_3$ crystals. YS Liang interpreted the data and drafted the manuscript, KD Xu revised the manuscript. All authors discussed the results and approved the final version of this manuscript.

Funding

This study was supported by the National Natural Science Foundation of China (No. 41930533), the Strategic Priority Research Program of the Chinese Academy of Sciences (No. XDB42000000), and the Senior User Project of R/V KEXUE of the Chinese Academy of Sciences (No. KEXUE2020GZ02).

Data Availability

The deep-sea octocoral genomes has been deposited with the NCBI under the BioProject numbers: PRJNA999483 (*P. papillata*) and PRJNA999484 (*Chrysogorgia* sp.). The whole-genome sequencing data and the RNA-seq data were deposited with the sequence read archive (SRA) database under accession nos. SRR25705840-SRR25705842 (*P. papillata*) and SRR25705989-SRR25705991 (*Chrysogorgia* sp.). The genome related annotation files can be accessed through Figshare at https://doi.org/10.6084/m9.figshare.23984235. The raw data of proteomic sequencing to the ProteomeXchange database with the project ID IPX0010006000. The specific accessions are provided in the respective Material and Methods sections describing the data and analyses.

Competing interests

All financial and non-financial competing interests must be declared in this section.

References

1. Gilbert PUPA, Bergmann KD, Boekelheide N, et al. Biomineralization: Integrating mechanism and evolutionary history. Sci adv. 2022;8(10):eabl9653. https://doi.org/10.1126/sciadv.abl9653.

2. Tambutté E, Allemand D, Zoccola D, et al. Observations of the tissue-skeleton interface in the scleractinian coral *Stylophora pistillata*. Coral Reefs. 2007;26(3):517–29. https://doi.org/10.1007/s00338-007-0263-5.

3. Drake J, Mass T, Haramaty L, et al. Proteomic analysis of skeletal organic matrix from the stony coral *Stylophora pistillata*. Proc Natl Acad Sci USA. 2013;110(10):3788–93. https://doi.org/10.1073/pnas.1301419110.

4. Ramos-Silva P, Kaandorp J, Huisman L, et al. The skeletal proteome of the coral *Acropora millepora*: the evolution of calcification by co-option and domain shuffling. Mol Biol Evol. 2013;30(9):2099–112. https://doi.org/10.1093/molbev/mst109.

5. Takeuchi T, Yamada L, Shinzato C, et al. Stepwise evolution of coral biomineralization revealed with genome-wide proteomics and transcriptomics. PLoS One. 2016;11(6):e0156424. https://doi.org/10.1371/journal.pone.0156424.

6. Conci N, Vargas S, Wrheide G. The biology and evolution of calcite and aragonite mineralization in Octocorallia. Front Ecol Evol. 2021;9:623774. https://doi.org/10.3389/fevo.2021.623774.

7. McFadden CS, Quattrini AM, Brugler MR, et al. Phylogenomics, origin, and diversification of Anthozoans (Phylum Cnidaria). Syst Biol. 2021;70(4):635–47. https://doi.org/10.1093/sysbio/syaa103.

8. Tambutté S, Holcomb M, Ferrier-Pagès C, et al. Coral biomineralization: From the gene to the environment. J Exp Mar Biol Ecol. 2011;408:58–78. https://doi.org/10.1016/j.jembe.2011.07.026.

9. Drake JL, Mass T, Stolarski J, et al. How corals made rocks through the ages. Global Change Biol. 2020;26(1):31–53. https://doi.org/10.1111/gcb.14912.

10. Wang X, Zoccola D, Liew YJ, et al. The evolution of calcification in reef-building corals. Mol Biol Evol. 2021;38(9):3543–55. https://doi.org/ 10.1093/molbev/msab103.

11. Falini G, Fermani S, Gofredo S. Coral biomineralization: a focus on intra-skeletal organic matrix and calcifcation. Semin Cell Dev Biol. 2015;46:17–26. https://doi.org/10.1016/j.semcdb.2015.09.005.

12. Marie B, Joubert C, Tayalé A, et al. Different secretory repertoires control the biomineralization processes of prism and nacre deposition of the pearl oyster shell. Proc Natl Acad Sci USA. 2012;109(51):20986–91. https://doi.org/10.1073/pnas.1210552109.

13. Vurture GW, Sedlazeck FJ, Nattestad M, et al. GenomeScope: fast reference-free genome profiling from short reads. Bioinformatics. 2017;33(14):2202–04. https://doi.org/10.1093/bioinformatics/btx153.

14. Luo RB, Liu BH, Xie YL, et al. SOAPdenovo2: an empirically improved memory-efficient short-read de novo assembler. GigaScience. 2012;1(1):1–18. https://doi.org/10.1186/2047-217X-1-18.

15. Koren S, Walenz BP, Berlin K, et al. Canu: Scalable and accurate long-read assembly via adaptive k-mer weighting and repeat separation. Genome Res. 2017;27(5):722–36. https://doi.org/10.1101/gr.215087.116.

16. Walker BJ, Abeel T, Shea T, et al. Pilon: an integrated tool for comprehensive microbial variant detection and genome assembly improvement. PLoS One. 2014;9(11):e112963. https://doi.org/10.1371/journal.pone.0112963.

17. Guan D, Mccarthy SA, Wood J, et al. Identifying and removing haplotypic duplication in primary genome assemblies. Bioinformatics. 2020;36(9):2896–98.

https://doi.org/10.1093/bioinformatics/btaa025.

18. Simão FA, Waterhouse RM, Ioannidis P, et al. BUSCO: assessing genome assembly and annotation completeness with single-copy orthologs. Bioinformatics. 2015;31(19):3210–12. https://doi.org/10.1093/bioinformatics/btv351.

19. Pertea M, Pertea GM, Antonescu CM, et al. StringTie enables improved reconstruction of a transcriptome from RNA-seq reads. Nat Biotechnol. 2015;33(3):290–95. https://doi.org/10.1038/nbt.3122.

20. Keilwagen J, Wenk M, Erickson JL, et al. Using intron position conservation for homology-based gene prediction. Nucleic Acids Res. 2016;44(9):e89. https://doi.org/10.1093/nar/gkw092.

21. Kim D, Langmead B, Salzberg SL. 2015. HISAT: a fast spliced aligner with low memory requirements. Nat Methods. 2015;12(4):357–60. https://doi.org/10.1038/nmeth.3317.

22. Tang S, Lomsadze A, Borodovsky M. Identification of protein coding regions in RNA transcripts. Nucleic Acids Res. 2015;43(12):e78. https://doi.org/10.1093/nar/gkv227.

23. Haas BJ, Delcher AL, Mount SM, et al. Improving the Arabidopsis genome annotation using maximal transcript alignment assemblies. Nucleic Acids Res. 2003;31(19):5654–66. https://doi.org/10.1093/nar/gkg770.

24. Haas BJ, Salzberg SL, Zhu W, et al. Automated eukaryotic gene structure annotation using EVidenceModeler and the program to assemble spliced alignments. Genome Biol. 2008;9(1):R7. https://doi.org/10.1186/gb-2008-9-1-r7.

25. Flynn JM, Hubley R, Goubert C, et al. RepeatModeler2 for automated genomic discovery of transposable element families. Proc Natl Acad Sci USA. 2020;117(17):9451–57.

https://doi.org/10.1073/pnas.1921046117.

26. Ou S, Jiang N. LTR_retriever: A highly accurate and densitive program for identification of long terminal repeat retrotransposons. Plant Physiol. 2018;176(2):1410–22. https://doi.org/10.1104/pp.17.01310.

27. Tarailo-Graovac M, Chen N. Using RepeatMasker to identify repetitive elements in genomic sequences. Curr Protocols BioInf. 2009;25:4–10. https://doi.org/10.1002/0471250953.bi0410s25.

28. Emms DM, Kelly S. OrthoFinder: solving fundamental biases in whole genome comparisons dramatically improves orthogroup inference accuracy. Genome Biol. 2015;16:157. https://doi.org/10.1186/s13059-015-0721-2.

29. Nguyen LT, Schmidt HA, von Haeseler A, et al. IQ-TREE: A fast and effective stochastic algorithm for estimating maximum-likelihood phylogenies. Mol Biol Evol. 2015;32(1):268–74. https://doi.org/10.1093/molbev/msu300.

30. Yang Z. PAML 4: phylogenetic analysis by maximum likelihood. Mol Biol Evol. 2007;24(8):1586–91. https://doi.org/10.1093/molbev/msm088.

31. De Bie T, Cristianini N, Demuth JP, et al. CAFE: a computational tool for the study of gene family evolution. Bioinformatics. 2006;22(10):1269–71. https://doi.org/10.1093/bioinformatics/btl097.

32. Cox J, Mann M. MaxQuant enables high peptide identification rates, individualized p.p.b.-range mass accuracies and proteome-wide protein quantification. Nat Biotechnol. 2008;26(12):1367–72. https://doi.org/10.1038/nbt.1511.

33. McFadden CS, van Ofwegen LP, Quattrini AM. Revisionary systematics of Octocorallia (Cnidaria: Anthozoa) guided by phylogenomics. Bull Syst Biol. 2022;1(3):8735.

https://doi.org/10.18061/bssb.v1i3.8735.

34. Quattrini AM, Rodríguez E, Faircloth BC, et al. Palaeoclimate ocean conditions shaped the evolution of corals and their skeletons through deep time. Nat Ecol Evol. 2020;4(11):1531–38. https://doi.org/10.1038/s41559-020-01291-1.

35. Yuyama I, Higuchi T. Differential gene expression in skeletal organic matrix proteins of scleractinian corals associated with mixed aragonite/calcite skeletons under low mMg/Ca conditions. PeerJ. 2019;7:e7241. https://doi.org/10.7717/peerj.7241.

36. Rahman MA, Oomori T, Wörheide G. 2011. Calcite formation in soft coral sclerites is determined by a single reactive extracellular protein. J Biol Chem. 2011;286(36):31638–49. https://doi.org/ 10.1074/jbc.M109.070185.

37. Du X, Fan G, Jiao Y, et al. The pearl oyster *Pinctada fucata martensii* genome and multi-omic analyses provide insights into biomineralization. GigaScience. 2017;6(8):1–12. https://doi.org/ 10.1093/gigascience/gix059.

38. Jackson DJ, Macis L, Reitner J, et al. Sponge paleogenomics reveals an ancient role for carbonic anhydrase in skeletogenesis. Science. 2007;316(5833):1893–95. https://doi.org/10.1126/science.1141560.

39. Le Roy N, Jackson DJ, Marie B, et al. The evolution of metazoan α-carbonic anhydrases and their roles in calcium carbonate biomineralization. Front Zool. 2014;11(1):1–16. https://doi.org/10.1186/s12983-014-0075-8.

40. Le Roy N, Ganot P, Aranda M, et al. The skeletome of the red coral *Corallium rubrum* indicates an independent evolution of biomineralization process in octocorals. BMC Ecol Evol. 2021;21:1. https://doi.org/10.1186/s12862-020-01734-0.

41. Goldberg WM. Evidence of a sclerotized collagen from the skeleton of a gorgonian coral. Comp Biochem Phys B. 1974;49(3): 525–26. https://doi.org/10.1016/0305-0491(74)90188-6.
42. Cui FZ, Li Y, Ge J. Self-assembly of mineralized collagen composites. Mater Sci Eng R. 2007;57(1):1–27. https://doi.org/10.1016/j.mser.2007.04.001.
43. Silver FH, Landis WJ. Deposition of apatite in mineralizing vertebrate extracellular matrices: a model of possible nucleation sites on type I collagen. Connect Tissue Res. 2011;52(3):242–54. https://doi.org/10.3109/03008207.2010.551567.
44. Whittaker CA, Hynes RO. Distribution and evolution of von Willebrand/integrin A domains: widely dispersed domains with roles in cell adhesion and elsewhere. Mol Biol Cell. 2002;13(10): 3369–87. https://doi.org/10.1091/mbc.E02-05-0259.
45. Bingle C, Vyakarnam A. Novel innate immune functions of the whey acidic protein family. Trends Immunol. 2008;29(9): 444–53. https://doi.org/10.1016/j.it.2008.07.001.
46. Levy S, Mass T. The skeleton and biomineralization mechanism as part of the innate immune system of stony corals. Fronti Immunol. 2022;13. https://doi.org/10.3389/fimmu.2022.850338.
47. Patterson MR. Passive suspension feeding by an octocoral in plankton patches: Empirical test of a mathematical model. Biol Bull. 1991;180(1):81–92. https://doi.org/10.2307/1542431.

Figure captions

Figure 1: Evolution of the *P. papillata* and *Chrysogorgia* sp. genome. (A and B) The freshly collected specimens of *P. papillata* (A) and *Chrysogorgia* sp. (B). Scale bars=10 cm (a). **(C)** Proportions of DNA transposons, LTR, LINE and SINE retrotransposons in the genomes of six representative anthozoans including *P. papillata* (Ppap), *Chrysogorgia* sp. (Csp), *D. gigantea*

(Dgig), *P. clavata* (Pcla), *Trachythela* sp. (Tsp) and *A. digitifera* (Adig). The tree delineates the evolutionary relationships among the six corals. The pie charts are scaled according to genome size (Supplementary Table S9). **(D)** A phylogenetic tree was constructed with 275 single-copy orthologues from 19 anthozoans and *Hydra vulgaris* (outgroup). Divergence time was estimated with the approximate likelihood calculation method in conjunction with a correlated rates molecular clock. The 95% confidence interval of the estimated divergence time was denoted as blue bar. The positive and negative numbers adjacent to the species abbreviations are gene family numbers of expansion/contraction obtained from the CAFE analysis. Species abbreviations in Supplementary Table S1. Geological era abbreviations: To, Tonian; Cr, Cryogenian; Ed, Ediacaran; Cm, Cambrian; O, Ordovician; S, Silurian; D, Devonian; C, Carboniferous; P, Permian; T, Triassic; J, Jurassic; K, Cretaceous; P, Palaeogene; N, Neogene.

Figure 2: Venn diagram of the protein-domains identified from the four coral SOMPs. SEM images represent the skeletal morphology of these four corals. Domains in bright green are related to the structural support of the skeleton. Domains in blue are mainly involved in cell adhesion. Immunity-related domains are represented in red.

Figure 3: Structural domain and distribution of collagen in axial skeleton of *P. papillata* and *Chrysogorgia* sp. (A) Schematic representation of 5 and 7 collagen proteins identified in the proteome of *P. papillata* and *Chrysogorgia* sp. respectively. **(B)** and **(C)** van Gieson staining results of axial skeleton of *P. papillata* (B) and *Chrysogorgia* sp. (C). The areas of axial skeleton

that contain collagen fibers are stained dark red. For *P. papillata*, the wart-like branching structures are sclerites with collagen fibers distributed on it.

Figure 1

Click here to access/download;Figure;Figure1.pdf

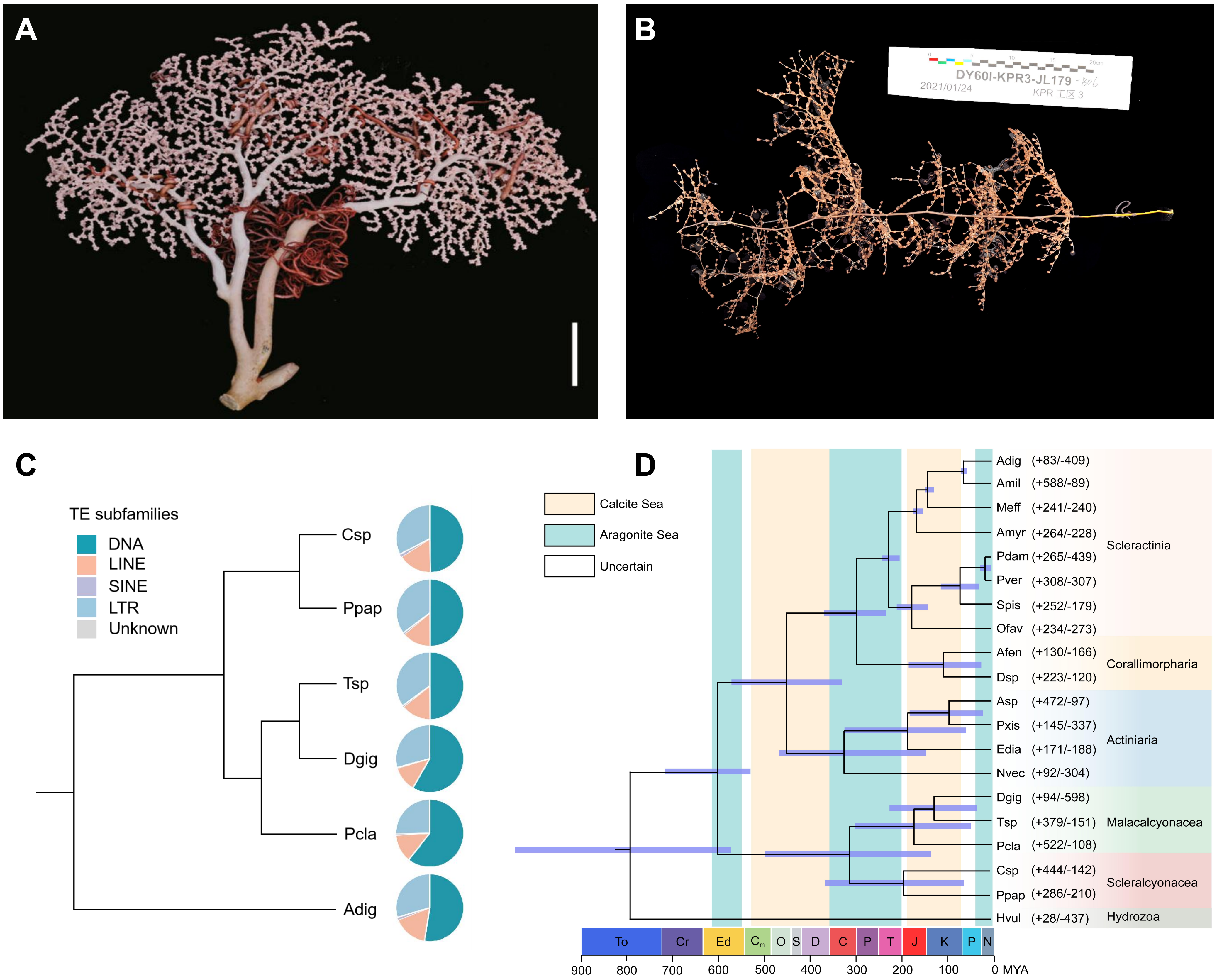

Figure 2

Click here to access/download;Figure;Figure2.pdf

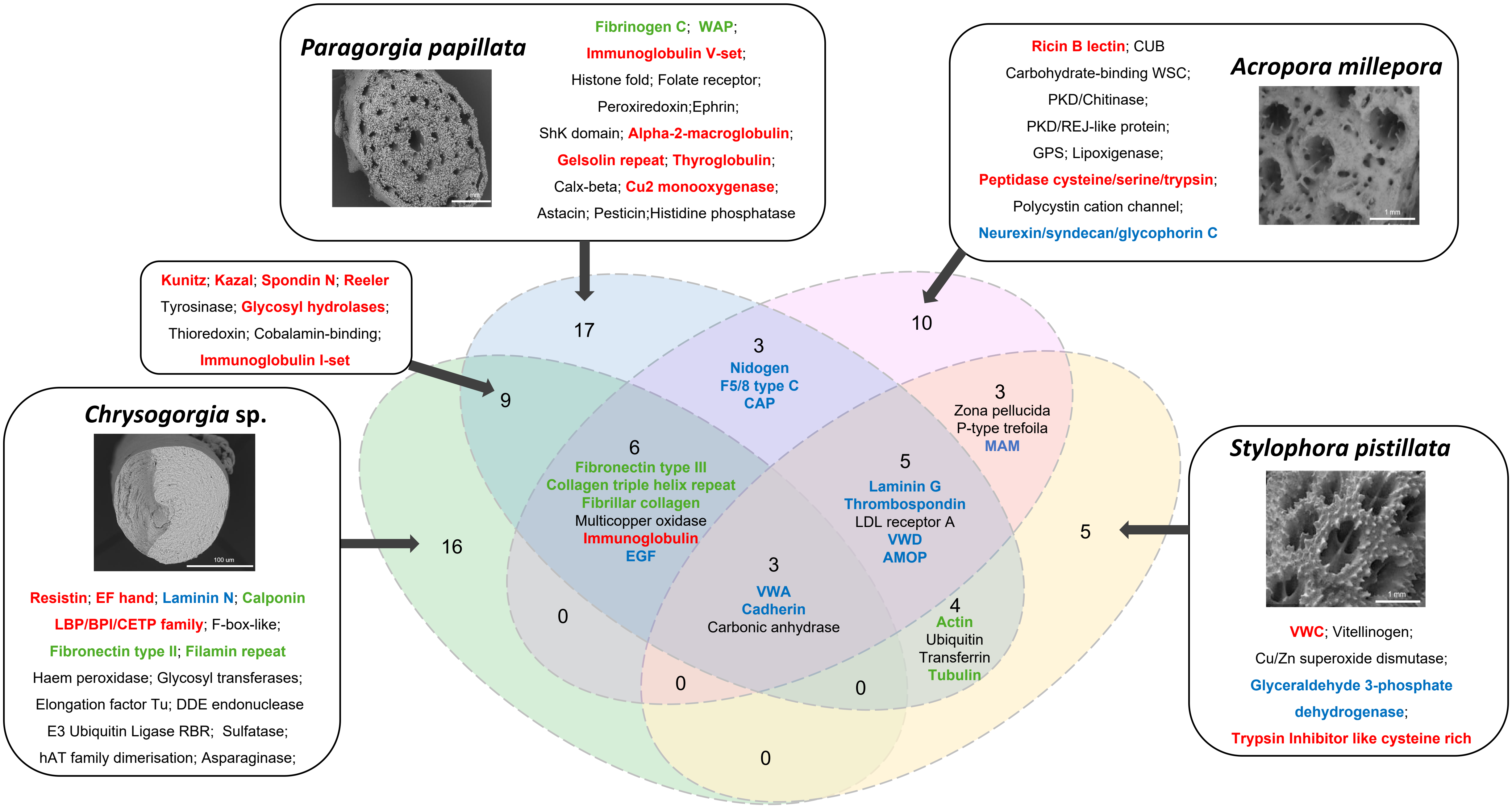

Figure 3

Click here to access/download;Figure;Figure3.pdf

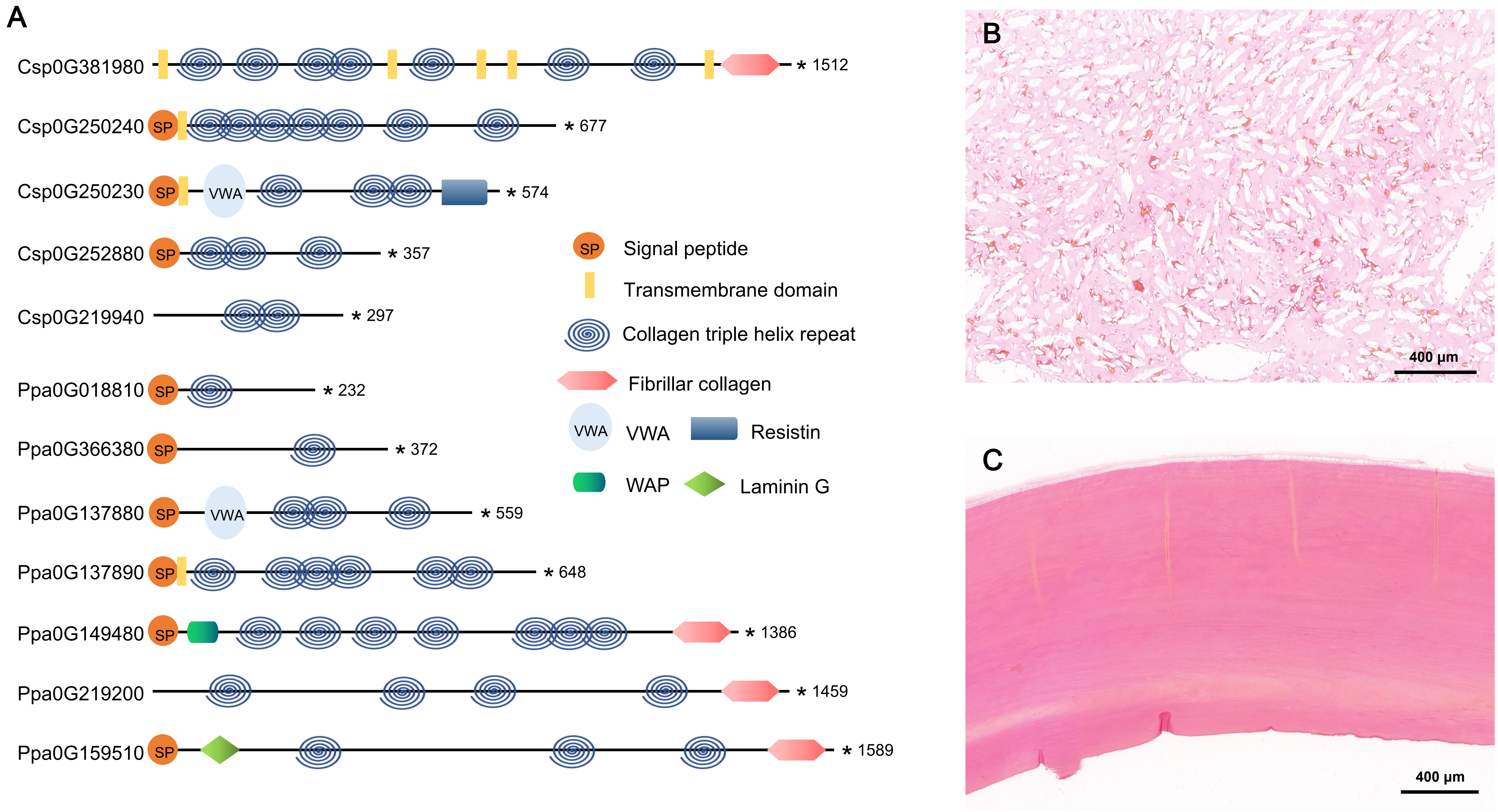

Supplementary Figure

Click here to access/download
Supplementary Material
Supplementary Figure.docx

Supplementary Table

Click here to access/download
Supplementary Material
Supplementary Table.docx

Supplementary Table S13-S18

Click here to access/download
Supplementary Material
Supplementary TableS13-S18.xlsx

Personal Cover Click here to access/download;Personal Cover;Cover letter.docx

Dear Editor:

Thank you very much for inviting reviewers to review our manuscript. All comments and suggestions play an important role in the correction and improvement of this article. Considering that previous submission (**GIGA-D-24-00426: Octocoral skeletal calcification strategies revealed by genome and skeletal proteome analyses**) did not follow the format of 'Data Note', we significantly restructured and reformatted the manuscript to meet the journal's guidelines.

Major changes include the following three areas:

- The title of the manuscript was changed to '**The molecular basis of octocoral calcification revealed by genome and skeletal proteome analyses**'.
- Considering that the manuscript is in the form of a **Data Note** type submission, we removed content related to calcification gene family analyses, PSMC analyses, evolutionary rate analyses, and in vitro crystallization experiment. We focused on the genomic and skeletal proteomic data themselves, performing preliminary comparative genomic and skeletal proteomic analyses.
- In response to the suggestions made by the reviewers, we have made careful revisions in the manuscript.

Given the extensive amendments made to the manuscript, we commenced by reiterating the significance and value of the manuscript to the editors in order to meet the criteria for publication in ***GigaScience***:

The diverse calcification strategies of octocorals are ideal materials for studying biomineralization. However, the lack of genome and proteome in octocorals has limited our understanding of the molecular mechanisms underlying the formation of different $CaCO_3$ polymorphs skeletal structures. In this study, we generated draft genomes of two calcite-forming octocorals (*Paragorgia papillata* and *Chrysogorgia* sp.) and characterized the skeletal proteomes of octocorals. The results include: (1) An overview of the indicators of *P. papillata* and *Chrysogorgia* sp. genome assembly and annotation; (2) Molecular evolutionary features of *P. papillata* and *Chrysogorgia* sp. revealed by phylogenetic analyses and gene family contraction expansion analyses; (3) A comparative analysis of skeletal proteomes has identified the

biomineralization toolkit for skeletal calcification and elucidated the evolution of collagen domains. The availability of these genomic and proteome information provides a valuable resource for understanding the molecular mechanism of coral skeletal formation and its evolutionary history.

We are confident that the publication of our dataset in ***GigaScience*** will contribute to the open scientific discourse and facilitate further studies on coral biomineralization. Given the journal's commitment to publishing high-value datasets, we believe our work is a valuable addition.

Please find the manuscript and associated files attached for your review. We are eager to make any necessary revisions to meet the journal's standards and look forward to your feedback. Thank you for considering our work for publication.

We look forward to hearing from you at your earliest convenience.

Sincerely,

Kuidong Xu (Corresponding authors)

Institute of Oceanology, Chinese Academy of Sciences (No.7 Nanhai Road, Qingdao, 266071, China)

Tel/Fax: 86-532-82898776 Email: kxu@qdio.ac.cn

Response to reviews

To Reviewer #1:

Question 1: First I think it would increase interest in their paper if figure 1 was a photo of the two species and their skeletons as well as a description of their calcified structures. Do they have calcified spicules as well as an axial skeleton and if so where are these located? This information is important for reader understanding, as these genera of octocorals will not be familiar to many readers. So, I suggest moving the photos from Supplementary Fig 2 into the paper itself.

Response: As suggested, we described the axial skeletal structure of two octocorals in Line 555-560, and in order to make readers better understand these two octocorals, we moved Supplementary Fig 2 to Figure 1 in the main text.

Question 2: On a related note, the authors are very inconsistent in their use of genus names, sometimes spelling them out and sometimes abbreviating them. The traditional solution to this problem is to spell out genus and species in full on first use (e.g Acropora millepora) and thereafter use an abbreviation (e.g. A. millepora). While this is a good solution for widely studied Scleractinia, with which readers will be familiar, it is less satisfactory for some of the less familiar genera referred to in this paper, so the best solution that I can think of is to have a table of species names and abbreviations early in the body of the paper. This could be a subset of the information in Table S1. While we are on the topic of names, the Hydra species referred to frequently in the paper is Hydra vulgaris not Hydra vulgatis.

Response: Thanks for this comment. We have added the abbreviations in lines 433-441 of the manuscript and changed the Latin name of *Hydra vulgatis* to *Hydra vulgaris.*

Question 3: There are many minor lapses in English usage scattered throughout the paper. The most common ones are the lack of the articles "a" and "the" and the use of singular where plural should be used. For example, "aragonitic sea" should read "aragonitic seas" and "calcite sea"

should be "calcite seas". So, the paper needs careful English editing.

Response: Thanks for this comment. We have carefully checked and corrected the errors in English usage.

Question 4: Line 23 vaterite is another possible polymorph (e.g. Laipnik et al 2020).

Response:Thanks for this comment. Here we highlight the presence of aragonitic or calcite crystalline skeletal structure in octocorals, and there is no evidence that octocorals have the crystal structure of vaterite. Laipnik et al (2020) emphasize that in vitro crystallization experiments with CARP3 may have facilitated the formation of vaterite. No vaterite skeletal structure has been reported in octocorals.

Question 5: Line 30 "show that it can induce"-"it" must be changed to "they" since the antecedent is CARPs.

Response: Thank you. Due to the major reorganization and reformatting of the manuscript, we have removed the in vitro crystallization experiments.

Question 6: Line 43 according to my reading of the Gilbert paper cited, it would be wise to insert the words "at least" before 541 Myr.

Response: Thanks for this valuable comment. We have made changes in the manuscript.

Question 7: Line 220-229 This region of the paper needs to be converted into English sentences.

Response: Thank you. Due to the major reorganization and reformatting of the manuscript, the content of this paragraph appears in lines 207-213 of the manuscript, and we reworded the content as follows:

The specific procedures were as follows: Firstly, the decalcified axial skeleton was embedded

in paraffin, dewaxed with xylene and ethanol, and stored in tap water. Secondly, the samples were stained with VG staining solution (Servicebio) for 1 min, rinsed rapidly with water, and dehydrated rapidly in anhydrous ethanol triple. Finally, the slides were immersed in xylene until transparent and then coverslipped with neutral resin, observed under a microscope and photographed.

Question 8: Line 390-392 the authors found higher evolutionary rates in their deep sea species.This is not what would be expected, given the relatively stable conditions in the deep sea. Do the authors have any explanation for this?

Response: Thank you. Due to the major reorganization and reformatting of the manuscript, we have removed the evolutionary rate analyses.

Question 9: Line 469-478 The following paper has a good discussion of the relation between biomineralization and immunity and probably deserves a mention

Levy, S. and Mass, T., 2022. The skeleton and biomineralization mechanism as part of the innate immune system of stony corals. Frontiers in Immunology, 13, p.850338.

Response: Thanks for this valuable comment. This paper is very helpful to the discussion of our results and has been cited in the manuscript.

Question 10: Line 555-560 I was surprised to find the following statement at the end of the paper, given what has gone before: "the involvement of CARPs is merely an innovation in the skeletal evolution of aragonitic corals. ". It seems to me that a stronger statement to end on would be the last statement of the previous paragraph: "Thus, the results strongly suggest that CARPs play an important role in the transformation of CaCO3 polymorphs in coral skeletons, which is of great significance for revealing the mechanism of coral biomineralization." The message that I took away from this paper and others is that there are several factors determining what form CaCO3 takes in coral skeletons.

Response: Thank you. Due to the major reorganization and reformatting of the manuscript, we

have removed the discussion related to in vitro crystallization experiments.

Question 11: Nevertheless this paper is important for pointing out the prevalence of collagen in ocgtocoral skeletons.

There are a few papers that have not been mentioned but probably should be, as noted below:

* an early paper pointing out the significance of collagen in the skeleton of a gorgonian

Goldberg, W.M., 1974. Evidence of a sclerotized collagen from the skeleton of a gorgonian coral. Comparative Biochemistry and Physiology Part B: Comparative Biochemistry, 49(3), pp.525-526.

Response: Thanks for this valuable comment. This paper is of great help to the discussion of the role of collagen in the octocoral skeleton and has been cited in the manuscript.

Question 12: *the following paper showed that changes in the molar Mg/Ca ratio (mMg/Ca) in seawater can determine whether aragonite of calcite is laid down and galaxin may also affect the result.

Yuyama, I. and Higuchi, T., 2019. Differential gene expression in skeletal organic matrix proteins of scleractinian corals associated with mixed aragonite/calcite skeletons under low mMg/Ca conditions. PeerJ, 7, p.e7241.

Response: Thanks for this valuable comment. This paper is very helpful to the discussion of our results and has been cited in the manuscript.

To Reviewer #2:

Question 1: 1. Novelty of Findings

While the study presents extensive data, many conclusions primarily confirm previous findings:

Conserved Calcification Gene Families: The idea that calcification gene families are shared across corals and even other animal lineages was suggested in previous studies (Drake et al 2014, Conci et al, 2021, Wang et al. 2021,). This finding is not novel.

CARP and Collagen Roles: Previous research has demonstrated that calcite-forming octocorals lack CARPs and utilize collagen in their skeletal matrix (e.g., Conci et al. 2020; Le Roy et al. 2021). These results are not unique to this study.

CARPs and Polymorph Transformation: The authors' experiments with CARP4 suggest its role in calcite-to-aragonite transformation. However, prior work on CARP3 (e.g., Laipnik et al. 2020) indicates that CARPs' roles may depend on ion composition, questioning the generalization of CARP functions in polymorph selection.

Response: Thanks for this valuable comment. Considering that the manuscript is in the form of a Data Note type submission, we removed content related to calcification gene family analyses, PSMC analyses, evolutionary rate analyses, and in vitro crystallization experiment.

In the new manuscript, we focus on the assembly, annotation and underlying comparative genomic analyses of two octocorals. Skeletal proteome analyses led us to identify a basic toolkit for coral calcification. We further found that collagen in the skeleton of octocorals has evolved structural domains associated with matrix adhesion and immunity, which may confer new genetic functions for calcification in octocorals. These genomes and proteomes expand the list of octocoral genomes and provide a valuable resource for understanding the molecular mechanisms of coral skeletal formation and their evolutionary history.

Question 2: Methodological Concerns

Genome Quality: The genomes provided do not appear to meet today's standards to call them "high-quality genomes". As such, I would expect chromosome-level assemblies. Nontheless I believe the quality of the genomes suffice to perform the kind of analyses presented here, as they rely on completeness of genes, rather than chromosome level- assemblies. However, also the methods for

excluding microbial contamination from assembled contigs are unclear.

Demographic History Analysis: Estimating changes in effective population size from a single genome raises concerns. How sensitive are the results to assumptions like generation time (set at 35 years based on estimation for unrelated stony corals)? The authors should provide sensitivity analyses to address such assumptions. Additionally, it remains unclear how the analysis contributes to the study because its results are not discussed at all. It may be considered to omit it from the manuscript.

In Vitro Crystallization:

-The rationale for selecting CARP4 (as opposed to other CARPs or combinations) is unclear. What is the physiological relevance of the protein concentration used?

-The recombinant CARP4 used in the experiments lacks post-translational modifications, which could affect its function in vivo, which should be mentioned.

-Details on replication, SEM, and Raman spectroscopy (e.g., instruments used, number of spectra measured) are insufficient.

-The absence of Mg2+ in the experimental setup is concerning, as Mg:Ca ratios are known to influence polymorph selection (Laipnik et al. 2020).

Response: Thanks for this valuable comment. Due to the major reorganization and reformatting of the manuscript, we removed content related to the demographic history analysis and in vitro crystallization experiment.

For genome quality, we did not assemble these two octocoral genomes to the chromosome level. However, from the length of contig N50 and the complete BUSCO genes, the assembly and annotation quality of these two octocoral genomes is higher than that of the published octocoral genomes. As suggested, we modified the wording from ‘high-quality genomes’ to ‘draft genomes’.

For the elimination of microbial reads contamination, we have added in lines 122-124 of the manuscript, as follows:

Contaminated reads containing chloroplast, mitochondrial, bacterial or viral sequences were removed via comparison of the genome assembly with the nucleotide sequence database (nt) from the National Center for Biotechnology Information (NCBI).

Question 3: Introduction

Lines 63-66: The statement, "Information on the proteins associated with coral calcification is extremely limited..." is vague. Provide specific details and cite relevant studies.

Response: Thank you. We have rewritten this section to emphasize that the absence of the octocoral genomes and skeletal proteomes limits our understanding of the molecular mechanisms underlying octocoral skeletal formation, as follows:

However, the lack of genome and proteome in octocorals has limited our understanding of the molecular mechanisms underlying the formation of different $CaCO_3$ polymorphs skeletal structures.

Question 4: Expand on the known role of CARPs in carbonate polymorph selection. How many CARPs are known? Why focus on CARP4 from Stylophora pistillata?

Response: Thank you. According to your suggestion, we have rewritten the manuscript and deleted the experimental content of CARP4 participating in in vitro calcium carbonate crystallization.

Question 5: Methods

Clarify the choice of octocoral species. Were these species selected for specific reasons, or were they chosen based on availability? Because the species, unfortunately, remained unidentified, please clarify if at least morphological voucher material for later species identification is available. Address genome quality concerns. Were microbial symbionts excluded?

Response: To date, the number of published genomes of octocorals is small, and we selected *D. gigantea*, *Trachythela* sp., and *P. clavata* for comparative analyses with the two octocorals we sequenced based on data availability. In addition, *Chrysogorgia* sp. was not identified to species, and identification of this species is ongoing.

For the elimination of microbial reads contamination, we have added in lines 122-124 of the manuscript, as follows:

Contaminated reads containing chloroplast, mitochondrial, bacterial or viral sequences were removed via comparison of the genome assembly with the nucleotide sequence database (nt) from the National Center for Biotechnology Information (NCBI).

Question 6: Justify the selection of CARP4 for in vitro experiments. Discuss the potential effects of lacking post-translational modifications.

Response: Thank you. According to your suggestion, we have rewritten the manuscript and deleted the experimental content of CARP4 participating in in vitro calcium carbonate crystallization.

Question 7: Specify the number of replicates for crystallization experiments and describe SEM and Raman spectroscopy methods in detail.

Response: Thank you. In the new manuscript, we have deleted the content of crystallization experiment. In addition, we used SEM and Raman spectroscopy to analyze the axial skeletal structure characteristics of *P. papillata* and *Chrysogorgia* sp. We believe that we have made it clear in the Materials method section, as follows:

To observe the skeletal ultrastructure, the axial skeletons of *P. papillata* and *Chrysogorgia* sp. were isolated by digestion of the tissues in sodium hypochlorite, and then were washed repeatedly with milli-Q water repeatedly. Then, the axial skeletons were transferred to carbon double-adhesive tape, air-dried and coated for analysis by the scanning electron microscopy (SEM). SEM scans were obtained using a Hitachi TM3030Plus SEM at 15 kV and the optimum magnification for each axial skeleton. To investigate the $CaCO_3$ polymorphs of coral skeletons, we used the confocal Raman spectroscopy technology (Alpha 300R+, WITec, Ulm, Germany) to detect the axial skeleton after the removal of the coenenchyme.

Question 8: Results

Line 413: The hypothesis that CARPs induce calcite-to-aragonite transformation should be introduced in the Introduction, not the Results.

Discuss how the experimental findings align or conflict with prior work on CARP3 (e.g., Laibnik et al. 2020). According to the experiments on CARP3, is ion composition the primary driver of polymorph selection rather than the presence of a CARP?

Response: Thank you. According to your suggestion, we have rewritten the manuscript and deleted the experimental content of CARP4 participating in in vitro calcium carbonate crystallization.

Question 9: Please reconsider the naming conventions for the identified proteins, as they appear to be based solely on the best BLAST hits of an unknown coral protein or on previously identified proteins from earlier studies. For example, the protein "Ppa0G268680" is annotated as "Sushi domain-containing 2-like." However, this annotation seems to derive from a BLAST hit to a protein with only a rudimentary annotation and an unknown function purportedly containing a sushi domain. Notably, "Ppa0G268680" does not actually contain a sushi domain. Instead, InterProScan identifies the presence of a Nidogen-like domain (PF06119), an AMOP domain (PF03782), and a von Willebrand factor type D domain (PF00094)

Response: Thanks to the reviewer for pointing out the error, different databases may not annotate the protein name consistently, but according to the domain features, the annotation of Ppa0G268680 appeared to be an error, we carried out to correct the protein name of Ppa0G268680 in Supplementary Table S15 and Supplementary Table S17 , and changed it to Protein mesh. Other identified proteins were also checked.

Question 10: Discussion

-Lines 532 ff.: The statement "CARPs can induce the transformation of CaCO3 polymorphs" is not novel. Emphasize what new insights this study provides.

Highlight the implications of the findings for understanding coral calcification mechanisms and evolutionary strategies beyond confirming prior work.

Response: Thanks for this valuable comment. Considering that the manuscript is in the form of a Data

Note type submission, we deleted the previous discussion and rewrote it.

In the new manuscript, we focus on the assembly, annotation and underlying comparative genomic analyses of two octocorals. Skeletal proteome analyses led us to identify a basic toolkit for coral calcification. We further found that collagen in the skeleton of octocorals has evolved structural domains associated with matrix adhesion and immunity, which may confer new genetic functions for calcification in octocorals. These genomes and proteomes expand the list of octocoral genomes and provide a valuable resource for understanding the molecular mechanisms of coral skeletal formation and their evolutionary history.

Question 11: Figures and Supplementary Data

-Figure legend Fig 2A: "Distribution of core calcification genes involved in Ca2+ transport and concentration regulation, inorganic carbon transport, catalysis of calcification related enzymes, and regulation of organic matrix proteins in corals of different skeletal types (calcite, aragonite and none)"

—What is shown in the figure is not the number of calcification genes, but the number of hits to specific genes. Many (most) of these genes are not involved in calcification but just belong to the same gene families. This should be clarified.

-Figures S11-S14 lack support values for phylogenies, reducing their reliability. Gene family origins (e.g., PMCAs, SLC4, CAs) predate Anthozoa, meaning the phylogenies do not adequately inform about evolutionary history. Compare these with phylogenies that included sequences of other phyla (e.g., SLC4: Voigt et al. 2017; CA: Voigt et al. 2021).

Response: Thank you. According to your suggestion, we deleted Figure related to calcification gene families and phylogenetic results of PMCAs, SLC4, and CAs.

Question 12: Data Availability

-Lines 625 ff.: Clarify data availability under the BioProject. Please submit the genome assemblies (and mention the accession codes here) and their annotation to NCBI, and NOT only provide the annotation in FigShare.

Response: Thank you. Our data is fully available from assembly to annotation, there is no need to upload annotation files to the NCBI database repeatedly.

Question 13: Please provide the raw spectra of the LS/LS MS in an appropriate repository.

Response: As suggested, we have added the raw spectra in the Data Availability of the manuscript, as shown below:

The raw data of proteomic sequencing to the ProteomeXchange database with the project ID IPX0010006000.
