# Supplementary material for: The molecular basis of octocoral calcification revealed by genome and skeletal proteome analyses
Source: Gigascience. 2025 Apr 1;14:giaf031. doi: 10.1093/gigascience/giaf031 (PMC11959691; doi:10.1093/gigascience/giaf031)
Supplement: giaf031_GIGA-D-24-00546_Revision_1 [file giaf031_giga-d-24-00546_revision_1.pdf]

GigaScience

The molecular basis of octocoral calcification revealed by genome and skeletal proteome analyses

--Manuscript Draft--

**Manuscript Number:**	GIGA-D-24-00546R1
**Full Title:**	The molecular basis of octocoral calcification revealed by genome and skeletal proteome analyses
**Article Type:**	Data Note
**Funding Information:**	National Natural Science Foundation of China (No. 41930533) — Prof. Kuidong Xu<br>Strategic Priority Research Program of the Chinese Academy of Sciencescs, Chinese Academy of Sciences (No. XDB42000000) — Prof. Kuidong Xu<br>Senior User Project of R/V KEXUE of the Chinese Academy of Sciences (No. KEXUE2020GZ02) — Prof. Kuidong Xu
**Abstract:**	The ability of octocorals and stony corals to deposit calcium carbonate (CaCO3) have led to their ecological success. Compared with the homogeneous aragonite skeleton of stony corals, octocorals have evolved different skeletal structures composed of different CaCO3 polymorphs and skeletal organic matrix. However, the molecular basis of skeletal structure formation in octocorals remains largely unexplored. Here, we generated the genomes and skeletal proteomes of two calcite-forming octocorals, Paragorgia papillata and Chrysogorgia sp. Both assembled genomes size is 618.13 Mb and 781.04 Mb for P. papillata and Chrysogorgia sp. respectively, with contig N50 of 2.67 Mb and 2.61 Mb. Comparative genomic analyses identified 162 and 285 significantly expanded gene families in the genomes of P. papillata and Chrysogorgia sp., respectively, which were mainly associated with biomineralization and immune response. Comparative analyses of skeletal proteomes revealed that corals with different CaCO3 polymorphs share a basic toolkit consisting of cadherin, von Willebrand factor type A and carbonic anhydrase domains for calcified skeleton deposition. By contrast, collagen is rich in the calcite-forming octocoral skeletons, but rarely occurs in aragonitic stony corals. Furthermore, some collagens have evolved domains related to matrix adhesion and immunity, which may confer new genetic functions on the calcification in octocorals. These findings facilitate our comprehension of the diverse forms of coral biomineralization and provide preliminary insights into the formation and evolution of the octocoral skeleton.
**Corresponding Author:**	Yanshuo Liang<br>Institute of Oceanology Chinese Academy of Sciences<br>Qingdao, CHINA
**Corresponding Author Secondary Information:**	
**Corresponding Author's Institution:**	Institute of Oceanology Chinese Academy of Sciences
**Corresponding Author's Secondary Institution:**	
**First Author:**	Yanshuo Liang
**First Author Secondary Information:**	
**Order of Authors:**	Yanshuo Liang<br>Kuidong Xu<br>Junyuan Li<br>Jingyuan Shi<br>Jiehong Wei

Powered by Editorial Manager® and ProduXion Manager® from Aries Systems Corporation

	Xiaoyu Zheng
	Wanying He
	Xin Zhang
**Order of Authors Secondary Information:**	
**Response to Reviewers:**	Response to reviews<br>We sincerely thank the editor and all reviewers for their valuable feedback that we have used to improve the quality of our manuscript (The molecular basis of octocoral calcification revealed by genome and skeletal proteome analyses; GIGA-D-24-00546). All comments and suggestions play an important role in the correction and improvement of this article. We have carefully revised the article according to the requirements of the editor and reviewers, and made detailed instructions as shown below.<br><br>To Reviewer #1:<br>Question 1: * I think there should be a clear statement of whether either of these species has calcified spicules in addition to an axial skeleton. From the manuscript it appears that neither species has spicules but because they are common in octocorals this should be clarified.<br>Response: Thanks for this valuable comment. I think your point of view is correct, but I think the spicules you're talking about might be sclerites. In order to give readers a better understanding of these two octocoral morphological characteristics, we have re-explained the calcification structure in the Lines 317-322, as follows:<br>“To determine the types of the octocoral skeletons, we utilized Raman spectroscopy and scanning electron microscopy to analyze the skeletal structures. We found calcified sclerites embedded at both polyps and coenosarc tissues in P. papillata and Chrysogorgia sp. The axial skeleton of P. papillata was found to consist of the accumulation of high-Mg calcite (HMC) sclerites of varying morphologies, forming a ring of regularly arranged central pores. In contrast, the axial skeleton of Chrysogorgia sp. is a fully calcified, HMC structure with a growth pattern analogous to that of annual rings (Fig. 2; Supplementary Fig. S6).”<br><br>Question 2: * the same sorts of grammatical mistakes that I pointed out previously are still present.<br>There are many instances where the articles "the" and "a" are needed and where singular and plural are incorrectly used. For example, in the first sentence of the abstract there is an example of the latter mistake. The word "ability" is singular and determines the verb, which should be "has" not "have".<br>Response: Thanks for your suggestion. We asked a company specializing in English editing to polish the manuscript.<br><br>Question 3: * to repeat my previous comment: "On a related note, the authors are very inconsistent in their use of genus names, sometimes spelling them out and sometimes abbreviating them. The traditional solution to this problem is to spell out genus and species in full on first use (e.g Acropora millepora) and thereafter use an abbreviation (e.g. A. millepora)."yet on lines 153-155 the authors still have" A.digitifera, A. millepora, Astreopora myriophthalma, D. gigantea, Porites australiensis, P. clavata,Stylophora postillata." Is there a reason that they don't want to follow the usual convention?<br>Response: We were really sorry for our careless mistakes. We have revised it in the manuscript according to your suggestion.<br><br>Question 4: *The reply of the authors to question 10 from reviewer 2 is an excellent, succinct summary of their work , which perhaps could be incorporated into their paper. "we focus on the assembly, annotation and underlying comparative genomic analyses of two octocorals. Skeletal proteome analyses led us to identify a basic toolkit for coral calcification. We further found that collagen in the skeleton of octocorals has evolved structural domains associated with matrix adhesion and immunity, which may confer new genetic functions for calcification in octocorals. These genomes and proteomes expand the list of octocoral genomes and provide a valuable resource for understanding the molecular mechanisms of coral skeletal formation and their evolutionary history."<br>Response: Thanks for this valuable comment. We added the Data Description at the end of the discussion section as summary of our work, as shown below:

Powered by Editorial Manager® and ProduXion Manager® from Aries Systems Corporation

Data Description
This study focuses on the assembly, annotation and underlying comparative genomic analyses of two octocoral genomes, P. papillata and Chrysogorgia sp. We further characterized the axial skeletal proteomes of these two octocorals to identify a basic toolkit for coral calcification by comparison with the skeletal proteomes of aragonitic corals. We also found that collagen in the axial skeleton of octocorals has evolved structural domains associated with matrix adhesion and immunity, which may confer new genetic functions for calcification in octocorals. These genomes and proteomes expand the list of octocoral genomes and provide a valuable resource for understanding the molecular mechanisms of coral skeletal formation and their evolutionary history.

Question 5: * Suppl Table S1 is a very useful addition to the paper
Response: Thank you.

Question 6: * I was unable to view Tables S13-S18. Following S12 there is a page with the following:
Table S13-S18 in the Supplementary information section
Response: We put TableS13-S18 in a table execl for Supplementary TableS13-S18.xlsx. This file has been successfully uploaded in the first submission. In order to avoid not seeing it again, I will upload it again when I submit the revision draft.

Question 7: * The captions to the Supplementary figures could use English editing
Response: Thanks for your suggestion. We have tried our best to polish the language in the captions of the Supplementary figures.

To Reviewer #2:
Question 1: Line 20: "have led to their" to "has led to their"
Response: We sincerely thank the reviewer #2 for careful reading. As suggested, we have corrected the "have led to their" to "has led to their"

Question 2: Lines 25-27: "Both assembled genomes size is 618.13 Mb and 781.04 Mb for P. papillata and Chrysogorgia sp. respectively, with contig N50 of 2.67 Mb and 2.61 Mb" to "The assembled genomes sizes were 618.13 Mb and 781.04 Mb for P. papillata and Chrysogorgia sp., respectively, with contig N50s of 2.67 Mb and 2.61 Mb"
Response: Thank you. According to your suggestion, we have made changes in the manuscript.

Question 3: Line 43: Is this what you were trying to say? "Biomineralization of CaCO3 originated in organisms at least 541 Mya"
Response: Thank you. Here we would like to state that the history of biological CaCO3 mineralization has lasted at least 541 Mya. The earliest known CaCO3 skeletons are problematic metazoans found in late Ediacaran (550 to 541 Ma before present) rocks distributed globally (Wood et al, 2002; Gilbert et al, 2022). In order not to mislead the reader, we have rephrased the manuscript as follows:
“The history of CaCO3 biomineralization by organisms spans at least 541 Myr, and biomineralization as an innovative mechanism in the evolutionary history of life has played a significant role in species development and global carbon cycles [1]”

Reference:
Gilbert PUPA, Bergmann KD, Boekelheide N, et al. Biomineralization: Integrating mechanism and evolutionary history. Sci adv. 2022;8(10):eabl9653.
Wood RA, Grotzinger JP, Dickson J. Proterozoic modular biomineralized metazoan from the Nama Group, Namibia. Science. 2002;296(5577):2383-2386.

Question 4: Line 46: "biogenic structures by their ability" to "biogenic structures through their ability"
Response: As suggested, we have made changes in the manuscript.

Question 5: Line 48: "CaCO3 skeleton-producing ability is found" to "The ability to produce CaCO3 skeletons is found"
Response: According to your suggestion, we have made revisions in the manuscript.

Powered by Editorial Manager® and ProduXion Manager® from Aries Systems Corporation

Question 6: Line 58: "of diverse organic matrix to calcification site" to "of a diverse organic matrix at the site of calcification"
Response: According to your suggestion, we have made revisions in the manuscript.

Question 7: Line 59: "Major components of organic matrix include proteins" to "Major components of the organic matrix include proteins"
Response: As suggested, we have made changes in the manuscript.

Question 8: Line 103-104: "This was followed by extending, gap filling and polishing the assembly" -> What tools and parameters were used for this? Was it SOAPdenovo or something else? Please elaborate.
Response: Thanks for this comment. Here we did not use specialized software to extend, fill gap and polish the genome, but through SOAPdenovo software to assemble the Illumina paired-end reads and set parameters to achieve these steps (the parameter: -K 45 -d 1 -D 1 -F). We have rephrased it in the manuscript as follows:
"The trimmed Illumina paired-end reads were assembled into scaffolds using SOAPdenovo v2.04 (RRID:SCR_010752) [14] with the following specified parameters: -K 45 -d 1 -D 1 -F."

Question 9: Line 120: "Following the removal of low-quality and duplicate reads" -> Using what tools and parameters? Please elaborate.
Response: Thank you. According to your suggestion, we have rephrased it in the manuscript as follows:
"PacBio long reads were subjected to quality controlled by using SequelQC software (RRID:SCR_017279), and the clean reads were corrected using the error correction module of Canu v.1.5 (RRID:SCR_015880) [15] to select longer subreads."

Question 10: Line 123: "sequences were removed via comparison of the genome assembly" -> Using what tools and parameters? I assume BLASTN, but using what cutoffs? Please elaborate.
Response: As suggested, we have rephrased it in the manuscript as follows:
"Contaminated reads containing chloroplast, mitochondrial, bacterial or viral sequences were removed through comparison of the genome assembly with the nucleotide sequence database from the National Center for Biotechnology Information (NCBI) using BLASTN v.2.2.26 with an e-value threshold of ≤ 1e−5."

Question 11: Line 128: "Duplicated genes in" -> Do you mean duplicated haplotigs? Purge_dups operates on scaffolds/contigs, not genes.
Response: Thanks for your careful checks. Based on your comments, we have made the corrections in the manuscript as follows:
"Haplotigs and contig overlaps in the resulting assembly were eliminated using Purge_dups v.1.2.5 (RRID:SCR_021173)."

Question 12: Line 134: "orthologues to the genome by using BUSCO v.5.0" to "orthologues to the genome using BUSCO v5.0"
Response: As suggested, we have made changes in the manuscript.

Question 13: Line 153: "gene model from the other cnidarians" to "gene model from a selection of other cnidarians" -> You are not using all cnidarian genome available, there for you need to make it clear throughout the manuscript for each of your analyses, that you are using a small subset of the genome data available.
Response: Thank you. According to your suggestion, we have rephrased it in the manuscript.

Question 14: Line 159: "combined using the EVM v1.1.1" to "combined using EVM v1.1.1" -> what weights did you use for EVM. Any additional parameters. Please elaborate.
Response: According to the suggestion, we made changes in the manuscript and added the weights of EVM, as follows:
"The weights assigned to ab initio prediction, protein alignment, and transcript were 4, 7, and 8, respectively."

Question 15: Line 177: "results were used to assign the OGs by OrthoFinder v2.4.0" to "results were used by OrthoFinder v2.4.0 to construct OGs"

Powered by Editorial Manager® and ProduXion Manager® from Aries Systems Corporation

Response: As suggested, we have made changes in the manuscript.

Question 16: Line 187: Why were OGs with > 100 copies excluded. Please justify this cutoff.
Response: Thanks for this comment. Excluding the orthologue groups greater than 100 copies during gene family contraction and expansion analysis is a common practice to ensure the accuracy, reliability, and biological relevance of the results. Here are the key reasons for this exclusion:
Some OGs may undergo extensive species-specific gene duplication events (e.g., tandem duplications or whole-genome duplications), leading to an unusually high number of copies in a single species.
Excluding high-copy gene families helps focus the analysis on orthologs, providing a clearer picture of gene family evolution across species.
High-copy gene families may introduce biases in statistical analysis, resulting in distorted results. For example, certain species may exhibit unusually high gene copy numbers for technical reasons (such as differences in the quality of genome assembly), and excluding these gene families can avoid the impact of this technical bias on the overall analysis.

Question 17: Line 236: "sequence similarity protein searches against the NR" -> Provide details on the tools, parameters, and cutoffs used.
Response: According to your suggestion, we have rephrased it in the manuscript as follows:
"Protein annotation was performed by sequence similarity search against the NR database in NCBI and the UniProtKB/SwissProt database using BLASTP with an E-value threshold of 1×10^{-5}."

Question 18: Line 253: "K-mer-based" -> italicize the "K" in k-mer wherever it is used, it should also be lowercase, unless at the start of a sentence.
Response: Thanks for this comment. We have revised all the "K-mer" in the manuscript to "k-mer".

Question 19: Line 255: "assembly are 2.67 Mb, while they are 2.61 Mb" to "The contig N50 of the P. papillata assembly is 2.67 Mb and the Chrysogorgia sp. assembly is 2.61 Mb."
Response: As suggested, we have made changes in the manuscript.

Question 20: Line 262: It would be good to also mention the low number of duplicated BUSCO genes, which supports the high quality of the genomes, and your downstream gene family expansion analysis. Additionally, most genome studies compare their assemblies against those currently available, to demonstrate the quality of their new data. While not strictly required, it would be good to mention the quality of the available octocoral data.
Response: As suggested, we rephrased it in the manuscript as follows:
"BUSCO analysis with the metazoan database showed that the genome assemblies of P. papillata and Chrysogorgia sp. contained 91.61% and 88.36% complete BUSCO genes, respectively. Moreover, the proportion of duplicated BUSCO genes in both genomes was 1.89%, which was comparable to that in previously published octocoral genomes (Supplementary Table S6)."

Question 21: Line 266: "were identified in" to "were predicted in"
Response: As suggested, we have made changes in the manuscript.

Question 22: Line 267: "with 91.01% and 90.06% of genes annotated into Nr" -> I do not believe that "annotated" is the correct word here. Just because it has hits to nr does not mean that it is annotated. It has "homology" to other sequences in nr, but annotation implies known function, and many proteins in nr have unknown function. Hits to KEGG and PFAM would be considered "annotated" since they are function-focused databases. Please rephrase.
Response: Thanks for this valuable comment. We rephrased it in the manuscript as follows:
"Among these PCGs, 37,974 (91.01%) in P. papillata and 47,126 (90.06%) in Chrysogorgia sp. were assigned functional annotations by comparing with public databases, including SwissProt, Pfam, NR, TrEMBL, eggNOG, KOG, KEGG, and GO

Powered by Editorial Manager® and ProduXion Manager® from Aries Systems Corporation

(Supplementary Table S8).”

Question 23: Line 269-270: I would not say that they are "relatively more variable", just more variable than the species that you selected. If you look across all assembled coral genomes, you see a wider variety of sizes (up to 40,000-50,000 genes in some genomes like Montipora capitata or Porites lobata). Consider rephrasing or giving additional context.
Response: Thanks for this valuable comment. Due to our lack of statistics on the protein-coding genes of all published coral genomes, we proposed that such a conclusion was incorrect, so we rephrased it in the manuscript as follows:
“While considerable variation exists in the number of PCGs among different corals, octocoral genomes typically demonstrate higher counts than hexacoral genomes”

Question 24: Line 270-273: "Genome annotation completeness was also evaluated using BUSCO, and the results showed that the ortholog genes of P. papillata and Chrysogorgia sp. contributed 94.23% and 92.87% of complete genes, respectively (Supplementary Table S10), indicating that our gene annotation is highly complete." to "The BUSCO completeness of the predicted genes in each genome was 94.23% for P. papillata and 92.87% for Chrysogorgia sp. (Supplementary Table S10), indicating that our predicted genes are highly complete."
Response: According to your suggestion, we have made revisions in the manuscript.

Question 25: Line 275-280: "Using a combination of homology-based and de novo approaches, the TEs of 294.04 and 374.71 Mb are identified in the P. papillata and Chrysogorgia sp. genomes (47.58% and 47.98%, respectively) (Fig. 1C; Supplementary Table S11 and S12), with both class II DNA transposons predominate (23.76% and 23.84%, respectively), while class I retrotransposons (long interspersed nuclear elements (LINEs), long terminal repeats (LTRs) and short interspersed nuclear elements (SINEs)) account for 23.81% and 24.14% of the genomes, respectively." to "Using a combination of homology-based and de novo approaches, 294.04 Mb and 374.71 Mb (47.58% and 47.98%) of the P. papillata and Chrysogorgia sp. genomes (respectively) were identified as TEs (Fig. 1C; Supplementary Table S11 and S12), with 23.76% and 23.84% of these TEs being class II DNA transposons and 23.81% and 24.14% of these TEs being class I retrotransposons (long interspersed nuclear elements [LINEs], long terminal repeats [LTRs], and short interspersed nuclear elements [SINEs])."
Response: According to your suggestion, we have made revisions in the manuscript.

Question 26: Line 301: "among the available anthozoan genomes" to "among a selection of the available anthozoan genomes"
Response: According to your suggestion, we have made revisions in the manuscript.

Question 27: Line 423: Normally in a "Data Note" manuscript you would have a concluding paragraph about how good your data is compared to the existing published data, and how it can be used to explore the biology of your system. While this is not required, I recommend that the authors add a brief paragraph summarizing the data presented in the manuscript.
Response: Thanks for this valuable comment. We added the Data Description at the end of the discussion section, as shown below:
Data Description
This study focuses on the assembly, annotation and underlying comparative genomic analyses of two octocoral genomes, P. papillata and Chrysogorgia sp. We further characterized the axial skeletal proteomes of these two octocorals to identify a basic toolkit for coral calcification by comparison with the skeletal proteomes of aragonitic corals. We also found that collagen in the axial skeleton of octocorals has evolved structural domains associated with matrix adhesion and immunity, which may confer new genetic functions for calcification in octocorals. These genomes and proteomes expand the list of octocoral genomes and provide a valuable resource for understanding the molecular mechanisms of coral skeletal formation and their evolutionary history.

Question 28: Line 610: "genome" to "genomes".
Response: According to your suggestion, we have made revisions in the manuscript.

Powered by Editorial Manager® and ProduXion Manager® from Aries Systems Corporation

	Question 29: Line 619-620: "the estimated divergence time was denoted as blue bar" to "the estimated divergence time at each node is denoted as a blue bar"<br>Response: According to your suggestion, we have made revisions in the manuscript.<br><br>Question 30: Supplementary Figure S3: Why not show the GenomeScope images? Also, "was as the input file of" to "was used as the input file to".<br>Response: We feel sorry for our carelessness. We performed the survey analysis using Jellyfish software only, not GenomeScope, and we deleted the relevant content in the manuscript and supplementary files.
**Additional Information:**	
**Question**	**Response**
Are you submitting this manuscript to a special series or article collection?	No
**Experimental design and statistics**<br><br>Full details of the experimental design and statistical methods used should be given in the Methods section, as detailed in our Minimum Standards Reporting Checklist. Information essential to interpreting the data presented should be made available in the figure legends.<br><br>Have you included all the information requested in your manuscript?	Yes
**Resources**<br><br>A description of all resources used, including antibodies, cell lines, animals and software tools, with enough information to allow them to be uniquely identified, should be included in the Methods section. Authors are strongly encouraged to cite Research Resource Identifiers (RRIDs) for antibodies, model organisms and tools, where possible.<br><br>Have you included the information requested as detailed in our Minimum Standards Reporting Checklist?	Yes
**Availability of data and materials**<br><br>All datasets and code on which the conclusions of the paper rely must be	Yes

either included in your submission or deposited in publicly available repositories (where available and ethically appropriate), referencing such data using a unique identifier in the references and in the "Availability of Data and Materials" section of your manuscript.<br><br>Have you have met the above requirement as detailed in our Minimum Standards Reporting Checklist?	
GigaScience has policies and guidelines in place for the use of generative AI-writing tools such as ChatGPT. If you have used such writing tools to assist with writing the manuscript this must be declared and cited in the text. Authors should not list AI-writing tools and other AI-assisted technologies as an author or co-author and should acknowledge that they are fully responsible for text generated or refined by AI-writing tools.<p><br><br>A summary of use (particularly in the introduction or among methods) needs to be included at the end of the paper, and the outputs should also be included as a supplementary file hosted in GigaDB or other open repositories. Please read our guidelines for more information. <p><br><br>By submitting to GigaScience, you are aware of the journal's AI-writing tools policy, and if you have declared use of such tools below, you have acknowledged this where appropriate in your manuscript and have made a summary of use and outputs available. <p><br><b>AI-assisted writing tools have been used in the preparation of this manuscript?	No

Manuscript Click here to access/download;Manuscript;Manuscript_Feb 28.docx

The molecular basis of octocoral calcification revealed by genome and skeletal proteome analyses

Yanshuo Liang[1,2,3], Kuidong Xu[1,2,3*], Junyuan Li[1,2], Jingyuan Shi[4], Jiehong Wei[1,2,3], Xiaoyu Zheng[1,2,3], Wanying He[3,5], Xin Zhang[3,5]

[1]*Laboratory of Marine Organism Taxonomy and Phylogeny, Qingdao Key Laboratory of Marine Biodiversity and Conservation, Institute of Oceanology, Chinese Academy of Sciences, Qingdao 266071, China*

[2]*Laoshan Laboratory, Qingdao 266237, China*

[3]*University of Chinese Academy of Sciences, Beijing 100049, China*

[4]*CAS Key Laboratory of Marine Ecology and Environmental Sciences, Institute of Oceanology, Chinese Academy of Sciences, Qingdao 266071, China*

[5]*Key Laboratory of Marine Geology and Environment & Center of Deep Sea Research, Institute of Oceanology, Center for Ocean Mega-Science, Chinese Academy of Sciences, Qingdao 266071, China*

***Correspondence address**. Kuidong Xu. No. 7 Nanhai Road, Qingdao City, Shandong Province, China. Institute of Oceanology, Chinese Academy of Science. E-mail: kxu@qdio.ac.cn

ORCID iDs: Yanshuo Liang [0000-0001-5179-5956]; Jiehong Wei [0000-0001-6401-6747].

Abstract

The ability of octocorals and stony corals to deposit calcium carbonate ($CaCO_3$) has contributed to their ecological success. While stony corals possess a homogeneous aragonite skeleton, octocorals have developed distinct skeletal structures composed of different $CaCO_3$ polymorphs and skeletal organic matrix. Nevertheless, the molecular basis of skeletal structure formation in octocorals remains inadequately understood. Here, we sequenced the genomes and skeletal proteomes of two calcite-forming octocorals, namely *Paragorgia papillata* and *Chrysogorgia* sp. The assembled genomes sizes were 618.13 Mb and 781.04 Mb for *P. papillata* and *Chrysogorgia* sp., respectively, with contig N50s of 2.67 Mb and 2.61 Mb. Comparative genomic analyses identified 162 and 285 significantly expanded gene families in the genomes of *P. papillata* and *Chrysogorgia* sp., respectively, which are primarily associated with biomineralization and immune response. Furthermore, comparative analyses of skeletal proteomes demonstrated that corals with different $CaCO_3$ polymorphs share a fundamental toolkit comprising cadherin, von Willebrand factor type A, and carbonic anhydrase domains for calcified skeleton deposition. In contrast, collagen is rich in the calcite-forming octocoral skeletons but occurs rarely in aragonitic stony corals. Additionally, certain collagens have developed domains related to matrix adhesion and immunity, which may confer novel genetic functions in octocoral calcification. These findings enhance our understanding of the diverse forms of coral biomineralization processes and offer preliminary insights into the formation and evolution of the octocoral skeleton.

Keywords Octocorallia, genomes, $CaCO_3$ polymorphs, skeletal proteomes, biomineralization toolkit

Introduction

The history of $CaCO_3$ biomineralization by organisms spans at least 541 Myr, and biomineralization as an innovative mechanism in the evolutionary history of life has played a significant role in species development and global carbon cycles [1]. The class Anthozoa, an ecologically important and morphologically diverse clade of metazoans, produces extensive biogenic structures through their ability to form colonies and precipitates $CaCO_3$ skeletons to support the entire coral ecosystems in both shallow and deep waters. The ability to produce $CaCO_3$ skeletons is found in two distinct clades of Anthozoa, namely the order Scleractinia (stony coral, subclass Hexacorallia) and the subclass Octocorallia (octocoral). As the primary reef builders, stony corals possess homogeneous aragonite skeletons, and their calcification process has been elucidated through skeletal proteome analysis and immunohistochemical verification [2–5]. In contrast, octocorals have evolved diverse skeletal structures, mainly including different $CaCO_3$ polymorphs (i.e., aragonite or calcite) and organic components (e.g., gorgonin) as well as different types of sclerites [6, 7]. Thus, skeletons of octocorals provide a unique opportunity to compare different calcification strategies involving varied skeletal structures and $CaCO_3$ polymorphs with those of stony corals.

The formation of coral skeletons is biologically controlled by the supply of ions required for $CaCO_3$ deposition and the secretion of a diverse organic matrix at the site of calcification (Supplementary Fig. S1) [8–10]. Major components of the organic matrix include proteins, carbohydrates and lipids [6, 8]. Despite comprising a minimal portion of the coral skeletal organic matrix space, the organic matrices secreted by the calicoblastic ectoderm play an important role in promoting nucleation, growth, and spatial orientation of various $CaCO_3$ polymorphs [11]. A

previous study showed that, although mollusks possess a set of conserved biomineralization-related proteins, the calcite and aragonitic layers within the shell use specific shell matrix proteins to deposit different polymorphs [12]. A fundamental question related to coral calcification is the mechanisms by which corals regulate calcite and aragonitic polymorphs through skeletal organic matrix proteins (SOMPs), and how they control the development of complex and diverse skeletal structures. However, the lack of comprehensive genomic and proteomic data for octocorals has constrained our understanding of the molecular mechanisms underlying the formation of skeletal structures with different $CaCO_3$ polymorphs.

In the present study, we generated draft genomes of two calcite-forming octocorals *Paragorgia papillata* (NCBI:txid2853639; marinespecies.org:taxname:1545268) and *Chrysogorgia* sp. (NCBI:txid3051262) and characterized their skeletal proteomes. We further performed comprehensive phylogenetic analyses, gene family evolution studies, and comparative skeletal proteomic analyses to understand the molecular mechanisms of skeleton formation in octocorals. The obtained genome and proteome information can contribute significantly to our understanding of the molecular mechanisms of coral skeletal formation and its evolutionary development.

Methods

Sample collection and DNA extraction

Samples of *P. papillata* and *Chrysogorgia* sp. were collected using the submersible vehicles *Faxian* and *Jiaolong* from seamounts of the Caroline Ridge (10°06'46.80"N, 140°14'31.79"E, 858 m deep) and the Kyushu-Palau Ridge (13°20'18.24"N, 134°33'37.44"E, 2,086 m deep) in the

tropical Western Pacific (Figs. 1A, 1B). The coral samples were preserved in a sealed sample chamber placed inside the sample basket of the submersible. Following recovery, the samples were sectioned into small pieces and immediately preserved in liquid nitrogen. All experimental protocols were approved by the relevant guidelines and regulations established by the Institutional Animal Care and Use Committee of the Institute of Oceanology, Chinese Academy of Sciences. Genomic DNA was extracted from the polyps by using the MagAttract HMW DNA kit (Qiagen, Germany). The quality and quantity of the extracted DNA were validated with standard agarose gel electrophoresis and a Qubit Fluorometer, respectively.

Illumina sequencing and genome size estimation

Paired-end libraries with insert sizes of 300 base pairs were constructed using the TruSeq DNA Sample Prep Kit in accordance with the manufacturer's instructions. The resulting libraries were then sequenced on an Illumina NovaSeq 6000 platform (RRID:SCR_016387). Low-quality reads and sequencing-adapter-contaminated reads were trimmed using Trimmomatic-0.36 (RRID:SCR_011848). A *k*-mer frequency distribution map of the clean reads was constructed to estimate the genome size, heterozygosity, and proportion of repetitive sequences by using the Jellyfish v.2.2.7 (RRID:SCR_005491) [13]. The genome size (G) was calculated using the following formula: $G = K_{num}/K_{depth}$, where K_{num} is the number of *k*-mers and K_{depth} is the peak depth. The trimmed Illumina paired-end reads were assembled into scaffolds using SOAPdenovo v.2.04 (RRID:SCR_010752) [14] with the following specified parameters: -K 45 -d 1 -D 1 -F.

PacBio sequencing and genome assembly

High-molecular-weight genomic DNA (gDNA) was used for constructing Pacific Biosciences (PacBio) sequencing libraries. The gDNA was fragmented with the g-TUBE device (Covaris) to achieve a size range of 6–20 kb to construct 20 kb libraries. The fragmented DNA was then concentrated and purified using AMPure XP beads (Agencourt). The SMRTbell Template Prep Kit reagents were used to repair various DNA damage, including abasic sites, nicks, thymine dimers, blocked 3′-ends, oxidized guanines/pyrimidines, and deaminated cytosines. T4 DNA polymerase was utilized to polish the ends of the fragments deemed suitable for ligation. The SMRTbell hairpin adapters were then ligated to the repaired ends. Subsequently, size selection was conducted by BluePippin electrophoresis (Sage Science), with a cutoff threshold size of 20 kb. Subsequently, AMPure PB Beads were used to concentrate and purify the SMRTbell templates after size selection. Finally, these purified SMRTbell templates were utilized for primer and polymerase binding. The SMRTbell libraries were then sequenced on a Pacbio Sequel II platform (RRID:SCR_017990).

PacBio long reads were subjected to quality controlled by using SequelQC software (RRID:SCR_017279), and the clean reads were corrected using the error correction module of Canu v.1.5 (RRID:SCR_015880) [15] to select longer subreads. Contaminated reads containing chloroplast, mitochondrial, bacterial or viral sequences were removed through comparison of the genome assembly with the nucleotide sequence database from the National Center for Biotechnology Information (NCBI) using BLASTN v.2.2.26 with an e-value threshold of $\leq$ 1e−5. The data were then assembled using NextDenovo v.2.2 (RRID:SCR_025033) with default parameters. The raw assembly was subjected to three rounds of polishing with Illumina short reads using Pilon (RRID:SCR_014731) [16]. Finally, the PacBio reads were aligned to the initial

assembly by using minimap2 v.2.24-r1122 (RRID:SCR_018550) with the parameter: -x map-bp. Haplotigs and contig overlaps in the resulting assembly were eliminated using Purge_dups v.1.2.5 (RRID:SCR_021173) [17] with the parameter minimumAlignmentScore 70 for *P. papillata* and minimumAlignmentScore 80 for *Chrysogorgia* sp. To evaluate the accuracy of the genome assembly, the Illumina reads were first mapped to the genome assembly by using bwa v.0.7.10 (RRID:SCR_010910). The completeness of the genome assembly was assessed by mapping 954 metazoan benchmarking universal single-copy orthologues to the genome by using BUSCO v.5.0 (RRID:SCR_015008) [18].

Transcriptome sequencing

Total RNA was extracted from the polyps of *P. papillata* and *Chrysogorgia* sp. by using Invitrogen TRIzol reagent (Thermo Fisher Scientific) by following the manufacturer's instructions. The integrity and quality of the extracted RNA were evaluated using the Fragment Analyzer 5400 (Agilent Technologies). Sequencing libraries were generated using the NEBNext® UltraTM RNA Library Prep Kit for Illumina® (NEB, USA) in accordance with the manufacturer's instructions, with an insert size of 300–500 bp. Illumina RNA sequencing (RNA-seq) libraries were prepared and sequenced on the Illumina NovaSeq 6000 platform, resulting in 150-bp paired-end reads. After performing quality score-based trimming using Trimmomatic-0.36, the clean reads were aligned to the coral genomes by using StringTie v. 2.1.5 (RRID:SCR_016323) [19].

Genome annotation

The protein-coding genes were annotated through a combination of *ab initio* prediction methods,

homology searches, and RNA sequencing. *Ab initio* gene prediction was performed using Augustus v.3.1.0 (RRID:SCR_008417) and SNAP v.2006-07-28 (RRID:SCR_007936) with default parameters. For the homolog-based approach, GeMoMa v.1.7 (RRID:SCR_017646) [20] software was performed by using reference gene model from a selection of other cnidarians: *Acropora digitifera*, *Acropora millepora*, *Astreopora myriophthalma*, *Dendronephthya gigantea*, *Porites australiensis*, *Paramuricea clavata*, and *Stylophora pistillata*. Gene prediction based on the RNA-seq data was conducted by aligning clean RNA-seq reads to the reference genome using Hisat2 v.2.0.4 (RRID:SCR_015530) [21] and assembling them with StringTie v.2.1.5. The coding regions were predicted using GeneMarkS-T v.5.1 (RRID:SCR_017648) [22] and PASA v.2.0.2 (RRID:SCR_014656) [23]. Gene models from these different approaches were integrated using EVM v.1.1.1 with default parameters (RRID:SCR_014659) [24] and updated by PASA. The weights assigned to *ab initio* prediction, protein alignment, and transcript were 4, 7, and 8, respectively. The final gene models were annotated by searching against the GenBank Non-Redundant, Gene Ontology, KEGG, and SwissProt databases, with an E-value threshold of 1×10^{-5}. Additionally, these predicted genes were annotated against the Pfam database using HMMER v.3.3.2 (RRID:SCR_005305) software.

Transposable elements (TEs) were analyzed using the RepeatModeler pipeline v.2.0.1 (RRID:SCR_015027) [25] and LTR_retriever v.2.9.0 (RRID:SCR_017623) [26]. Initially, RECON v.1.0.8 (RRID:SCR_021170), RepeatScout v.1.0.6 (RRID:SCR_014653), LTRharvest v.1.5.10 (RRID:SCR_018970), and LTR_FINDER v.1.0.7 (RRID:SCR_015247) were utilized to construct a *de novo* repeat library using default parameters. The predicted repeats were classified using RepeatClassifier and integrated with the Dfam database v.3.5. Subsequently, RepeatMasker

v.4.1.2 (RRID:SCR_012954) [27] was used to identify the divergence of TEs in the coral genomes based on the constructed repetitive sequence database. The repeat landscape was obtained using a modified R script from GitHub.

Phylogenetic analysis and gene expansion and contraction

The orthologue groups (OGs) were identified through a BLASTp search of protein sequences from the genomes of 19 anthozoans and *Hydra vulgaris* (outgroup) (Supplementary Table S1). The BLASTp results were used by OrthoFinder v.2.4.0 (RRID:SCR_017118) [28] to construct OGs. To construct phylogenetic relationships, the protein sequences from 275 single-copy orthologues were extracted from all 20 species and analyzed through multiple alignment using MAFFT v.7.310 (RRID:SCR_011811). Subsequently, poorly aligned regions were trimmed using Gblocks v.0.91b (RRID:SCR_015945), and all alignments were combined into one supergene. ModelFinder software was used to identify the optimal model for the trimmed alignment, and the maximum likelihood tree was generated using IQtree v.2.2.0 (RRID:SCR_017254) [29] with 1,000 bootstrap replicates. The divergence times were estimated using the MCMCTree program from PAML v.4.9j (RRID:SCR_014932) [30] with a correlated rates molecular clock. Five fossil calibration points (Supplementary Table S2) were selected for dating the phylogeny of anthozoans. Finally, the OGs comprising >100 copies in a single species were excluded, and the remaining OGs were employed for the gene family expansion and contraction analysis using CAFÉ v.4.2.1 (RRID:SCR_005983) [31] with the parameter lambda -s and estimated divergence times between species as the input. An event of significant expansion or contraction was considered only when the gene family-wide p-value was <0.01 and the taxon-specific Viterbi p-value was <0.05. The

significantly expanded and contracted gene families were extracted for the Gene Ontology term enrichment analysis with Fisher's exact test, and the *p*-value was adjusted for multiple testing by using the False Discovery Rate method.

Morphological observation, $CaCO_3$ polymorphs analysis, and van Gieson staining of octocoral skeletons

To observe the skeletal ultrastructure, the axial skeletons of *P. papillata* and *Chrysogorgia* sp. were isolated by digestion of the tissues in a sodium hypochlorite solution and washed repeatedly by multiple rinses in milli-Q water. The axial skeletons were subsequently mounted on carbon double-adhesive tape, air-dried, and coated for scanning electron microscopy (SEM) examination. SEM scans were performed using a Hitachi TM3030Plus scanning electron microscope at 15 kV and optimal magnification for each axial skeleton. To determine the $CaCO_3$ polymorphs of coral skeletons, confocal Raman spectroscopy (Alpha 300R+, WITec, Ulm, Germany) was conducted to detect the axial skeleton after the removal of the coenenchyme. van Gieson (VG) staining was performed to determine the distribution of collagen fibers in axial skeletons. The protocol involved the following steps. First, the decalcified axial skeleton was embedded in paraffin, dewaxed with xylene and ethanol, and stored in tap water. Next, the samples were treated with the VG staining solution (Servicebio) for 1 min, rinsed rapidly with water, and dehydrated rapidly in three grades of anhydrous ethanol. Finally, the slides were immersed in xylene until they were transparent, coverslipped with neutral resin, observed under a microscope, and photographed.

Proteomics analysis

The axial skeletons of *P. papillata* and *Chrysogorgia* sp. were bleached in a 10% hypochlorite solution for 5 h to remove the tissue and other potential contaminants. Subsequently, the skeletons were thoroughly rinsed with milli-Q water and dried overnight at 60°C. The dried axial skeletons were pulverized to a fine powder in liquid nitrogen, and bleached again, rinsed, and dried. The skeleton powder was decalcified with 10% acetic acid for 24 h at room temperature on an orbital shaker, and the decalcified solution was centrifuged (14,000 × g, 10 min, 4°C) to separate the acid soluble matrix (ASM) and acid insoluble matrix (AIM). The resulting insoluble pellets (AIM) were rinsed repeatedly with milli-Q water, lyophilized, and reconstituted with 8 M urea (with 1% SDS). Both AIM and ASM were concentrated using Amicon Ultrafiltration devices (15 mL, 10 kDa cutoff), purified with methanol/chloroform, and subsequently reconstituted in 8 M urea.

The ASM and AIM samples were dissolved in solubilization buffer (1% SDS, 10 mM DTT, 50 mM Tris-HCl (pH 8.0)) for sodium dodecyl sulfate-polyacrylamide gel electrophoresis. The samples were subsequently prepared for HPLC-MS/MS analysis through a series of steps, including reduction, alkylation, trypsin digestion, drying, and solubilization. Label-free mass spectrometry was conducted using a Thermo Orbitrap Fusion mass spectrometer. The scan events were configured as a full MS scan in the range of 250–1450 m/z at a mass resolution of 120,000, followed by CID MS/MS scan repetition on the 20 most abundant ions selected from the previous full MS scan with an isolation window. The resulting MS raw data were imported into MaxQuant v. 1.5.2.8 (RRID:SCR_014485) [32] and compared against their respective genomic data. For this study, proteins were considered identified if they exhibited a spectral count exceeding 2 in each sample Identified proteins with at least two distinct peptides were considered for the analysis.

Protein annotation was performed based on sequence similarity search against the NR

database in NCBI and the UniProtKB/SwissProt database using BLASTP with an E-value threshold of 1×10^{-5}. Protein sequences were analyzed for signal peptides and transmembrane domains by using Signal IP v.5.0 (RRID:SCR_015644) and TMHMM v.2.0 (RRID:SCR_014935), respectively. Conserved domains were detected using the InterproScan platform (RRID:SCR_005829). In previous studies, protein identification was based on matching nucleotides or EST databases with unique peptides, which resulted in incomplete functional annotations. Here, we conducted a comparative analysis of the domains of SOMPs by including these two octocorals and two aragonitic scleractinians (*A. millepora* and *S. pistillata*) [3, 4]. The interspecies comparison of the SOMPs from each species was performed using a locally installed NCBI BLAST tool (v.2.2.25+).

Results

Genomic characteristics of *P. papillata* and *Chrysogorgia* sp.

Using a combination of PacBio long reads and Illumina short reads (Supplementary Fig. S2 and Supplementary Table S3), we generated high-quality genomes for *Paragorgia papillata* and *Chrysogorgia* sp. The genome sizes of *P. papillata* (618.13 Mb) and *Chrysogorgia* sp. (781.04 Mb) closely agreed with the *k*-mer-based estimates of 596.50 Mb and 774.93 Mb, respectively (Supplementary Fig. S3 and Supplementary Table S4). The contig N50 of the *P. papillata* assembly is 2.67 Mb and the *Chrysogorgia* sp. assembly is 2.61 Mb (Supplementary Table S4). The integrity of genome assembly was evaluated by back-mapping one library of paired-end data for each coral to its respective assembly. The analysis revealed that 99.34% (*P. papillata*) and 99.40% (*Chrysogorgia* sp.) of the Illumina paired-end reads aligned to the assembled genomes

(Supplementary Table S5). BUSCO analysis with the metazoan database showed that the genome assemblies of *P. papillata* and *Chrysogorgia* sp. contained 91.61% and 88.36% complete BUSCO genes, respectively. Moreover, the proportion of duplicated BUSCO genes in both genomes was 1.89%, which was comparable to that in previously published octocoral genomes (Supplementary Table S6).

The genomes of *P. papillata* and *Chrysogorgia* sp. have a relatively high number of protein-coding genes as compared to the genomes of other anthozoans. Through the integration of multiple methodologies, 41,723 and 52,329 protein-coding genes (PCGs) were predicted in the genomes of *P. papillata* and *Chrysogorgia* sp., respectively (Supplementary Table S7). Among these PCGs, 37,974 (91.01%) in *P. papillata* and 47,126 (90.06%) in *Chrysogorgia* sp. were assigned functional annotations by comparing with public databases, including SwissProt, Pfam, NR, TrEMBL, eggNOG, KOG, KEGG, and GO (Supplementary Table S8). While considerable variation exists in the number of PCGs among different corals, octocoral genomes typically demonstrate higher counts than hexacoral genomes (Supplementary Table S9). The BUSCO completeness of the predicted genes in each genome was 94.23% for *P. papillata* and 92.87% for *Chrysogorgia* sp. (Supplementary Table S10), indicating that our predicted genes are highly complete. TEs influence genome evolution by modifying genomic architecture and affecting gene expression regulation. Using a combination of homology-based and de novo approaches, 294.04 Mb and 374.71 Mb (47.58% and 47.98%) of the *P. papillata* and *Chrysogorgia* sp. genomes (respectively) were identified as TEs (Fig. 1C; Supplementary Table S11 and S12), with 23.76% and 23.84% of these TEs being class II DNA transposons and 23.81% and 24.14% of these TEs being class I retrotransposons (long interspersed nuclear elements [LINEs], long terminal repeats

[LTRs], and short interspersed nuclear elements [SINEs]). Additionally, Kimura distance-based copy divergence analysis revealed similar expansion patterns for TEs across different coral lineages, except for *Trachythela* sp. (Tsp), with high compositional similarity (Fig. 1C; Supplementary Fig. S4).

Phylogenomic analysis and gene-family evolution

The results of phylogenetic analysis clearly showed an Ediacaran origin for Anthozoa and the reciprocal monophyly of the subclasses Octocorallia and Hexacorallia (Fig. 1D). The five octocorals examined belonged to two newly established orders Scleralcyonacea (*P. papillata* [Ppap] and *Chrysogorgia* sp. [Csp]) and Malacalcyonacea (*Paramuricea clavata* [Pcla], Tsp, and *Dendronephthya gigantea* [Dgig]). Ppap and Csp formed a sister group, and their divergence was estimated at approximately the Triassic-Jurassic boundary (181 Ma), which corresponds to the transition period from aragonitic to calcite seas. The remaining three octocorals, i.e., Dgig, Pcla, and Tsp, originated in calcite seas during the Jurassic to Cretaceous periods. Additionally, the results supported the monophylies of Actiniaria (true sea anemones), Corallimorpharia (naked corals, mushroom anemones), and Scleractinia (stony corals) within Hexacorallia. The divergence between Scleractinia and Corallimorpharia (Amplexidiscus fenestrafer [Afen] and *Discosoma* sp. [Dsp]) occurred at 281 Ma (95% confidence interval: 349–221 Ma). Stony corals evolved the capacity to deposit aragonitic crystals in typical aragonitic seas during the Late Carboniferous to Triassic periods (281–214 Ma). Subsequently, stony corals diversified into two crown clades (“robust” and “complex”) in aragonitic seas during the mid-Triassic period (228–193 Ma).

Comparative analyses among a selection of the available anthozoan genomes showed that

286 gene families were expanded in *P. papillata* and 444 in *Chrysogorgia* sp., with 162 and 285 gene families showing significant expansion, respectively (Viterbi p-value < 0.05) (Fig. 1D; Supplementary Tables S13 and S14). The GO enrichment analysis of the expanded gene families identified 24 overrepresented GO categories in both *P. papillata* and *Chrysogorgia* sp. genomes (Supplementary Fig. S5). The significantly expanded gene families were involved in multiple processes: the phosphatidylinositol signaling pathway (PIP5 kinase activity and G protein-coupled neurotransmitter receptor), cell-cell adhesion (cadherin binding and actinin binding), ion transport processes (potassium channel regulator, vacuolar transport, and endosomal transport), and immune-related pathways (e.g., scavenger receptor activity, immunoglobulin production, and T cell receptor signaling pathway); this finding suggests their contributions to both biomineralization and immune responses.

Skeletal structure characterization and biomineralized protein toolkit

To determine the types of the octocoral skeletons, we utilized Raman spectroscopy and scanning electron microscopy to analyze the skeletal structures. We found calcified sclerites embedded at both polyps and coenosarc tissues in *P. papillata* and *Chrysogorgia* sp. The axial skeleton of *P. papillata* showed the accumulation of high-Mg calcite (HMC) sclerites with diverse morphologies in the form of a ring of regularly arranged central pores. In contrast, the axial skeleton of *Chrysogorgia* sp. exhibited a completely calcified HMC structure with a growth pattern resembling that of annual rings (Fig. 2; Supplementary Fig. S6).

We further investigated the molecular basis of skeletal formation in octocorals and identified 64 and 37 SOMPs in the skeletal organic matrix space of *P. papillata* and *Chrysogorgia* sp.,

respectively (Supplementary Tables S15 and S16) by using LC-MS/MS protein sequencing and reference genome search. These SOMPs were validated by multiple unique peptides (Supplementary Tables S17 and S18). To characterize the conserved biomineralization toolkit, we performed a comparative skeleton proteomics analysis of the two calcite-forming octocorals and the two aragonitic scleractinians, *A. millepora* and *S. pistillata*. To detect multiple domains in the same protein from different evolutionary sources, we performed domain prediction and further compared the SOMPs in their functional context. Despite substantial differences in the skeletal morphology and microstructures of corals, we identified the following three functional domains common to the coral skeletal organic matrix space across all the four species (Fig. 2): cadherin, von Willebrand factor type A (VWA), and carbonic anhydrase (CA). The cadherin domain, which contains conserved cysteine residues and calcium-binding motifs, is involved in intercellular adhesion, and this domain was exclusively detected in protocadherin-like or classical cadherin (Supplementary Tables S15 and S16), which belongs to the cadherin superfamily. The VWA domain was identified in protocadherin, collagen, and fibrillin-2 (Supplementary Tables S15 and S16). CA occurred in a superfamily of predominantly zinc-binding metalloenzymes that catalyze the interconversion of CO_2 into HCO_3^-, all of which contain predicted signal peptides or transmembrane domains (Supplementary Tables S15 and S16).

Function and composition of SOMPs

The SOMPs embedded within octocoral skeletons can be classified into five main categories according to their domain function prediction, namely cell adhesion, structure support, immune regulation, enzymes, and other functional proteins (Fig. 2; Supplementary Tables S15 and S16).

The composition of SOMPs largely varied among corals with different skeletal types, and each coral retained distinct functional domains, with the proportion of unique functional domains of up to 47% in *P. papillata* (Fig. 2). Compared to stony corals, we found that octocorals possess numerous proteins containing immunity-associated domains, including alpha-2-macroglobulin, spondin 2, agrin, and putative defense protein 3 (Fig. 2; Supplementary Tables S15 and S16). The presence of these proteins is consistent with the expansion of gene families related to immune regulation (Supplementary Fig. S5), suggesting the presence of immune regulatory pathways within the coral skeletal organic matrix that enhance defense mechanisms during skeletal formation and prevent pathogen invasion.

We identified 7 and 5 collagen types in the skeletons of *P. papillata* and *Chrysogorgia* sp., respectively, and the helical regions of all collagens exhibited the characteristic Gly-X-Y periodic repeats (Fig. 3A; Supplementary Tables S15 and S16). Pfam domain analysis revealed that some collagens have undergone recombination with VWA, WAP, and laminin G domains, potentially contributing to the diverse functions of collagens. To observe the distribution of collagen fibers in tissues, VG staining was performed on the axial skeleton of *P. papillata* and *Chrysogorgia* sp. The results showed that collagen fibers in *P. papillata* were mainly distributed on the calcified sclerites along its unconsolidated and unfused scleritic axis; in contrast, collagen fibers in *Chrysogorgia* sp. appeared deep red throughout the axial skeleton, indicating their widespread distribution in the mineralized skeleton (Figs. 3B, 3C; Supplementary Fig. S7).

Discussion

This study presents the sequenced and assembled draft genomes and skeletal proteomes of two

octocoral species, *P. papillata* and *Chrysogorgia* sp., contributing to the growing collection of octocoral genomes and enhancing our understanding of skeletal formation and evolutionary history in octocorals. We found that, except for *P. clavata*, the genome sizes of the two octocoral species, *P. papillata* and *Chrysogorgia* sp., were considerably larger than those of the published genomes of octocorals and hexacorals; this could be potentially attributed to the large number of repetitive sequences in the genomes of *P. papillata* and *Chrysogorgia* sp. Phylogenetic analyses revealed that the five calcite-forming octocorals examined belonged to two newly established orders, namely Scleralcyonacea and Malacalcyonacea [33], all of which emerged in calcite seas during the Jurassic to Cretaceous periods. The origin of corals with different $CaCO_3$ polymorphs is generally considered to be related to the palaeoclimate ocean conditions [34], suggesting that elevated Mg/Ca ratios in calcite seas may have favored calcite skeleton formation in octocorals [35].

The skeletal structure of corals contains an embedded organic matrix with a specific set of proteins that can stabilize amorphous calcium carbonate and regulate the nucleation, orientation, and polymorph selection [9, 36]. Understanding the composition of SOMPs in the coral skeleton is essential for elucidating the ancient mechanisms underlying coral skeleton formation and evolution. By comparing the proteomes of different coral skeletons, this study revealed a conserved protein toolkit utilized by both calcite-forming octocorals and aragonitic stony corals for biomineralization. Despite variations in skeletal morphology and polymorphs, the biomineralization toolkit composed of cadherin, VWA, and CA is evolutionarily conserved and represents a fundamental component of the biomineralization process for skeletal construction. The cadherin domain, a Ca^{2+}-dependent transmembrane glycoprotein present in both

protocadherin and classical cadherin, belongs to extracellular matrix (ECM)-like proteins. A potential role of cadherin in coral skeleton formation is mediating connections between calicoblastic cells and the organic matrix within the skeleton [4, 5]. The VWA domain is predominantly associated with proteins involved in cell adhesion and structural support. This domain interacts with chitin or fibronectin to form a cross-linked organic matrix network, thereby guiding skeletal growth and morphological differentiation [11, 37]. CA is a key enzyme involved in a wide range of physiological functions and is present in all metazoan clades [38, 39]. CA proteins identified in the skeletal proteomes of octocorals and stony corals possess transmembrane domains or signaling peptides, suggesting their role as secreted or membrane-associated CAs that catalyze the interconversion of CO_2 to HCO_3^- in the ECM and provide inorganic carbon for $CaCO_3$ precipitation.

Skeletal proteome analysis and collagen fiber staining revealed substantial collagen presence in octocoral skeleton. Abundant collagen-like proteins have also been detected in the precious red coral *Corallium rubrum* and the gorgonian coral with calcite skeleton [40, 41]. The presence of abundant collagen in the skeletal organic matrix space appears to be a distinctive characteristic of calcite-forming octocorals. Previous studies have indicated that, during the skeleton formation process, the initial collagen triple helix structure contains negatively charged carboxyl groups on its exterior; these groups bind with calcium ions to form mineralized collagen fibers that provide a template for mineral deposition and promote $CaCO_3$ nucleation [42, 43]. This finding suggests that collagen may serve as the fundamental structural framework of octocoral skeletons. We also observed frequent recombination of collagen domains in the axial skeletons of octocorals, including binding to VWA, laminin G, and WAP domains. The binding of new domains may

confer novel genetic functions to collagen during the deposition of $CaCO_3$ skeletal structures. The VWA and laminin G domains, typically found in ECM proteins, are involved in cell-substrate adhesion and the arrangement of CaCO3 crystals [36, 44]. The presence of these proteins may facilitate the cross-linking of collagen with other non-collagenous proteins to establish the core matrix framework. The WAP domain plays a pivotal role in regulating innate immunity, protecting against microbial invasion, and promoting mucosal tissue repair [45]. A previous study demonstrated that proteins involved in innate immune responses can assist stony corals in resisting skeletal pathogen infiltration, thereby enhancing their calcification capacity [46]. Based on these findings, we propose that the binding of the collagen domain to the WAP domain enhances immunity in a matrix framework environment, thereby promoting the deposition of calcified skeleton in octocorals. Octocorals are predominantly passive suspension feeders, and their colonies frequently adopt a clumped, tree-like, or net-like structure oriented toward ocean currents [47]. In this context, collagen might play a critical role in strengthening skeletal structure and enhancing skeletal flexibility to withstand ocean current conditions.

Data Description

This study presents the assembly, annotation, and comparative genomic analyses of genomes of two octocoral species: *P. papillata* and *Chrysogorgia* sp. We also characterized the axial skeletal proteomes of these two octocorals to identify a fundamental toolkit for coral calcification by comparison with the skeletal proteomes of aragonitic corals. We found that collagen in the axial skeleton of octocorals has evolved structural domains linked to matrix adhesion and immunity, which may confer novel genetic functions for calcification in octocorals. The data obtained from

the genomes and proteomes expand the existing octocoral genome database and provide significant insights into the molecular mechanisms and evolutionary history of coral skeletal formation.

Additional Files

Supplementary Figure. This file contains Figure S1-S7.

Supplementary Table. This file contains Table S1-S12.

Supplementary Table S13-S18. This supplementary table contains the annotation file of expansion/contraction gene families in *P. papillata* and *Chrysogorgia* sp. and skeletal proteome analysis file.

Abbreviations

Stony coral: Scleractinia; Octocoral: Octocorallia; $CaCO_3$: Calcium carbonate; SOMPs: Skeletal organic matrix proteins; TEs: Transposable elements; OGs: Orthologue Groups; VG: Van Gieson staining; SEM: Scanning electron microscope; CA: Carbonic anhydrase; SDS-PAGE: Sodium dodecyl sulphate-polyacrylamide gel electrophoresis; ASM: The acid soluble matrix; AIM: The acid insoluble matrix; BUSCO: Benchmarking universal single-copy orthologs; LINEs: Long interspersed nuclear elements; LTRs: Long terminal repeats; SINEs: Short interspersed nuclear elements; HMC: high-Mg calcite; VWA: von Willebrand factor type A; WAP: WAP-type (Whey Acidic Protein) 'four-disulfide core'; ECM: Extracellular calcifying medium.

Acknowledgements

We appreciate the crew of R/V Kexue and ROV Faxian and HOV Jiaolong for their assistance on sample and data collection. We thank Dr. Zifeng Zhan, Yang Li, Yu Xu, and Dongsheng Wang for providing assistance in collecting and preserving samples at sea. Thanks to Dr. Yu Xu and Dr. Ting Lv for their assistance in using scanning electron microscopy. Thanks to Oceanographic Data Center, Institute of Oceanology, Chinese Academy of Sciences for providing computing power for comparative genomics analysis.

Author's Contributions

YS Liang and KD Xu conceived and designed the project. YS Liang, JY Li, JY Shi assembled the genome, annotated the genes, and performed bioinformatics analyses. YS Liang conducted Van Gieson (VG) staining experiments of coral skeletons, and JY Shi photographed the staining pictures. JH Wei revised the abstract and introduction and provided important points. XY Zheng analyzed the collagen domain and mapped it. WY He and X Zhang used Raman spectroscopy to identify coral skeletons and $CaCO_3$ crystals. YS Liang interpreted the data and drafted the manuscript, KD Xu revised the manuscript. All authors discussed the results and approved the final version of this manuscript.

Funding

This study was supported by the National Natural Science Foundation of China (No. 41930533), the Strategic Priority Research Program of the Chinese Academy of Sciences (No. XDB42000000), and the Senior User Project of R/V KEXUE of the Chinese Academy of Sciences (No. KEXUE2020GZ02).

Data Availability

The genomes of the two deep-sea octocoral species investigated in this study have been deposited in the NCBI database under BioProject numbers PRJNA999483 (*P. papillata*) and PRJNA999484 (*Chrysogorgia* sp.). The whole-genome sequencing data and RNA-seq data were deposited in the sequence read archive database under accession numbers SRR25705840-SRR25705842 (*P. papillata*) and SRR25705989-SRR25705991 (*Chrysogorgia* sp.). The genome-related annotation files are accessible through Figshare [48]. The raw data of proteomic sequencing are available in the ProteomeXchange database under project ID IPX0010006000. The specific accessions are provided in the respective Material and Methods sections describing the data and analyses. All additional supporting data are available in the *GigaScience* repository, GigaDB [49-51].

Competing interests

The authors declare that they have no competing interests.

References

1. Gilbert PUPA, Bergmann KD, Boekelheide N, et al. Biomineralization: Integrating mechanism and evolutionary history. Sci adv. 2022;8(10):eabl9653. https://doi.org/10.1126/sciadv.abl9653.

2. Tambutté E, Allemand D, Zoccola D, et al. Observations of the tissue-skeleton interface in the scleractinian coral *Stylophora pistillata*. Coral Reefs. 2007;26(3):517–29. https://doi.org/10.1007/s00338-007-0263-5.

3. Drake J, Mass T, Haramaty L, et al. Proteomic analysis of skeletal organic matrix from the

stony coral *Stylophora pistillata*. Proc Natl Acad Sci USA. 2013;110(10):3788–93. https://doi.org/10.1073/pnas.1301419110.

4. Ramos-Silva P, Kaandorp J, Huisman L, et al. The skeletal proteome of the coral *Acropora millepora*: the evolution of calcification by co-option and domain shuffling. Mol Biol Evol. 2013;30(9):2099–112. https://doi.org/10.1093/molbev/mst109.

5. Takeuchi T, Yamada L, Shinzato C, et al. Stepwise evolution of coral biomineralization revealed with genome-wide proteomics and transcriptomics. PLoS One. 2016;11(6):e0156424. https://doi.org/10.1371/journal.pone.0156424.

6. Conci N, Vargas S, Wrheide G. The biology and evolution of calcite and aragonite mineralization in Octocorallia. Front Ecol Evol. 2021;9:623774. https://doi.org/10.3389/fevo.2021.623774.

7. McFadden CS, Quattrini AM, Brugler MR, et al. Phylogenomics, origin, and diversification of Anthozoans (Phylum Cnidaria). Syst Biol. 2021;70(4):635–47. https://doi.org/10.1093/sysbio/syaa103.

8. Tambutté S, Holcomb M, Ferrier-Pagès C, et al. Coral biomineralization: From the gene to the environment. J Exp Mar Biol Ecol. 2011;408:58–78. https://doi.org/10.1016/j.jembe.2011.07.026.

9. Drake JL, Mass T, Stolarski J, et al. How corals made rocks through the ages. Global Change Biol. 2020;26(1):31–53. https://doi.org/10.1111/gcb.14912.

10. Wang X, Zoccola D, Liew YJ, et al. The evolution of calcification in reef-building corals. Mol Biol Evol. 2021;38(9):3543–55. https://doi.org/ 10.1093/molbev/msab103.

11. Falini G, Fermani S, Gofredo S. Coral biomineralization: a focus on intra-skeletal organic matrix and calcifcation. Semin Cell Dev Biol. 2015;46:17–26.

https://doi.org/10.1016/j.semcdb.2015.09.005.

12. Marie B, Joubert C, Tayalé A, et al. Different secretory repertoires control the biomineralization processes of prism and nacre deposition of the pearl oyster shell. Proc Natl Acad Sci USA. 2012;109(51):20986–91. https://doi.org/10.1073/pnas.1210552109.

13. Marçais G, Kingsford C. A fast, lock-free approach for efficient parallel counting of occurrences of *k*-mers. Bioinformatics. 2011;27(6):764–770. https://doi.org/10.1093/bioinformatics/btr011.

14. Luo RB, Liu BH, Xie YL, et al. SOAPdenovo2: an empirically improved memory-efficient short-read de novo assembler. GigaScience. 2012;1(1):1–18. https://doi.org/10.1186/2047-217X-1-18.

15. Koren S, Walenz BP, Berlin K, et al. Canu: Scalable and accurate long-read assembly via adaptive k-mer weighting and repeat separation. Genome Res. 2017;27(5):722–36. https://doi.org/10.1101/gr.215087.116.

16. Walker BJ, Abeel T, Shea T, et al. Pilon: an integrated tool for comprehensive microbial variant detection and genome assembly improvement. PLoS One. 2014;9(11):e112963. https://doi.org/10.1371/journal.pone.0112963.

17. Guan D, Mccarthy SA, Wood J, et al. Identifying and removing haplotypic duplication in primary genome assemblies. Bioinformatics. 2020;36(9):2896–98. https://doi.org/10.1093/bioinformatics/btaa025.

18. Simão FA, Waterhouse RM, Ioannidis P, et al. BUSCO: assessing genome assembly and annotation completeness with single-copy orthologs. Bioinformatics. 2015;31(19):3210–12. https://doi.org/10.1093/bioinformatics/btv351.

19. Pertea M, Pertea GM, Antonescu CM, et al. StringTie enables improved reconstruction of a transcriptome from RNA-seq reads. Nat Biotechnol. 2015;33(3):290–95. https://doi.org/10.1038/nbt.3122.

20. Keilwagen J, Wenk M, Erickson JL, et al. Using intron position conservation for homology-based gene prediction. Nucleic Acids Res. 2016;44(9):e89. https://doi.org/10.1093/nar/gkw092.

21. Kim D, Langmead B, Salzberg SL. 2015. HISAT: a fast spliced aligner with low memory requirements. Nat Methods. 2015;12(4):357–60. https://doi.org/10.1038/nmeth.3317.

22. Tang S, Lomsadze A, Borodovsky M. Identification of protein coding regions in RNA transcripts. Nucleic Acids Res. 2015;43(12):e78. https://doi.org/10.1093/nar/gkv227.

23. Haas BJ, Delcher AL, Mount SM, et al. Improving the Arabidopsis genome annotation using maximal transcript alignment assemblies. Nucleic Acids Res. 2003;31(19):5654–66. https://doi.org/10.1093/nar/gkg770.

24. Haas BJ, Salzberg SL, Zhu W, et al. Automated eukaryotic gene structure annotation using EVidenceModeler and the program to assemble spliced alignments. Genome Biol. 2008;9(1):R7. https://doi.org/10.1186/gb-2008-9-1-r7.

25. Flynn JM, Hubley R, Goubert C, et al. RepeatModeler2 for automated genomic discovery of transposable element families. Proc Natl Acad Sci USA. 2020;117(17):9451–57. https://doi.org/10.1073/pnas.1921046117.

26. Ou S, Jiang N. LTR_retriever: A highly accurate and densitive program for identification of long terminal repeat retrotransposons. Plant Physiol. 2018;176(2):1410–22. https://doi.org/10.1104/pp.17.01310.

27. Tarailo-Graovac M, Chen N. Using RepeatMasker to identify repetitive elements in genomic sequences. Curr Protocols BioInf. 2009;25:4–10. https://doi.org/10.1002/0471250953.bi0410s25.

28. Emms DM, Kelly S. OrthoFinder: solving fundamental biases in whole genome comparisons dramatically improves orthogroup inference accuracy. Genome Biol. 2015;16:157. https://doi.org/10.1186/s13059-015-0721-2.

29. Nguyen LT, Schmidt HA, von Haeseler A, et al. IQ-TREE: A fast and effective stochastic algorithm for estimating maximum-likelihood phylogenies. Mol Biol Evol. 2015;32(1):268–74. https://doi.org/10.1093/molbev/msu300.

30. Yang Z. PAML 4: phylogenetic analysis by maximum likelihood. Mol Biol Evol. 2007;24(8):1586–91. https://doi.org/10.1093/molbev/msm088.

31. De Bie T, Cristianini N, Demuth JP, et al. CAFE: a computational tool for the study of gene family evolution. Bioinformatics. 2006;22(10):1269–71. https://doi.org/10.1093/bioinformatics/btl097.

32. Cox J, Mann M. MaxQuant enables high peptide identification rates, individualized p.p.b.-range mass accuracies and proteome-wide protein quantification. Nat Biotechnol. 2008;26(12):1367–72. https://doi.org/10.1038/nbt.1511.

33. McFadden CS, van Ofwegen LP, Quattrini AM. Revisionary systematics of Octocorallia (Cnidaria: Anthozoa) guided by phylogenomics. Bull Syst Biol. 2022;1(3):8735. https://doi.org/10.18061/bssb.v1i3.8735.

34. Quattrini AM, Rodríguez E, Faircloth BC, et al. Palaeoclimate ocean conditions shaped the evolution of corals and their skeletons through deep time. Nat Ecol Evol. 2020;4(11):1531–38. https://doi.org/10.1038/s41559-020-01291-1.

35. Yuyama I, Higuchi T. Differential gene expression in skeletal organic matrix proteins of scleractinian corals associated with mixed aragonite/calcite skeletons under low mMg/Ca conditions. PeerJ. 2019;7:e7241. https://doi.org/10.7717/peerj.7241.

36. Rahman MA, Oomori T, Wörheide G. 2011. Calcite formation in soft coral sclerites is determined by a single reactive extracellular protein. J Biol Chem. 2011;286(36):31638–49. https://doi.org/ 10.1074/jbc.M109.070185.

37. Du X, Fan G, Jiao Y, et al. The pearl oyster *Pinctada fucata martensii* genome and multi-omic analyses provide insights into biomineralization. GigaScience. 2017;6(8):1–12. https://doi.org/ 10.1093/gigascience/gix059.

38. Jackson DJ, Macis L, Reitner J, et al. Sponge paleogenomics reveals an ancient role for carbonic anhydrase in skeletogenesis. Science. 2007;316(5833):1893–95. https://doi.org/10.1126/science.1141560.

39. Le Roy N, Jackson DJ, Marie B, et al. The evolution of metazoan α-carbonic anhydrases and their roles in calcium carbonate biomineralization. Front Zool. 2014;11(1):1–16. https://doi.org/10.1186/s12983-014-0075-8.

40. Le Roy N, Ganot P, Aranda M, et al. The skeletome of the red coral *Corallium rubrum* indicates an independent evolution of biomineralization process in octocorals. BMC Ecol Evol. 2021;21:1. https://doi.org/10.1186/s12862-020-01734-0.

41. Goldberg WM. Evidence of a sclerotized collagen from the skeleton of a gorgonian coral. Comp Biochem Phys B. 1974;49(3): 525–26. https://doi.org/10.1016/0305-0491(74)90188-6.

42. Cui FZ, Li Y, Ge J. Self-assembly of mineralized collagen composites. Mater Sci Eng R. 2007;57(1):1–27. https://doi.org/10.1016/j.mser.2007.04.001.

43. Silver FH, Landis WJ. Deposition of apatite in mineralizing vertebrate extracellular matrices: a model of possible nucleation sites on type I collagen. Connect Tissue Res. 2011;52(3):242–54. https://doi.org/10.3109/03008207.2010.551567.

44. Whittaker CA, Hynes RO. Distribution and evolution of von Willebrand/integrin A domains: widely dispersed domains with roles in cell adhesion and elsewhere. Mol Biol Cell. 2002;13(10): 3369–87. https://doi.org/10.1091/mbc.E02-05-0259.

45. Bingle C, Vyakarnam A. Novel innate immune functions of the whey acidic protein family. Trends Immunol. 2008;29(9): 444–53. https://doi.org/10.1016/j.it.2008.07.001.

46. Levy S, Mass T. The skeleton and biomineralization mechanism as part of the innate immune system of stony corals. Fronti Immunol. 2022;13. https://doi.org/10.3389/fimmu.2022.850338.

47. Patterson MR. Passive suspension feeding by an octocoral in plankton patches: Empirical test of a mathematical model. Biol Bull. 1991;180(1):81–92. https://doi.org/10.2307/1542431.

48. Liang Y. Annotation files. FigShare. Dataset. 2023. https://doi.org/10.6084/m9.figshare.23984235

49. Liang Y, Xu K, Li J, et al. Supporting data for "The molecular basis of octocoral calcification revealed by genome and skeletal proteome analyses" GigaScience Database. 2025. https://doi.org/10.5524/102665

50. Liang Y, Xu K, Li J, et al. Genome assembly of the deep-sea octocoral Paragorgia papillata GigaScience Database. 2025. https://doi.org/10.5524/102666

51. Liang Y, Xu K, Li J, et al. Genome assembly of the deep-sea octocoral Chrysogorgia sp. GigaScience Database. 2025. https://doi.org/10.5524/102667

Figure captions

Figure 1: Evolution of the genomes of *P. papillata* and *Chrysogorgia* sp. (A and B) Freshly collected samples of *P. papillata* (A) and *Chrysogorgia* sp. (B). Scale bars=10 cm (a). **(C)** Proportions of DNA transposons and LTR, LINE, and SINE retrotransposons in the genomes of six representative anthozoans including *P. papillata* (Ppap), *Chrysogorgia* sp. (Csp), *D. gigantea* (Dgig), *P. clavata* (Pcla), *Trachythela* sp. (Tsp), and *A. digitifera* (Adig). The tree illustrates the evolutionary relationships among the six corals. The pie charts are scaled according to the genome size (Supplementary Table S9). **(D)** A phylogenetic tree was constructed using 275 single-copy orthologues from 19 anthozoans and *Hydra vulgaris* (outgroup). Divergence time was estimated with the approximate likelihood calculation method together with a correlated rates molecular clock. The 95% confidence interval of the estimated divergence time at each node is denoted as a blue bar. The positive and negative numbers adjacent to the species abbreviations represent gene family numbers of expansion/contraction derived from the CAFE analysis. Species abbreviations in Supplementary Table S1. Geological era abbreviations: To, Tonian; Cr, Cryogenian; Ed, Ediacaran; Cm, Cambrian; O, Ordovician; S, Silurian; D, Devonian; C, Carboniferous; P, Permian; T, Triassic; J, Jurassic; K, Cretaceous; P, Palaeogene; N, Neogene.

Figure 2: Venn diagram of the protein domains identified from the four coral SOMPs. SEM images illustrate the skeletal morphology of these four corals. Domains shown in bright green are related to the structural support of the skeleton. Domains shown in blue are mainly involved in cell adhesion. Immunity-related domains are represented in red.

Figure 3: Structural domain and distribution of collagen in the axial skeleton of *P. papillata* and *Chrysogorgia* sp. (A) Schematic representation of 5 and 7 collagen proteins identified in the proteomes of *P. papillata* and *Chrysogorgia* sp., respectively. **(B)** and **(C)** van Gieson staining results of axial skeletons of *P. papillata* (B) and *Chrysogorgia* sp. (C). The axial skeleton areas containing collagen fibers appear dark red. In *P. papillata*, the wart-like branching structures are sclerites with distributed collagen fibers.

Figure 1

Click here to access/download;Figure;Figure1.pdf

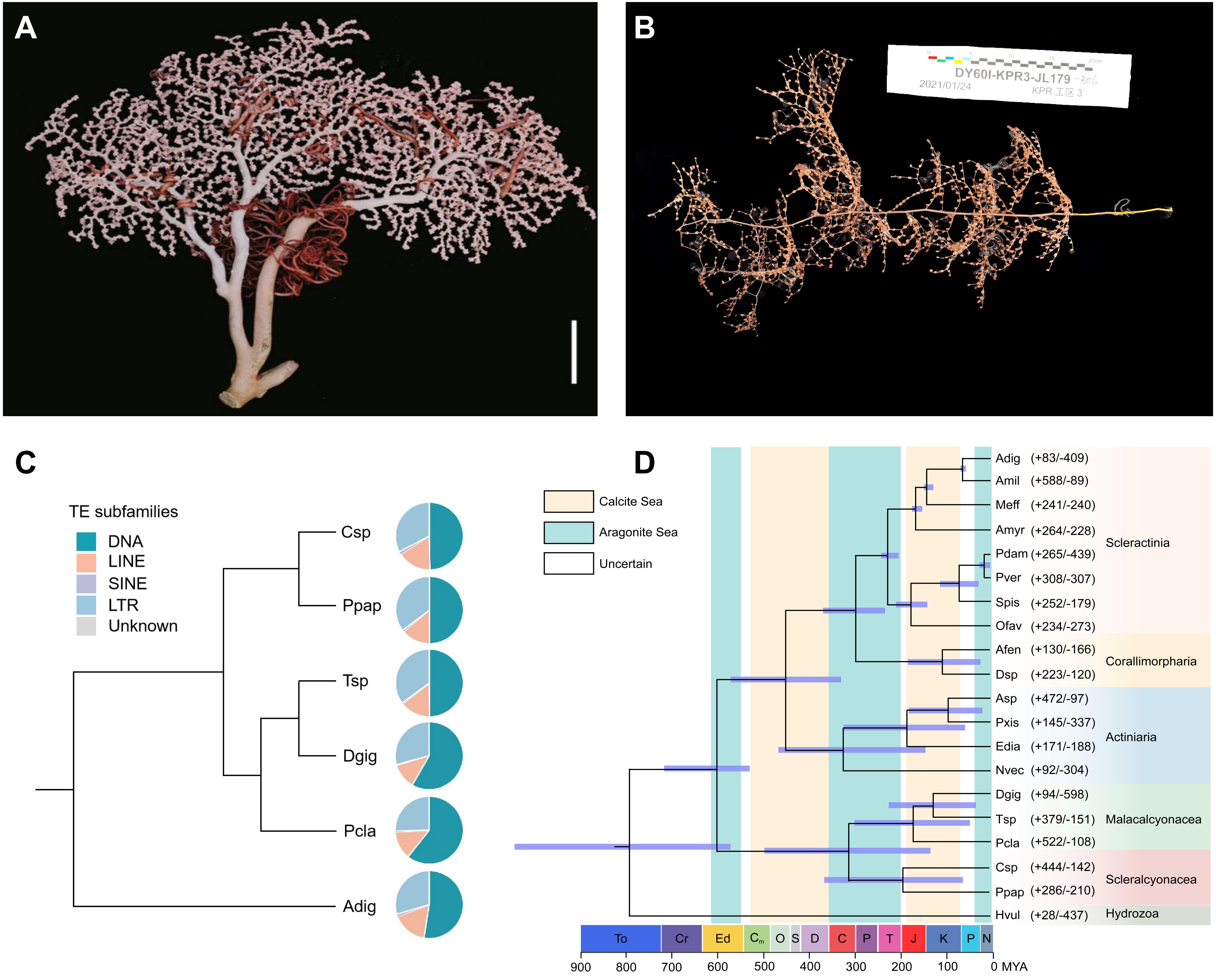

Figure 2

Click here to access/download;Figure;Figure2.pdf

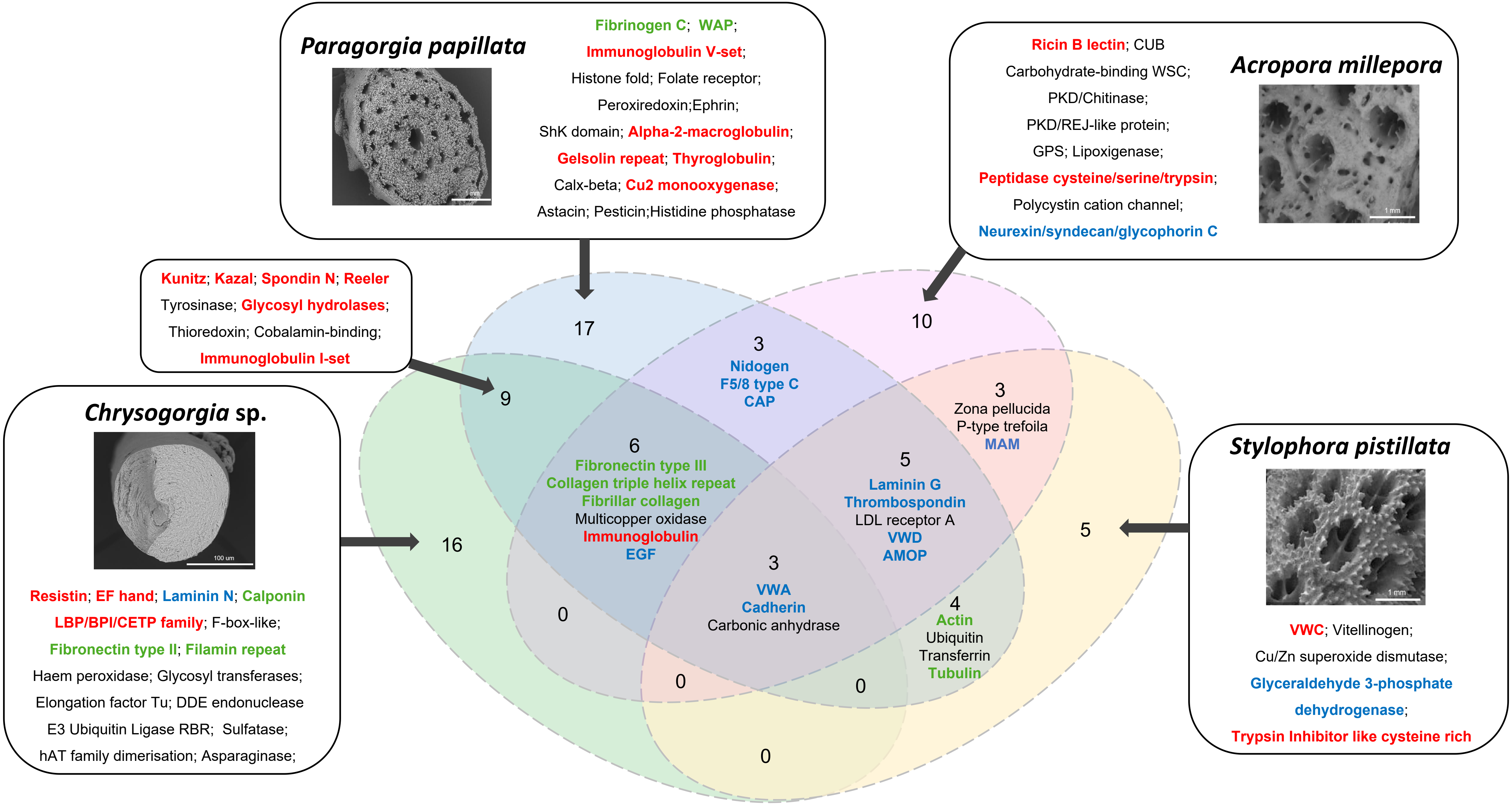

Figure 3

Click here to access/download;Figure;Figure3.pdf

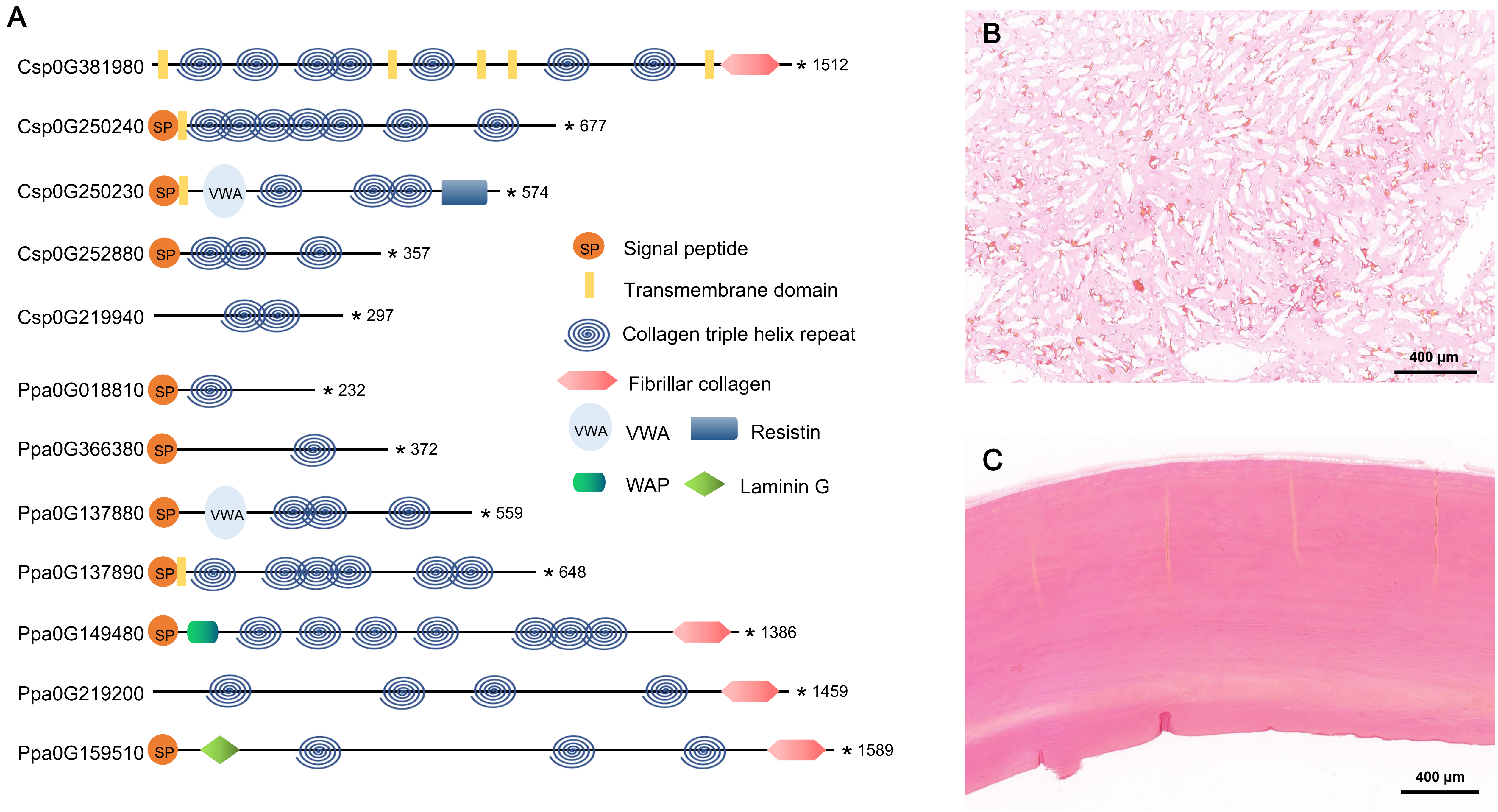

Supplementary Figure

Click here to access/download
Supplementary Material
Supplementary Figure.docx

Supplementary Table

Click here to access/download
Supplementary Material
Supplementary Table.docx

Click here to access/download
Supplementary Material
Supplementary TableS13-S18.xlsx
