# Supplementary material for: The molecular basis of octocoral calcification revealed by genome and skeletal proteome analyses
Source: Gigascience. 2025 Apr 1;14:giaf031. doi: 10.1093/gigascience/giaf031 (PMC11959691; doi:10.1093/gigascience/giaf031)
Supplement: giaf031_Response_to_Reviewer_Comments_Original_Submission [file giaf031_response_to_reviewer_comments_original_submission.pdf]

Author's Response To Reviewer Comments

Response to reviews
We sincerely thank the editor and all reviewers for their valuable feedback that we have used to improve the quality of our manuscript (The molecular basis of octocoral calcification revealed by genome and skeletal proteome analyses; GIGA-D-24-00546). All comments and suggestions play an important role in the correction and improvement of this article. We have carefully revised the article according to the requirements of the editor and reviewers, and made detailed instructions as shown below.

To Reviewer #1:
Question 1: * I think there should be a clear statement of whether either of these species has calcified spicules in addition to an axial skeleton. From the manuscript it appears that neither species has spicules but because they are common in octocorals this should be clarified.
Response: Thanks for this valuable comment. I think your point of view is correct, but I think the spicules you're talking about might be sclerites. In order to give readers a better understanding of these two octocoral morphological characteristics, we have re-explained the calcification structure in the Lines 317-322, as follows:
“To determine the types of the octocoral skeletons, we utilized Raman spectroscopy and scanning electron microscopy to analyze the skeletal structures. We found calcified sclerites embedded at both polyps and coenosarc tissues in P. papillata and Chrysogorgia sp. The axial skeleton of P. papillata was found to consist of the accumulation of high-Mg calcite (HMC) sclerites of varying morphologies, forming a ring of regularly arranged central pores. In contrast, the axial skeleton of Chrysogorgia sp. is a fully calcified, HMC structure with a growth pattern analogous to that of annual rings (Fig. 2; Supplementary Fig. S6).”

Question 2: * the same sorts of grammatical mistakes that I pointed out previously are still present. There are many instances where the articles "the" and "a" are needed and where singular and plural are incorrectly used. For example, in the first sentence of the abstract there is an example of the latter mistake. The word "ability" is singular and determines the verb, which should be "has" not "have".
Response: Thanks for your suggestion. We asked a company specializing in English editing to polish the manuscript.

Question 3: * to repeat my previous comment: "On a related note, the authors are very inconsistent in their use of genus names, sometimes spelling them out and sometimes abbreviating them. The traditional solution to this problem is to spell out genus and species in full on first use (e.g Acropora millepora) and thereafter use an abbreviation (e.g. A. millepora)."yet on lines 153-155 the authors still have" A.digitifera, A. millepora, Astreopora myriophthalma, D. gigantea, Porites australiensis, P. clavata,Stylophora postillata." Is there a reason that they don't want to follow the usual convention?
Response: We were really sorry for our careless mistakes. We have revised it in the manuscript according to your suggestion.

Question 4: *The reply of the authors to question 10 from reviewer 2 is an excellent, succinct summary of their work , which perhaps could be incorporated into their paper. "we focus on the assembly, annotation and underlying comparative genomic analyses of two octocorals. Skeletal proteome analyses led us to identify a basic toolkit for coral calcification. We further found that collagen in the skeleton of octocorals has evolved structural domains associated with matrix adhesion and immunity, which may confer new genetic functions for calcification in octocorals. These genomes and proteomes expand the list of octocoral genomes and provide a valuable resource for understanding the molecular mechanisms of coral skeletal formation and their evolutionary history."
Response: Thanks for this valuable comment. We added the Data Description at the end of the discussion section as summary of our work, as shown below:
Data Description
This study focuses on the assembly, annotation and underlying comparative genomic analyses of two octocoral genomes, P. papillata and Chrysogorgia sp. We further characterized the axial skeletal proteomes of these two octocorals to identify a basic toolkit for coral calcification by comparison with the skeletal proteomes of aragonitic corals. We also found that collagen in the axial skeleton of octocorals has evolved

structural domains associated with matrix adhesion and immunity, which may confer new genetic functions for calcification in octocorals. These genomes and proteomes expand the list of octocoral genomes and provide a valuable resource for understanding the molecular mechanisms of coral skeletal formation and their evolutionary history.

Question 5: * Suppl Table S1 is a very useful addition to the paper
Response: Thank you.

Question 6: * I was unable to view Tables S13-S18. Following S12 there is a page with the following: Table S13-S18 in the Supplementary information section
Response: We put TableS13-S18 in a table execl for Supplementary TableS13-S18.xlsx. This file has been successfully uploaded in the first submission. In order to avoid not seeing it again, I will upload it again when I submit the revision draft.

Question 7: * The captions to the Supplementary figures could use English editing
Response: Thanks for your suggestion. We have tried our best to polish the language in the captions of the Supplementary figures.

To Reviewer #2:
Question 1: Line 20: "have led to their" to "has led to their"
Response: We sincerely thank the reviewer #2 for careful reading. As suggested, we have corrected the "have led to their" to "has led to their"

Question 2: Lines 25-27: "Both assembled genomes size is 618.13 Mb and 781.04 Mb for P. papillata and Chrysogorgia sp. respectively, with contig N50 of 2.67 Mb and 2.61 Mb" to "The assembled genomes sizes were 618.13 Mb and 781.04 Mb for P. papillata and Chrysogorgia sp., respectively, with contig N50s of 2.67 Mb and 2.61 Mb"
Response: Thank you. According to your suggestion, we have made changes in the manuscript.

Question 3: Line 43: Is this what you were trying to say? "Biomineralization of CaCO3 originated in organisms at least 541 Mya"
Response: Thank you. Here we would like to state that the history of biological CaCO3 mineralization has lasted at least 541 Mya. The earliest known CaCO3 skeletons are problematic metazoans found in late Ediacaran (550 to 541 Ma before present) rocks distributed globally (Wood et al, 2002; Gilbert et al, 2022). In order not to mislead the reader, we have rephrased the manuscript as follows:
"The history of CaCO3 biomineralization by organisms spans at least 541 Myr, and biomineralization as an innovative mechanism in the evolutionary history of life has played a significant role in species development and global carbon cycles [1]"

Reference:
Gilbert PUPA, Bergmann KD, Boekelheide N, et al. Biomineralization: Integrating mechanism and evolutionary history. Sci adv. 2022;8(10):eabl9653.
Wood RA, Grotzinger JP, Dickson J. Proterozoic modular biomineralized metazoan from the Nama Group, Namibia. Science. 2002;296(5577):2383-2386.

Question 4: Line 46: "biogenic structures by their ability" to "biogenic structures through their ability"
Response: As suggested, we have made changes in the manuscript.

Question 5: Line 48: "CaCO3 skeleton-producing ability is found" to "The ability to produce CaCO3 skeletons is found"
Response: According to your suggestion, we have made revisions in the manuscript.

Question 6: Line 58: "of diverse organic matrix to calcification site" to "of a diverse organic matrix at the site of calcification"
Response: According to your suggestion, we have made revisions in the manuscript.

Question 7: Line 59: "Major components of organic matrix include proteins" to "Major components of the organic matrix include proteins"
Response: As suggested, we have made changes in the manuscript.

Question 8: Line 103-104: "This was followed by extending, gap filling and polishing the assembly" -> What tools and parameters were used for this? Was it SOAPdenovo or something else? Please elaborate.
Response: Thanks for this comment. Here we did not use specialized software to extend, fill gap and polish the genome, but through SOAPdenovo software to assemble the Illumina paired-end reads and set parameters to achieve these steps (the parameter: -K 45 -d 1 -D 1 -F). We have rephrased it in the manuscript as follows:
"The trimmed Illumina paired-end reads were assembled into scaffolds using SOAPdenovo v2.04 (RRID:SCR_010752) [14] with the following specified parameters: -K 45 -d 1 -D 1 -F."

Question 9: Line 120: "Following the removal of low-quality and duplicate reads" -> Using what tools and parameters? Please elaborate.
Response: Thank you. According to your suggestion, we have rephrased it in the manuscript as follows:
"PacBio long reads were subjected to quality controlled by using SequelQC software (RRID:SCR_017279), and the clean reads were corrected using the error correction module of Canu v.1.5 (RRID:SCR_015880) [15] to select longer subreads."

Question 10: Line 123: "sequences were removed via comparison of the genome assembly" -> Using what tools and parameters? I assume BLASTN, but using what cutoffs? Please elaborate.
Response: As suggested, we have rephrased it in the manuscript as follows:
"Contaminated reads containing chloroplast, mitochondrial, bacterial or viral sequences were removed through comparison of the genome assembly with the nucleotide sequence database from the National Center for Biotechnology Information (NCBI) using BLASTN v.2.2.26 with an e-value threshold of ≤ 1e−5."

Question 11: Line 128: "Duplicated genes in" -> Do you mean duplicated haplotigs? Purge_dups operates on scaffolds/contigs, not genes.
Response: Thanks for your careful checks. Based on your comments, we have made the corrections in the manuscript as follows:
"Haplotigs and contig overlaps in the resulting assembly were eliminated using Purge_dups v.1.2.5 (RRID:SCR_021173)."

Question 12: Line 134: "orthologues to the genome by using BUSCO v.5.0" to "orthologues to the genome using BUSCO v5.0"
Response: As suggested, we have made changes in the manuscript.

Question 13: Line 153: "gene model from the other cnidarians" to "gene model from a selection of other cnidarians" -> You are not using all cnidarian genome available, there for you need to make it clear throughout the manuscript for each of your analyses, that you are using a small subset of the genome data available.
Response: Thank you. According to your suggestion, we have rephrased it in the manuscript.

Question 14: Line 159: "combined using the EVM v1.1.1" to "combined using EVM v1.1.1" -> what weights did you use for EVM. Any additional parameters. Please elaborate.
Response: According to the suggestion, we made changes in the manuscript and added the weights of EVM, as follows:
"The weights assigned to ab initio prediction, protein alignment, and transcript were 4, 7, and 8, respectively."

Question 15: Line 177: "results were used to assign the OGs by OrthoFinder v2.4.0" to "results were used by OrthoFinder v2.4.0 to construct OGs"
Response: As suggested, we have made changes in the manuscript.

Question 16: Line 187: Why were OGs with > 100 copies excluded. Please justify this cutoff.
Response: Thanks for this comment. Excluding the orthologue groups greater than 100 copies during gene

family contraction and expansion analysis is a common practice to ensure the accuracy, reliability, and biological relevance of the results. Here are the key reasons for this exclusion:
¬Some OGs may undergo extensive species-specific gene duplication events (e.g., tandem duplications or whole-genome duplications), leading to an unusually high number of copies in a single species.
¬Excluding high-copy gene families helps focus the analysis on orthologs, providing a clearer picture of gene family evolution across species.
¬High-copy gene families may introduce biases in statistical analysis, resulting in distorted results. For example, certain species may exhibit unusually high gene copy numbers for technical reasons (such as differences in the quality of genome assembly), and excluding these gene families can avoid the impact of this technical bias on the overall analysis.

Question 17: Line 236: "sequence similarity protein searches against the NR" -> Provide details on the tools, parameters, and cutoffs used.
Response: According to your suggestion, we have rephrased it in the manuscript as follows:
“Protein annotation was performed by sequence similarity search against the NR database in NCBI and the UniProtKB/SwissProt database using BLASTP with an E-value threshold of 1×10^{-5}.”

Question 18: Line 253: "K-mer-based" -> italicize the "K" in k-mer wherever it is used, it should also be lowercase, unless at the start of a sentence.
Response: Thanks for this comment. We have revised all the “K-mer” in the manuscript to “k-mer”.

Question 19: Line 255: "assembly are 2.67 Mb, while they are 2.61 Mb" to "The contig N50 of the P. papillata assembly is 2.67 Mb and the Chrysogorgia sp. assembly is 2.61 Mb."
Response: As suggested, we have made changes in the manuscript.

Question 20: Line 262: It would be good to also mention the low number of duplicated BUSCO genes, which supports the high quality of the genomes, and your downstream gene family expansion analysis. Additionally, most genome studies compare their assemblies against those currently available, to demonstrate the quality of their new data. While not strictly required, it would be good to mention the quality of the available octocoral data.
Response: As suggested, we rephrased it in the manuscript as follows:
“BUSCO analysis with the metazoan database showed that the genome assemblies of P. papillata and Chrysogorgia sp. contained 91.61% and 88.36% complete BUSCO genes, respectively. Moreover, the proportion of duplicated BUSCO genes in both genomes was 1.89%, which was comparable to that in previously published octocoral genomes (Supplementary Table S6).”

Question 21: Line 266: "were identified in" to "were predicted in"
Response: As suggested, we have made changes in the manuscript.

Question 22: Line 267: "with 91.01% and 90.06% of genes annotated into Nr" -> I do not believe that "annotated" is the correct word here. Just because it has hits to nr does not mean that it is annotated. It has "homology" to other sequences in nr, but annotation implies known function, and many proteins in nr have unknown function. Hits to KEGG and PFAM would be considered "annotated" since they are function-focused databases. Please rephrase.
Response: Thanks for this valuable comment. We rephrased it in the manuscript as follows:
“Among these PCGs, 37,974 (91.01%) in P. papillata and 47,126 (90.06%) in Chrysogorgia sp. were assigned functional annotations by comparing with public databases, including SwissProt, Pfam, NR, TrEMBL, eggNOG, KOG, KEGG, and GO (Supplementary Table S8).”

Question 23: Line 269-270: I would not say that they are "relatively more variable", just more variable than the species that you selected. If you look across all assembled coral genomes, you see a wider variety of sizes (up to 40,000-50,000 genes in some genomes like Montipora capitata or Porites lobata). Consider rephrasing or giving additional context.
Response: Thanks for this valuable comment. Due to our lack of statistics on the protein-coding genes of all published coral genomes, we proposed that such a conclusion was incorrect, so we rephrased it in the manuscript as follows:
“While considerable variation exists in the number of PCGs among different corals, octocoral genomes

typically demonstrate higher counts than hexacoral genomes”

Question 24: Line 270-273: "Genome annotation completeness was also evaluated using BUSCO, and the results showed that the ortholog genes of P. papillata and Chrysogorgia sp. contributed 94.23% and 92.87% of complete genes, respectively (Supplementary Table S10), indicating that our gene annotation is highly complete." to "The BUSCO completeness of the predicted genes in each genome was 94.23% for P. papillata and 92.87% for Chrysogorgia sp. (Supplementary Table S10), indicating that our predicted genes are highly complete."
Response: According to your suggestion, we have made revisions in the manuscript.

Question 25: Line 275-280: "Using a combination of homology-based and de novo approaches, the TEs of 294.04 and 374.71 Mb are identified in the P. papillata and Chrysogorgia sp. genomes (47.58% and 47.98%, respectively) (Fig. 1C; Supplementary Table S11 and S12), with both class II DNA transposons predominate (23.76% and 23.84%, respectively), while class I retrotransposons (long interspersed nuclear elements (LINEs), long terminal repeats (LTRs) and short interspersed nuclear elements (SINEs)) account for 23.81% and 24.14% of the genomes, respectively." to "Using a combination of homology-based and de novo approaches, 294.04 Mb and 374.71 Mb (47.58% and 47.98%) of the P. papillata and Chrysogorgia sp. genomes (respectively) were identified as TEs (Fig. 1C; Supplementary Table S11 and S12), with 23.76% and 23.84% of these TEs being class II DNA transposons and 23.81% and 24.14% of these TEs being class I retrotransposons (long interspersed nuclear elements [LINEs], long terminal repeats [LTRs], and short interspersed nuclear elements [SINEs])."
Response: According to your suggestion, we have made revisions in the manuscript.

Question 26: Line 301: "among the available anthozoan genomes" to "among a selection of the available anthozoan genomes"
Response: According to your suggestion, we have made revisions in the manuscript.

Question 27: Line 423: Normally in a "Data Note" manuscript you would have a concluding paragraph about how good your data is compared to the existing published data, and how it can be used to explore the biology of your system. While this is not required, I recommend that the authors add a brief paragraph summarizing the data presented in the manuscript.
Response: Thanks for this valuable comment. We added the Data Description at the end of the discussion section, as shown below:
Data Description
This study focuses on the assembly, annotation and underlying comparative genomic analyses of two octocoral genomes, P. papillata and Chrysogorgia sp. We further characterized the axial skeletal proteomes of these two octocorals to identify a basic toolkit for coral calcification by comparison with the skeletal proteomes of aragonitic corals. We also found that collagen in the axial skeleton of octocorals has evolved structural domains associated with matrix adhesion and immunity, which may confer new genetic functions for calcification in octocorals. These genomes and proteomes expand the list of octocoral genomes and provide a valuable resource for understanding the molecular mechanisms of coral skeletal formation and their evolutionary history.

Question 28: Line 610: "genome" to "genomes".
Response: According to your suggestion, we have made revisions in the manuscript.

Question 29: Line 619-620: "the estimated divergence time was denoted as blue bar" to "the estimated divergence time at each node is denoted as a blue bar"
Response: According to your suggestion, we have made revisions in the manuscript.

Question 30: Supplementary Figure S3: Why not show the GenomeScope images? Also, "was as the input file of" to "was used as the input file to".
Response: We feel sorry for our carelessness. We performed the survey analysis using Jellyfish software only, not GenomeScope, and we deleted the relevant content in the manuscript and supplementary files.
