# Supplementary material for: The molecular basis of octocoral calcification revealed by genome and skeletal proteome analyses
Source: Gigascience. 2025 Apr 1;14:giaf031. doi: 10.1093/gigascience/giaf031 (PMC11959691; doi:10.1093/gigascience/giaf031)
Supplement: giaf031_Reviewer_1_Report_Original_Submission — Eldon Ball -- 12/16/2024 [file giaf031_reviewer_1_report_original_submission.pdf]

Reviewer Report

Title: The molecular basis of octocoral calcification revealed by genome and skeletal proteome analyses

Version: Original Submission **Date:** 12/16/2024

Reviewer name: Eldon Ball

Reviewer Comments to Author:

The revised paper is much better focused than the previous version. It presents 2 relatively high quality (judging by the BUSCO scores) octocoral genomes and analyzes them using standard methods. It also presents proteomic data for the same 2 species. These genomes and proteomes are a welcome addition to the literature as there have previously been only a few genomes or proteomes for this group. Genome compositions are also reported for components such as transposable elements. The new genomes have then been combined with previously reported scleratinian and octocorallian datasets to determine a common biomineralization toolkit for the two groups. Anatomical and histochemical comparisons between members of the two groups are also reported. Materials, methods and analyses are clearly reported and appear appropriate to me, although I cannot claim to be an expert in bioinformatics. In spite of a general improvement, several points that I previously raised have not been corrected.* I think there should be a clear statement of whether either of these species has calcified spicules in addition to an axial skeleton. From the manuscript it appears that neither species has spicules but because they are common in octocorals this should be clarified.* the same sorts of grammatical mistakes that I pointed out previously are still present.There are many instances where the articles "the" and "a" are needed and where singular and plural are incorrectly used. For example, in the first sentence of the abstract there is an example of the latter mistake. The word "ability" is singular and determines the verb, which should be "has" not "have".* to repeat my previous comment: "On a related note, the authors are very inconsistent in their use of genus names, sometimes spelling them out and sometimes abbreviating them. The traditional solution to this problem is to spell out genus and species in full on first use (e.g Acropora millepora) and thereafter use an abbreviation (e.g. A. millepora)."yet on lines 153-155 the authors still have" A.digitifera, A. millepora, Astreopora myriophthalma, D. gigantea, Porites australiensis, P. clavata,Stylophora postillata." Is there a reason that they don't want to follow the usual convention?*The reply of the authors to question 10 from reviewer 2 is an excellent, succinct summary of their work , which perhaps could be incorporated into their paper. "we focus on the assembly, annotation and underlying comparative genomic analyses of two octocorals. Skeletal proteome analyses led us to identify a basic toolkit for coral calcification. We further found that collagen in the skeleton of octocorals has evolved structural domains associated with matrix adhesion and immunity, which may confer new genetic functions for calcification in octocorals. These genomes and proteomes expand the list of octocoral genomes and provide a valuable resource for understanding the molecular mechanisms of coral skeletal formation and their evolutionary history."* Suppl Table S1 is a very useful addition to the paper* I was unable to view Tables S13-S18. Following S12 there is a page with the following:Table

S13-S18 in the Supplementary information section* The captions to the Supplementary figures could use English editing

Level of Interest

Please indicate how interesting you found the manuscript: Choose an item.

Quality of Written English

Please indicate the quality of language in the manuscript: Choose an item.

Declaration of Competing Interests

Please complete a declaration of competing interests, considering the following questions:

- Have you in the past five years received reimbursements, fees, funding, or salary from an organisation that may in any way gain or lose financially from the publication of this manuscript, either now or in the future?
- Do you hold any stocks or shares in an organisation that may in any way gain or lose financially from the publication of this manuscript, either now or in the future?
- Do you hold or are you currently applying for any patents relating to the content of the manuscript?
- Have you received reimbursements, fees, funding, or salary from an organization that holds or has applied for patents relating to the content of the manuscript?
- Do you have any other financial competing interests?
- Do you have any non-financial competing interests in relation to this paper?

If you can answer no to all of the above, write 'I declare that I have no competing interests' below. If your reply is yes to any, please give details below.

I declare that I have no competing interests

I agree to the open peer review policy of the journal. I understand that my name will be included on my report to the authors and, if the manuscript is accepted for publication, my named report including any attachments I upload will be posted on the website along with the authors' responses. I agree for my report to be made available under an Open Access Creative Commons CC-BY license (http://creativecommons.org/licenses/by/4.0/). I understand that any comments which I do not wish to be included in my named report can be included as confidential comments to the editors, which will not be published.

Choose an item.

To further support our reviewers, we have joined with Publons, where you can gain additional credit to further highlight your hard work (see: https://publons.com/journal/530/gigascience). On publication of this paper, your review will be automatically added to Publons, you can then choose whether or not to claim your Publons credit. I understand this statement.

Yes Choose an item.
