# Supplementary material for: The molecular basis of octocoral calcification revealed by genome and skeletal proteome analyses
Source: Gigascience. 2025 Apr 1;14:giaf031. doi: 10.1093/gigascience/giaf031 (PMC11959691; doi:10.1093/gigascience/giaf031)
Supplement: giaf031_Reviewer_2_Report_Original_Submission — Timothy Gordon Stephens, Ph.D. -- 1/28/2025 [file giaf031_reviewer_2_report_original_submission.pdf]

Reviewer Report

Title: The molecular basis of octocoral calcification revealed by genome and skeletal proteome analyses

Version: Original Submission **Date:** 1/28/2025

Reviewer name: Timothy Gordon Stephens, Ph.D.

Reviewer Comments to Author:

The manuscript by Liang et al. detail the high-quality genome assemblies of Paragorgia papillata and Chrysogorgia sp., two octocorals of ecological and evolutionary significance, and the comparison of these genomes against a select group of hard corals. The authors also present the skeletal proteomes of these two species, elucidating the complex matrix structure of these corals and connecting it to the generated genomes. This manuscript is well written and represents a valuable contribution to the field. I have some comments below for the authors to address but look forward to seeing this research published.Line 20: "have led to their" to "has led to their"Lines 25-27: "Both assembled genomes size is 618.13 Mb and 781.04 Mb for P. papillata and Chrysogorgia sp. respectively, with contig N50 of 2.67 Mb and 2.61 Mb" to "The assembled genomes sizes were 618.13 Mb and 781.04 Mb for P. papillata and Chrysogorgia sp., respectively, with contig N50s of 2.67 Mb and 2.61 Mb"Line 43: Is this what you were trying to say? "Biomineralization of CaCO3 originated in organisms at least 541 Mya"Line 46: "biogenic structures by their ability" to "biogenic structures through their ability"Line 48: "CaCO3 skeleton-producing ability is found" to "The ability to produce CaCO3 skeletons is found"Line 58: "of diverse organic matrix to calcification site" to "of a diverse organic matrix at the site of calcification"Line 59: "Major components of organic matrix include proteins" to "Major components of the organic matrix include proteins"Line 103-104: "This was followed by extending, gap filling and polishing the assembly" -> What tools and parameters were used for this? Was it SOAPdenovo or something else? Please elaborate.Line 120: "Following the removal of low-quality and duplicate reads" -> Using what tools and parameters? Please elaborate.Line 123: "sequences were removed via comparison of the genome assembly" -> Using what tools and parameters? I assume BLASTN, but using what cutoffs? Please elaborate.Line 128: "Duplicated genes in" -> Do you mean duplicated haplotigs? Purge_dups operates on scaffolds/contigs, not genes.Line 134: "orthologues to the genome by using BUSCO v.5.0" to "orthologues to the genome using BUSCO v5.0"Line 153: "gene model from the other cnidarians" to "gene model from a selection of other cnidarians" -> You are not using all cnidarian genome available, there for you need to make it clear throughout the manuscript for each of your analyses, that you are using a small subset of the genome data available.Line 159: "combined using the EVM v1.1.1" to "combined using EVM v1.1.1" -> what weights did you use for EVM. Any additional parameters. Please elaborate.Line 177: "results were used to assign the OGs by OrthoFinder v2.4.0" to "results were used by OrthoFinder v2.4.0 to construct OGs"Line 187: Why were OGs with > 100 copies excluded. Please justify this cutoff.Line 236: "sequence similarity protein searches against the NR" -> Provide details on the tools, parameters, and cutoffs used.Line 253: "K-mer-based" -> italicize the "K" in k-mer wherever it is used, it should also be lowercase, unless at the start of a sentence.Line 255: "assembly are 2.67 Mb,

while they are 2.61 Mb" to "The contig N50 of the P. papillata assembly is 2.67 Mb and the Chrysogorgia sp. assembly is 2.61 Mb."Line 262: It would be good to also mention the low number of duplicated BUSCO genes, which supports the high quality of the genomes, and your downstream gene family expansion analysis. Additionally, most genome studies compare their assemblies against those currently available, to demonstrate the quality of their new data. While not strictly required, it would be good to mention the quality of the available octocoral data.Line 266: "were identified in" to "were predicted in"Line 267: "with 91.01% and 90.06% of genes annotated into Nr" -> I do not believe that "annotated" is the correct word here. Just because it has hits to nr does not mean that it is annotated. It has "homology" to other sequences in nr, but annotation implies known function, and many proteins in nr have unknown function. Hits to KEGG and PFAM would be considered "annotated" since they are function-focused databases. Please rephrase.Line 269-270: I would not say that they are "relatively more variable", just more variable than the species that you selected. If you look across all assembled coral genomes, you see a wider variety of sizes (up to 40,000-50,000 genes in some genomes like Montipora capitata or Porites lobata). Consider rephrasing or giving additional context.Line 270-273: "Genome annotation completeness was also evaluated using BUSCO, and the results showed that the ortholog genes of P. papillata and Chrysogorgia sp. contributed 94.23% and 92.87% of complete genes, respectively (Supplementary Table S10), indicating that our gene annotation is highly complete." to "The BUSCO completeness of the predicted genes in each genome was 94.23% for P. papillata and 92.87% for Chrysogorgia sp. (Supplementary Table S10), indicating that our predicted genes are highly complete."Line 275-280: "Using a combination of homology-based and de novo approaches, the TEs of 294.04 and 374.71 Mb are identified in the P. papillata and Chrysogorgia sp. genomes (47.58% and 47.98%, respectively) (Fig. 1C; Supplementary Table S11 and S12), with both class II DNA transposons predominate (23.76% and 23.84%, respectively), while class I retrotransposons (long interspersed nuclear elements (LINEs), long terminal repeats (LTRs) and short interspersed nuclear elements (SINEs)) account for 23.81% and 24.14% of the genomes, respectively." to "Using a combination of homology-based and de novo approaches, 294.04 Mb and 374.71 Mb (47.58% and 47.98%) of the P. papillata and Chrysogorgia sp. genomes (respectively) were identified as TEs (Fig. 1C; Supplementary Table S11 and S12), with 23.76% and 23.84% of these TEs being class II DNA transposons and 23.81% and 24.14% of these TEs being class I retrotransposons (long interspersed nuclear elements [LINEs], long terminal repeats [LTRs], and short interspersed nuclear elements [SINEs])."Line 301: "among the available anthozoan genomes" to "among a selection of the available anthozoan genomes"Line 423: Normally in a "Data Note" manuscript you would have a concluding paragraph about how good your data is compared to the existing published data, and how it can be used to explore the biology of your system. While this is not required, I recommend that the authors add a brief paragraph summarizing the data presented in the manuscript.Line 610: "genomes" to "genomes".Line 619-620: "the estimated divergence time was denoted as blue bar" to "the estimated divergence time at each node is denoted as a blue bar"Supplementary Figure S3: Why not show the GenomeScope images? Also, "was as the input file of" to "was used as the input file to".

Level of Interest

Please indicate how interesting you found the manuscript: Choose an item.

Quality of Written English

Please indicate the quality of language in the manuscript: Choose an item.

Declaration of Competing Interests

Please complete a declaration of competing interests, considering the following questions:

- Have you in the past five years received reimbursements, fees, funding, or salary from an organisation that may in any way gain or lose financially from the publication of this manuscript, either now or in the future?
- Do you hold any stocks or shares in an organisation that may in any way gain or lose financially from the publication of this manuscript, either now or in the future?
- Do you hold or are you currently applying for any patents relating to the content of the manuscript?
- Have you received reimbursements, fees, funding, or salary from an organization that holds or has applied for patents relating to the content of the manuscript?
- Do you have any other financial competing interests?
- Do you have any non-financial competing interests in relation to this paper?

If you can answer no to all of the above, write 'I declare that I have no competing interests' below. If your reply is yes to any, please give details below.

I declare that I have no competing interests'

I agree to the open peer review policy of the journal. I understand that my name will be included on my report to the authors and, if the manuscript is accepted for publication, my named report including any attachments I upload will be posted on the website along with the authors' responses. I agree for my report to be made available under an Open Access Creative Commons CC-BY license (http://creativecommons.org/licenses/by/4.0/). I understand that any comments which I do not wish to be included in my named report can be included as confidential comments to the editors, which will not be published.

Choose an item.

To further support our reviewers, we have joined with Publons, where you can gain additional credit to further highlight your hard work (see: https://publons.com/journal/530/gigascience). On publication of this paper, your review will be automatically added to Publons, you can then choose whether or not to claim your Publons credit. I understand this statement.

Yes Choose an item.
